# Unveiling the PDK4-centered rituximab-resistant mechanism in DLBCL: the potential of the “Smart” exosome nanoparticle therapy

**DOI:** 10.1186/s12943-024-02057-0

**Published:** 2024-07-15

**Authors:** Xin Wu, Chunmei Ban, Woding Deng, Xuewei Bao, Ning Tang, Yupeng Wu, Zhixuan Deng, Jianbin Xiong, Qiangqiang Zhao

**Affiliations:** 1 Department of Spine Surgery, Third Xiangya Hospital, Central South University, Changsha, Hunan China; 2 Department of Hematology, Liuzhou People’s Hospital affiliated to Guangxi Medical University, Liuzhou, Guangxi China; 3 Xiangya School of Medicine, Central South University, Changsha, China; 4 Department of Hematology, The Qinghai Provincial People’s Hospital, Xining, Qinghai China; 5 Department of Orthopedics, Third Xiangya Hospital, Central South University, Changsha, Hunan China; 6 Department of Spine Surgery, First Affiliated Hospital of University of South China, Hengyang, Hengyang, Hunan China; 7 Institute of Cell Biology, Hengyang Medical School, University of South China, Hengyang, Hengyang, Hunan China; 8 Department of Orthopaedics, Liuzhou Municipal Liutie Central Hospital, Liuzhou, Guangxi China

**Keywords:** Diffuse large B-cell lymphoma, PDK4, Rituximab resistance, Advanced exosome nanoparticles, Targeted therapy, Immunotherapy

## Abstract

**Background:**

Diffuse large B-cell lymphoma (DLBCL) represents a prevalent malignant tumor, with approximately 40% of patients encountering treatment challenges or relapse attributed to rituximab resistance, primarily due to diminished or absent CD20 expression. Our prior research identified PDK4 as a key driver of rituximab resistance through its negative regulation of CD20 expression. Further investigation into PDK4’s resistance mechanism and the development of advanced exosome nanoparticle complexes may unveil novel resistance targets and pave the way for innovative, effective treatment modalities for DLBCL.

**Methods:**

We utilized a DLBCL-resistant cell line with high PDK4 expression (SU-DHL-2/R). We infected it with short hairpin RNA (shRNA) lentivirus for RNA sequencing, aiming to identify significantly downregulated mRNA in resistant cells. Techniques including immunofluorescence, immunohistochemistry, and Western blotting were employed to determine PDK4’s localization and expression in resistant cells and its regulatory role in phosphorylation of Histone deacetylase 8 (HDAC8). Furthermore, we engineered advanced exosome nanoparticle complexes, aCD20@Exo^CTX^/siPDK4, through cellular, genetic, and chemical engineering methods. These nanoparticles underwent characterization via Dynamic Light Scattering (DLS) and Transmission Electron Microscopy (TEM), and their cellular uptake was assessed through flow cytometry. We evaluated the nanoparticles’ effects on apoptosis in DLBCL-resistant cells and immune cells using CCK-8 assays and flow cytometry. Additionally, their capacity to counteract resistance and exert anti-tumor effects was tested in a resistant DLBCL mouse model.

**Results:**

We found that PDK4 initiates HDAC8 activation by phosphorylating the Ser-39 site, suppressing CD20 protein expression through deacetylation. The aCD20@Exo^CTX^/siPDK4 nanoparticles served as effective intracellular delivery mechanisms for gene therapy and monoclonal antibodies, simultaneously inducing apoptosis in resistant DLBCL cells and triggering immunogenic cell death in tumor cells. This dual action effectively reversed the immunosuppressive tumor microenvironment, showcasing a synergistic therapeutic effect in a subcutaneous mouse tumor resistance model.

**Conclusions:**

This study demonstrates that PDK4 contributes to rituximab resistance in DLBCL by modulating CD20 expression via HDAC8 phosphorylation. The designed exosome nanoparticles effectively overcome this resistance by targeting the PDK4/HDAC8/CD20 pathway, representing a promising approach for drug delivery and treating patients with Rituximab-resistant DLBCL.

**Supplementary Information:**

The online version contains supplementary material available at 10.1186/s12943-024-02057-0.

## Introduction

Lymphomas constitute a group of malignant tumors originating from the lymphatic hematopoietic system, with non-Hodgkin lymphoma (NHL) comprising approximately 90% of these conditions [1]. Within this category, diffuse large B-cell lymphoma (DLBCL) accounts for 35–40% of NHL instances [2]. Despite the R-CHOP regimen (rituximab, cyclophosphamide, doxorubicin, vincristine, and prednisone) enhancing DLBCL patient outcomes, around 40% of individuals advance to a relapsed or refractory stage owing to chemotherapy resistance [3]. Furthermore, a minority, less than 20%, of these relapsed or refractory patients derive benefit from hematopoietic stem cell transplantation [4]. While CAR-T cell therapy has been efficacious in treating relapsed/refractory DLBCL, its associated high costs, adverse effects, and the potential for patient relapse presents significant challenges [5]. The propensity for DLBCL patient relapse has been closely linked to rituximab’s clinical effectiveness, with resistance to this drug posing a persistent obstacle in treatment [6]. As such, unveiling the resistance mechanisms to rituximab and innovating novel therapeutic approaches to counteract this resistance remains a pivotal area of research.

The CD20 expression levels heavily influence the efficacy of rituximab in treating DLBCL in lymphoma cells. Research has demonstrated that patients exhibiting high CD20 expression levels and sensitivity to rituximab can, following treatment, show reduced CD20 expression or even become CD20-negative, leading to acquired resistance [7]. Our study utilized RNA-Seq sequencing to compare gene expression between rituximab-sensitive and -resistant patients, revealing a significant upregulation of pyruvate dehydrogenase kinase 4 (PDK4) in the latter group. Subsequent in vitro and in vivo studies have indicated that PDK4 plays a role in mediating DLBCL’s resistance to rituximab by downregulating CD20 expression [8] despite the several benefits of targeted therapy using small interfering RNA (siRNA), challenges such as siRNA’s short half-life, poor membrane penetration, rapid degradation in circulation, and low stability persist [9]. Therefore, the development of an effective delivery vehicle is crucial for the successful administration of PDK4 siRNA.

Exosomes (Exo), bilayer nanovesicles ranging from 30 to 150 nm, exhibit excellent biocompatibility in vivo [10]. Research indicates that exogenous oligonucleotides, such as siRNA, can be efficiently encapsulated into exosomes via electroporation, safeguarding siRNA against degradation and ensuring its intact delivery to target cells [11]. Furthermore, exosomes originating from bone marrow mesenchymal stem cells (BMSCs) retain specific characteristics of the progenitor cells, including the inherent capacity for tumor homing, rendering them effective natural nanocarriers [12, 13]. However, the diminished or absent expression of the CD20 antigen in patients with relapsed/refractory diffuse large B-cell lymphoma (DLBCL) and resistant cells complicates the targeting of lymphoma cells using Rituximab (RTX) [14]. DSPE-PEG, a lipid-like material, is commonly employed for its ability to integrate functional molecules onto the exosomal surface through hydrophobic interactions with membrane lipids [15] while also imparting “stealth” properties to exosomes, minimizing protein adsorption and enhancing tumor targeting and drug delivery [16]. Although BMSC-derived exosomes are produced in relatively high yields, BMSCs exhibit potent immunosuppressive effects, including inhibiting T cell activation and dendritic cell maturation and promoting regulatory T cell proliferation and differentiation [17]. Cyclophosphamide (CTX), a critical chemotherapeutic agent in the R-CHOP regimen, can suppress Treg cell function and induce immunogenic cell death at low doses, bolstering antitumor activity [18]. ICD is a unique form of apoptosis that can activate the immune system to recognize and eliminate tumor cells. During this process, dendritic cells (DCs) play a central role. They capture and process antigens released by dying tumor cells, subsequently activating T cells and initiating a specific immune response against the tumor. Nonetheless, CTX’s antitumor efficacy is limited by its poor pharmacokinetics, low tumor accumulation, and nonspecific toxicity toward healthy tissue and immune cells [19]. Studies suggest that exosomes obtained after co-incubating drugs with parent cells encapsulate the drugs, showing enhanced antitumor effects compared to free drugs [20]. BMSCs demonstrate low sensitivity to various chemotherapeutics, including cyclophosphamide, indicating a broad tolerance to chemotherapy drugs [21]. This approach addresses the challenge of delivering hydrophobic drugs and amplifies antitumor efficacy through exosomes modified by cellular engineering.

**Fig. 1:**
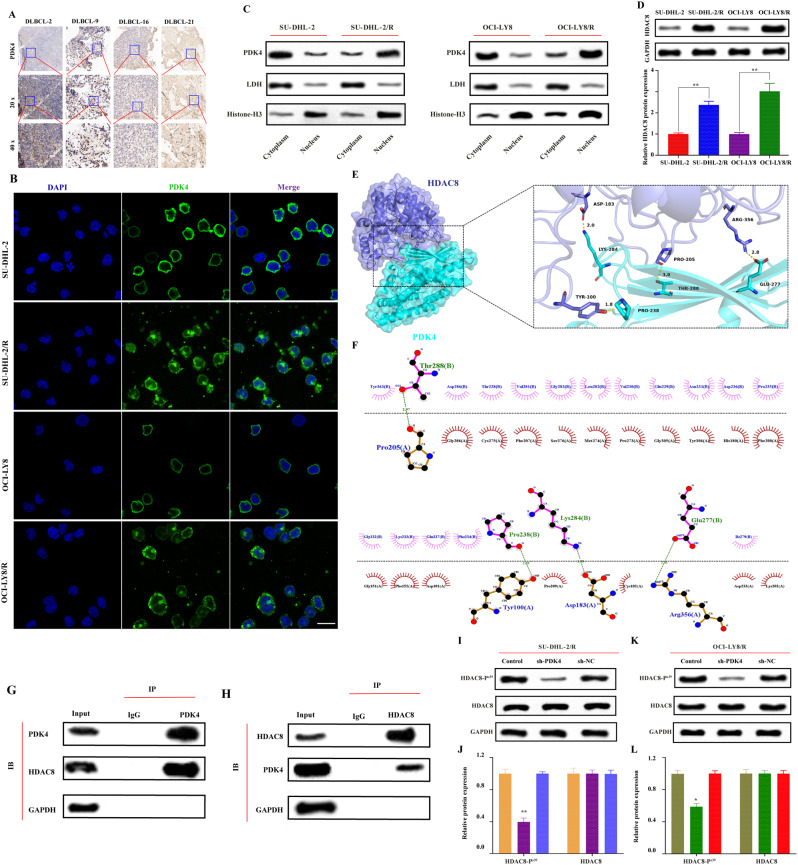
(**A**) Immunohistochemistry validates the localization of PDK4 in DLBCL chemotherapy-sensitive and chemotherapy-resistant patient tissues (DLBCL-2 represents chemotherapy-sensitive patients, DLBCL-9, DLBCL-16, and DLBCL-21 represent chemotherapy-resistant patients); (**B**) Immunofluorescence demonstrates the cellular localization of PDK4 in DLBCL resistant cells and parent cells, where green fluorescence indicates the cellular localization of PDK4, and blue fluorescence indicates the nuclear localization of DAPI. The scale bar is 20 μm; (**C**) Cytoplasmic and nuclear fractionation, Western blot analysis of PDK4 expression and localization in sensitive and resistant cell lines, with LDH and Histone-H3 used as cytoplasmic and nuclear markers, respectively. (**D**) Western Blot analysis of HDAC8 protein expression levels and Statistical chart of bands by Image J software. **vs sh-NC: *P* < 0.01; (**E**) Analysis of the protein-protein interaction interface between HDAC8 and PDK4 based on structure. The image represents the predicted structure of the HDAC8-PDK4 complex, where interactive hot spot residues are marked. (**F**) Two-dimensional angle analysis and visualization of the interaction forces between HDAC8 and PDK4 proteins. The 205th Pro amino acid residue, the 100th Tyr amino acid residue, the 183rd Asp amino acid residue, and the 356th Arg amino acid residue of the HDAC8 protein can bind through hydrogen bonds with the 288th Thr amino acid residue, the 238th Pro amino acid residue, the 284th Lys amino acid residue, and the 277th Glu amino acid residue of the PDK4 protein, with lengths of 2.97Å, 1.83Å, 1.99Å, and 2.81Å, respectively; in the two-dimensional graph, green dashed lines represent hydrogen bonds. (**G**) In SU-DHL-2/R cells, PDK4 was immunoprecipitated using an anti-PDK4 antibody, and then HDAC8 was detected by a western blotting assay. The control immunoprecipitation was conducted with IgG. (**H**) In SU-DHL-2/R cells, HDAC8 was immunoprecipitated using an anti-HDAC8 antibody, and then PDK4 was detected by a western blotting assay. The control immunoprecipitation was conducted with IgG. (**I**, **K**) Western blot confirms that downregulation of PDK4 (sh-PDK4) can inhibit the phosphorylation of HDAC8 protein but does not affect the expression of HDAC8 protein, GAPDH is the internal reference for protein loading; (**J**, **L**) Statistical chart of bands by Image J software. *vs sh-NC or Control: *P* < 0.05; **vs sh-NC or Control: *P* < 0.01

This study proposes developing a “smart”exosome-targeted delivery strategy to overcome PDK4-mediated resistance to rituximab in DLBCL. Figure 1A illustrates the process: initially, cyclic phosphoramidate is co-incubated with BMSCs, yielding exosomes (Exo^CTX^) via ultracentrifugation. Subsequently, Exo^CTX^ and PDK4 siRNA are separately introduced into an electroporation cup, facilitating the creation of exosome nanoparticles (Exo^CTX^/siPDK4) through electroporation. In the final step, DSPE-Hyd-PEG2000-NHS conjugates with rituximab to generate a pioneering compound (aCD20), which is integrated into the membrane of the exosome nanoparticles, resulting in the formation of a novel exosomal nanoparticle medication, referred to as aCD20@Exo^CTX^/siPDK4.

## Materials and methods

### Materials

RPMI 1640 medium (Gibico), DMEM medium (Gibico, USA); Fetal Bovine Serum (Gibco, USA); PBS (Servicebio); Exosome-depleted serum (Umibio); DMSO (Solabio), CCK-8 assay kit (Dojindo, Japan); Reactive Oxygen Species assay kit (Beyotime, China); PKH67 (Sigma, USA); Calcein-AM/propidium iodide (PI) was purchased from Yeasen Biotechnology (China). The apoptosis detection kit was purchased from BD Biosciences (USA). Rituximab (TOPSCIENCE), Cyclophosphamide (TOPSCIENCE), Inhibitor PCI-34,051 (Selleck), Cytoplasm/Nucleus Separation Kit (Invitrogen), TRIzol (Invitrogen), RT-PCR Kit (Takara), PCR Primers (Sangon Biotech), SDS-PAGE Gel Preparation Kit (Beyotime, Wuhan); EasySep™ Mouse T Cell Isolation Kit was purchased from STEMCELL Technologies. Anti-GAPDH antibody (CST), Anti-PDK4 antibody (Abcam), Anti-HDAC8 antibody (CST), Anti-HDAC8 (phospho S39) antibody (Abcam), Anti-Histone H3 antibody (CST), Anti-ac-Histone H3 antibody (CST), Anti-LDH antibody (Abcam), Anti-CD20 antibody (Abcam), Anti-CD90 antibody (Abcam), Anti-CD73 antibody (Abcam), Anti-CD81 antibody (CST), Anti-CD63 antibody (CST), Anti-TSG101 antibody (CST), Anti-Calnexin antibody (CST), Anti-Bcl-2 antibody (CST), Anti-PARP-1 antibody (CST), Anti-Cleaved PARP-1 antibody (CST), Anti-Caspase-3 antibody (CST), Anti-cleaved Caspase-3 antibody (CST), Anti-Tubulin antibody (CST), Secondary antibodies for Western Blot (CST), Secondary antibodies for immunofluorescence (ThermoFisher). An anti-calreticulin (CRT) antibody was purchased from Abcam. The human HMGB1 ELISA Kit and ATP assay kit were purchased from Beyotime Biotechnology. anti-CD11c anti-CD86 were purchased from CST. anti-CD80, anti-CD3, anti-CD4, anti-Foxp3 and anti-CD8 were purchased from Sigma-Aldrich. RNAiso Plus was purchased from TAKARA. TNF-α, IFN-γ, IL-12, and IL-6 ELISA kits were purchased from NEOBIOSCIENCE.

### Cell culture

The human DLBCL cell lines SU-DHL-2 and OCI-Ly8 were obtained from the Cancer Research Institute of Central South University. The rituximab-resistant cell lines SU-DHL-2/R and OCI-ly8/R were successfully constructed and validated by our team based on previous reports [8, 22]. Cells were cultured in RPMI 1640 medium (90% 1640, 10% FBS, 1% penicillin, and streptomycin) and incubated in a cell culture incubator at 37 °C with 5% CO_2_. The cells were passaged every two days, and once they reached the logarithmic growth phase, they were used for the studies in the following experiments.

### Preparation and characterization of aCD20@Exo^CTX^/siPDK4 nanoparticles

The preparation process of aCD20@Exo^CTX^/siPDK4 **(**Fig. 1A**)** begins with co-incubating cyclophosphamide with BMSCs and obtaining exosomes (Exo^CTX^) through ultracentrifugation. Subsequently, Exo^CTX^ and PDK4 siRNA are added into an electroporation cuvette, and exosome nanoparticles (Exo^CTX^/siPDK4) are prepared using the electroporation method. Finally, a precursor compound (aCD20) is formed by coupling rituximab with DSPE-Hyd-PEG2000-NHS, and it is inserted into the membrane of the exosome nanoparticles to construct a novel exosomal nanoparticle drug (named aCD20@Exo^CTX^/siPDK4). For TEM characterization, purified Exo, Exo^CTX^, and aCD20@Exo^CTX^/siPDK4 solutions were dropped onto a carbon-coated copper grid and stained with 2% uranyl acetate. The transmission electron microscope was used at 75 kV to observe samples. Nanosight NS300 was used to test the hydrodynamic diameters and zeta potential of suspended purified Exo, Exo^CTX^, aCD20@Exo^CTX^/siPDK4. The infrared spectral scanning range was 4000 ~ 400 cm^− 1^ with a resolution of 4 cm^− 1^, accumulating 16 scans to eliminate the interference of water and CO2. Each sample was scanned three times, and the average spectrum for each sample was obtained using OMNIC 8.2 software. The average spectrum was subjected to baseline correction, 13-point smoothing, and normalization. The processed spectrum was then differentiated to get the second derivative spectrum.

### Laser confocal microscopy and flow cytometry analysis of cell uptake of nanoparticle aCD20@Exo^CTX^/siPDK4

To visualize the internalization of the exosomal nanoparticle aCD20@Exo^CTX^/siPDK4, 4 µL of PKH67 was diluted in 1 mL of diluent C to prepare the working solution according to the manufacturer’s instructions. The green fluorescent dye PKH67 working solution was mixed with the resuspended exosome aCD20@Exo^CTX^/siPDK4 and incubated in the dark for 5 min, followed by the addition of 1 mL of exosome-depleted serum to terminate the staining. After ultracentrifugation at 100,000×g for 70 min, PKH67-labeled exosomes aCD20@Exo^CTX^/siPDK4 were obtained [23]. Subsequently, cells SU-DHL-2 and SU-DHL-2/R were incubated with various concentrations of the labeled exosomal nanoparticles aCD20@Exo^CTX^/siPDK4 or at different time points with 2 × 10^5^ cells in 24-well plates. After co-culture, cells were washed three times with pre-cooled PBS. Cells were resuspended in PBS and vortexed, and the cellular fluorescence intensity was measured using a flow cytometer. The remaining cells were smeared onto slides and air-dried, then fixed with 4% paraformaldehyde at room temperature for 15 min. Cells were stained with DAPI staining solution at 37 °C for 15 min; the supernatant was removed and washed three times with PBS, each time for 5 min. The cell smears were air-dried, sealed with anti-fluorescence quenching mounting medium, and observed under a laser confocal microscope (Nikon, ECLIPSE TI) for fluorescence and photographed.

### Assessment of cell proliferation and viability using the CCK-8 assay and calcein-AM/PI double staining method

Cells were collected and counted, and the number of SU-DHL-2 and SU-DHL-2/R cells was adjusted to 2 × 10^^4^/ml. Subsequently, PBS, RTX, RTX + siPDK4, aCD20@Exo/siPDK4, and aCD20@Exo^CTX^/siPDK4 were added, followed by thorough mixing. 100 µl of the cell suspension was plated into each well of a 96-well plate, and 100 µl of the medium was added to the wells to serve as blank controls. Each group had three replicate wells. After 24 h of incubation, ten µL of CCK-8 solution was added to each well, and the plate was further incubated at 37 °C in 5% CO_2_ for 1 to 4 hours. The optical density (OD) at 450 nm wavelength was measured using a microplate reader (PerkinElmer EnSpire, USA). For Calcein-AM/PI staining, SU-DHL-2 or SU-DHL-2/R cells were seeded in 12-well plates at a density of 1 × 10^4 cells/well. PBS, RTX, RTX + siPDK4, RTX@Exo/siPDK4, and RTX@Exo^CTX^/siPDK4 were added, and after 24 h of incubation, the cells were stained with calcein AM and propidium iodide (PI). The fluorescent images of the cells were captured using an inverted fluorescence microscope (ZEISS Axio Vert.A1, Germany).

### Detection of cell apoptosis by flow cytometry

SU-DHL-2 and SU-DHL-2/R cells were plated in 6-well plates (1 × 10^^5^ cells/well) and treated with PBS, RTX, RTX + siPDK4, aCD20@Exo/siPDK4, and aCD20@Exo^CTX^/siPDK4 for 24 h. The cells were centrifuged and washed twice with PBS, and the supernatant was discarded. Subsequently, 100 µl of 1× binding buffer was added to resuspend the cells, followed by 5 µl of Annexin V-FITC. The mixture was gently mixed, then ten µl of propidium iodide staining solution was added and mixed gently. The cells were incubated in the dark at room temperature for 10–20 min, then resuspended in 200 µl of binding buffer for immediate analysis by flow cytometry.

### Detection of cellular protein expression levels by Western blot method

SU-DHL-2 and SU-DHL-2/R cells were seeded in T25 flasks (1 × 10^6 cells/flask) and treated with PBS, RTX, RTX + siPDK4, aCD20@Exo/siPDK4, and aCD20@Exo^CTX^/siPDK4. The cells were cultured at 37 °C in a 5% CO2 environment for 24 h. After centrifugation, the supernatant was discarded, and the cells were resuspended in PBS, followed by another round of centrifugation and discarding of the supernatant. 1× loading buffer was added to the cell pellet, which was then boiled for 8 min to prepare the protein samples and stored at -20 °C. Gels were prepared according to the molecular weight of the proteins of interest, and protein samples, along with a marker, were loaded for electrophoresis. The membrane transfer time was adjusted based on the molecular weights of the proteins. The transferred PVDF membrane was blocked in milk on a shaker at room temperature for 1.5 h, followed by overnight incubation with the primary antibody at 4 °C. The PVDF membrane was washed three times with PBS, incubated with the secondary antibody on a shaker at room temperature for 1.5 h, and then washed three times with PBS. An ECL solution was prepared at a 1:1 ratio and applied to the PVDF membrane, and the membrane was exposed and scanned for imaging in a chemiluminescence gel imaging system.

### In Vitro Validation of aCD20@Exo^CTX^/siPDK4 nanoparticles inducing immunogenic cell death (ICD) in SU-DHL-2/R cells

SU-DHL-2/R cells were seeded in a 6-well plate at 1 × 10^4 cells per well and cultured overnight. Subsequently, PBS, RTX, RTX + siPDK4, aCD20@Exo/siPDK4, and aCD20@Exo^CTX^/siPDK4 were added to the wells. After 4 h of incubation, the culture medium was removed, and the cells were washed twice with PBS. The cells were then collected and centrifuged at 1000 rpm for 5 min. The supernatant was discarded, and the cells were resuspended in 0.1 mL PBS. Alexa Fluor^®^ 488 labeled CRT antibody was added for staining for 24 h [24]. After 45 min of incubation, the cells were washed twice with PBS and analyzed by flow cytometry. Using an ELISA kit to determine the release of HMGB1 from SU-DHL-2/R cells following various treatments [25]: First, SU-DHL-2/R cells are seeded in a 6-well plate at a density of 5 × 10^4 cells per well and cultured overnight. PBS, RTX, RTX + siPDK4, aCD20@Exo/siPDK4, and aCD20@Exo^CTX^/siPDK4 are added to the wells. After 24 h of incubation, the supernatant is collected and centrifuged at 3000 rpm for 10 min to remove debris, and the samples are transferred to centrifuge tubes for storage. Standards are prepared, and 50 µL of either standard or sample, along with 50 µL of sample diluent and 100 µL of HMGB1 antibody, are added to each well and incubated at 4 °C in the dark for 16 h. After incubation, the liquid in the wells is discarded, and the wells are washed 4 times with wash buffer. The substrate solution is added for 20 min, followed by a stop solution to end the reaction. The absorbance at 450 nm is measured using a microplate reader. To measure the release of ATP from SU-DHL-2/R cells treated with aCD20@Exo^CTX^/siPDK4, an ATP assay kit is used following these steps: [25]: First, SU-DHL-2/R cells are plated in a 6-well plate with 5 × 10^4 cells per well. After culturing for 12 h, the experimental groups are treated with PBS, RTX, RTX + siPDK4, aCD20@Exo/siPDK4, and aCD20@Exo^CTX^/siPDK4 in the six-well plate. After 24 h of incubation, the supernatant is collected and centrifuged at 3000 rpm for 10 min to remove debris, and the samples are stored in centrifuge tubes. ATP standards are then prepared according to the instructions. The ATP detection working solution is prepared as directed in the manual. The ATP detection working solution is added to Eppendorf tubes, and then the samples or standards are added. The chemiluminescence is measured using a luminometer. This process quantifies ATP release from the cells, indicating cellular metabolic activity and potential treatment responses.

### In vitro extraction, culture, and maturation of BMDCs induced by aCD20@Exo^CTX^/siPDK4

Bone marrow dendritic cells (BMDCs) were extracted from C57BL/6 mice using the method described previously [26]. In brief, leukocytes collected from the bone marrow were seeded in six-well plates (1 × 10^6/well). Then, five ng/ml of interleukin-4 (IL-4) and 20 ng/ml of granulocyte-macrophage colony-stimulating factor (GM-CSF) were added to induce differentiation into immature dendritic cells. On day 7, the culture medium from SU-DHL-2/R cells treated with PBS, RTX, RTX + siPDK4, aCD20@Exo/siPDK4, and aCD20@Exo^CTX^/siPDK4 was collected, centrifuged at 2000 rpm for 5 min to remove debris, and the supernatant was used as lysate. A Transwell chamber migration system was used, where lysates from SU-DHL-2/R cells treated in various ways were added to the lower chamber, and 5 × 10^5 BMDCs were planted in the upper chamber [27]. After further co-culturing for 48 h, cells from the lower chamber were collected on day nine and resuspended in a staining buffer containing anti-CD11c, anti-CD80, and anti-CD86. Flow cytometry was used to detect the expression of CD80 and CD86 on DCs and assess DC maturation induced under different treatments.

### In vitro isolation and activation level measurement of T cells

Following previous reports, Murine primary T cells were purified from spleen and LN cell suspensions obtained from C57BL/6 using the Mouse T cell Isolation kit [28]. Flow cytometry analysis confirmed that the T cells isolated by this method were 90–95% CD3^+^ [29]; subsequently, the activated T cells were co-cultured for 48 h with PBS, RTX, RTX + siPDK4, aCD20@Exo/siPDK4, and aCD20@Exo^CTX^/siPDK4. The T lymphocytes were washed thrice with PBS and then stained at 4 °C with anti-CD4 and anti-CD8 antibodies for 30 min. After washing three times with cold PBS, flow cytometry detected cell fluorescence.

### Humanized mouse immune system reconstruction and resistant tumor model establishment

The establishment of humanized NSG mice was described in previous studies [30]. Briefly, newborn NOD-scid IL2rγnull (NSG) mice were irradiated and injected with human CD34^+^ hematopoietic stem cells intravenously. Eight weeks later, the proportion of human CD45^+^ cells was measured; a proportion greater than 10% indicated successful modeling and suitability for subsequent experiments. Then, 1 × 10^7 SU-DHL-2/R cells were injected subcutaneously into NSG mice to construct a resistant tumor model. The size of the tumor was measured using a caliper to record the length (L, mm) and width (W, mm) of the cancer. The tumor volume (V, mm^3) was calculated using the formula V = (LW^2)/2. The implementation of this study complied with the ethical requirements of the Ministry of Science and Technology of the People’s Republic of China (Ethical Approval Number: [2023]CJ0051). The experimental process followed the International Association of Veterinary Editors’ “Guidelines for Authors on Animal Ethics and Welfare” consensus and local and national regulations. All procedures on experimental animals were performed under anesthesia to minimize their pain, suffering, and death to the greatest extent possible.

### In vivo biodistribution analysis of aCD20@Exo^CTX^/siPDK4 nanoparticles

In in vivo imaging studies, Cy5-siRNA was synthesized to reduce the interference from autofluorescence. To investigate the tumor-targeting capability of the PHK26-aCD20@Exo^CTX^/siPDK4 complex, nude mice bearing subcutaneous tumors were injected intravenously with either free Cy5-siPDK4 or PHK26-aCD20@Exo^CTX^/siPDK4, with each mouse receiving an equivalent amount of 20 mg Cy5-siPDK4^31^. At 6-, 12-, and 24-hours post-injection, the mice were anesthetized with isoflurane, and the distribution of free Cy5-siPDK4 and PHK26-aCD20@Exo^CTX^/siPDK4 within the body was detected using the IVIS Spectrum in vivo imaging system (PerkinElmer). 24 h after injection, the DLBCL-resistant tumor-bearing mice were euthanized, and organs such as the tumor, heart, liver, spleen, lungs, and kidneys were extracted. The fluorescence intensity in the tumor site and each organ was detected ex vivo using the in vivo imaging system.

### The Anti-DLBCL resistant subcutaneous tumor activity of aCD20@Exo^CTX^/siPDK4 nanoparticles

When the tumor volume reached approximately 100 mm^3, this was designated as day 0 (D0). Mice were randomly divided into five groups (six mice per group) and received intravenous injections of 100 µL of PBS, RTX, RTX + siPDK4, aCD20@Exo/siPDK4, and aCD20@Exo^CTX^/siPDK4, respectively. Each mouse received a dose of rituximab at 12.5 mg/kg, equivalent to 225–275 µg/mouse, administered every other day (D0, D3, D6, D9) for 4 treatments. Tumor size and mouse weight were recorded every other day, along with the mice’s survival time. Mice were euthanized before death, and anticoagulated whole blood, tumors, and major organs (heart, liver, spleen, lungs, kidneys) were collected. The whole blood was anticoagulated with EDTA, and its blood cell levels were measured using an automatic blood cell analyzer. After centrifuging at 3000 rpm for 10 min, serum enzyme levels were detected using an automatic biochemistry analyzer. The major organs and tumors were fixed with 4% paraformaldehyde, embedded in paraffin, sectioned, and subjected to H&E staining, immunofluorescence staining, qPCR, and Western blot analysis.

### In vivo maturation of dendritic cells

To detect DC maturation in vivo, tumor-draining lymph nodes (LNs) were collected after euthanasia of mice given different treatments of PBS, RTX, RTX + siPDK4, aCD20@Exo/siPDK4, and aCD20@Exo^CTX^/siPDK4. A single-cell suspension was obtained by grinding lymph nodes and passed through the 70 μm cell strainer. The frequency of matured DC in the LNs was then investigated by flow cytometry after staining with anti-CD11c, anti-CD86, and anti-CD80 antibodies (gating on CD11c^+^ cells).

### In vivo T cell infiltration

To detect T cell infiltration in vivo, the tumors were harvested collected after euthanasia of mice given different treatments of PBS, RTX, RTX + siPDK4, aCD20@Exo/siPDK4, and aCD20@Exo^CTX^/siPDK4, cut into small pieces, and immersed in a solution of 1.0 mg/mL collagenase IV, hyaluronidase (100 U) and 0.2 mg/mL DNase I for 60 min at 37 °C. After that, a single-cell suspension was obtained by homogenizing tumor cells and passed through the 70 μm cell strainer. The cell suspension was then incubated with antibodies. The cell suspension was stained with anti-CD3, anti-CD4, and anti-CD8 and then detected by flow cytometry.

### Flow cytometric analysis of treg cells in tumor tissue

To detect T cell infiltration in vivo, the tumors were harvested collected after euthanasia of mice given different treatments of PBS, RTX, RTX + siPDK4, aCD20@Exo/siPDK4, and aCD20@Exo^CTX^/siPDK4, cut into small pieces, and immersed in a solution of 1.0 mg/mL collagenase IV, hyaluronidase (100 U) and 0.2 mg/mL DNase I for 60 min at 37 °C. After that, a single-cell suspension was obtained by homogenizing tumor cells and passed through the 70 μm cell strainer. Add 50 µl of anti-CD3 and anti-CD4 antibody solution and incubate for 1 h at 4 °C in the dark. Resuspend the cells in 500 µl of cell staining buffer, then centrifuge at 1200 rpm/min for 5 min and discard the supernatant. Add 100 µl of Foxp3 Fixation/permeabilization solution and incubate for 60 min at 4 °C in the dark. Wash the cells once with 1 ml of permeabilization buffer, resuspend in 100 µl of the buffer, add 50 µl of anti-Foxp3, and incubate for 20 min at room temperature in the dark. Wash once with 1 ml of buffer. Finally, resuspend the cells in 200 µl of buffer and transfer them to a flow cytometry tube for analysis.

### Statistical analysis

Statistical analyses were performed using SPSS 20.0 software, with continuous variables presented as mean ± SD. T-tests, analysis of variance (ANOVA), and non-parametric rank sum tests were used to explore differences between groups (*p < 0.05; **p < 0.01; ***p < 0.001 indicate statistically significant differences).

## Results

### PDK4 interacts with HDAC8 and influences the phosphorylation of HDAC8

It has been found that in DLBCL, PDK4 mediates resistance to rituximab by inhibiting CD20 expression [8], yet the specific mechanism by which PDK4 regulates CD20 in DLBCL requires further investigation. Previous research has primarily focused on the role of PDK4 in the cytoplasm and mitochondria, but recent studies have revealed that PDK4 also plays a significant role in the cell nucleus. [31]. Investigating the cellular localization of PDK4 in DLBCL could further elucidate the mechanism by which PDK4 regulates CD20 expression. Initially, tumor tissues from DLBCL patients sensitive and resistant to rituximab chemotherapy were collected, and the expression and cellular localization of PDK4 in resistant tumor tissues were explored through immunohistochemistry. It was found that in resistant tumor tissues, PDK4 localization includes both the cytoplasm and the nucleus **(**Fig. 1A**)**.

Using immunofluorescence and WB nuclear-cytoplasmic fractionation techniques, we found that PDK4 is predominantly localized in the nucleus of resistant cells (SU-DHL-2/R, OCI-LY8/R) compared to sensitive cells (SU-DHL-2, OCI-LY8) **(**Fig. 1B-C**)**. Therefore, the negative regulation of CD20 transcription, thereby promoting rituximab resistance, may be regulated by nuclear PDK4 protein.

To clarify the regulatory mechanism, lentiviral shRNA was used to knock down PDK4 in SU-DHL-2/R cells. Sh-PDK4 significantly reduced the mRNA and protein levels of PDK4 in SU-DHL-2/R and OCI-LY8-R cells (Supplementary Fig. 1A-B). Three groups of sh-PDK4 cells were randomly selected as the experimental group, with their corresponding three groups of sh-NC cells as the control group for RNA-seq sequencing analysis. Using |Fold change| > 2 and FDR < 0.05 as criteria for selection, 715 significantly upregulated genes were marked in red in the volcano plot, and 1145 downregulated considerably genes were marked in green (Supplementary Fig. 1C). KEGG enrichment analysis identified the top 20 biological processes different in drug-resistant cells, among which (Drug resistance: Antineoplastic) closely matches the research objectives of this project (Supplementary Fig. 1D). Drug sensitivity analysis suggests that high expression of PDK4 also upregulates the IC50 values of most drugs, leading to multidrug resistance (Supplementary Fig. 1E). Based on this biological function, the applicant screened for high and low expression genes (top 10, respectively) between the sh-PDK4 group and the sh-NC group for hierarchical clustering analysis, the results of which are displayed in the heatmap of Supplementary Fig. 1F. qRT-PCR was used to detect 20 significantly different genes in DLBCL drug-resistant cells (SU-DHL-2/R and OCI-LY8/R) between the sh-NC group and the sh-PDK4 group, showing that HDAC8 had the most significant difference in the two strains of drug-resistant cells in the sh-PDK4 group and its expression was significantly reduced (Supplementary Fig. 2A-B).

Western Blot analysis showed that the protein expression of HDAC8 in drug-resistant cells (SU-DHL-2/R and OCI-LY8/R) was significantly higher than that in sensitive cells (SU-DHL-2 and OCI-LY8) (Fig. 1D). Therefore, we believe that HDAC8 may play an important role in the process of PDK4-mediated rituximab resistance.To further clarify the interaction between HDAC8 and PDK4, we downloaded the three-dimensional protein structure files of HDAC8 and PDK4 from the Protein Data Bank. We performed three-dimensional and two-dimensional force analysis and visualization using AutoDockTools, ZDock, and PyMol software. In the three-dimensional images, HDAC8 is represented as a dark blue cartoon model, PDK4 as a cyan cartoon model, and their binding sites are shown as sticks of corresponding colors (Fig. 1E). In the two-dimensional images, several residues between HDAC8 and PDK4 form hydrogen bonds, such as the hydrogen bond formed between ASP183 of HDAC8 and LYS284 of PDK4. Under the influence of these interaction forces, the score of HDAC8-PDK4 is 1515.129 kcal/mol. Generally, the higher the binding energy between proteins, the more stable the binding. The above results suggest that HDAC8 may strongly interact with PDK4 (Fig. 1F). To further explore whether HDAC8 acts as a target of PDK4 in DLBCL, we first performed nuclear-cytoplasmic fractionation on sensitive cell lines and examined the binding of PDK4 and HDAC8 in both the nucleus and cytoplasm. The results showed that in sensitive cell lines, the expression level of PDK4 in the cytoplasm was significantly higher than in the nucleus. Moreover, the binding intensity of PDK4 and HDAC8 was significantly higher in the nucleus compared to the cytoplasm(Supplementary Fig. 2C-D). To further explore whether HDAC8 is a target of PDK4 in DLBCL, we observed a strong interaction between PDK4 and HDAC8 in SU-DHL-2/R cells through co-immunoprecipitation (Co-IP) experiments(Fig. 1G-H). In summary, this experiment indicates an interaction between PDK4 and HDAC8 proteins in DLBCL drug-resistant cells [32]; PDK4 is a protein kinase that can phosphorylate various target proteins. It has been found that the main phosphorylation site of HDAC8 is Ser-39 at the N-terminus. The applicant verified the protein expression by collecting proteins from the drug-resistant cell control group, PDK4 knockdown group, and knockdown control group using Western Blot. As shown in Fig. 1I-L, The expression of phosphorylated HDAC8 (p-HDAC8 Ser-39) protein was significantly downregulated in the PDK4 knockdown group, but there was no significant change in the expression of phosphorylated HDAC8 protein in the control group and the knockdown control group. In addition, there was no significant difference in the expression of HDAC8 protein between the groups.

### PDK4 promotes rituximab resistance by inhibiting CD20 expression through phosphorylation and activating HDAC8’s deacetylation function

**Fig. 2:**
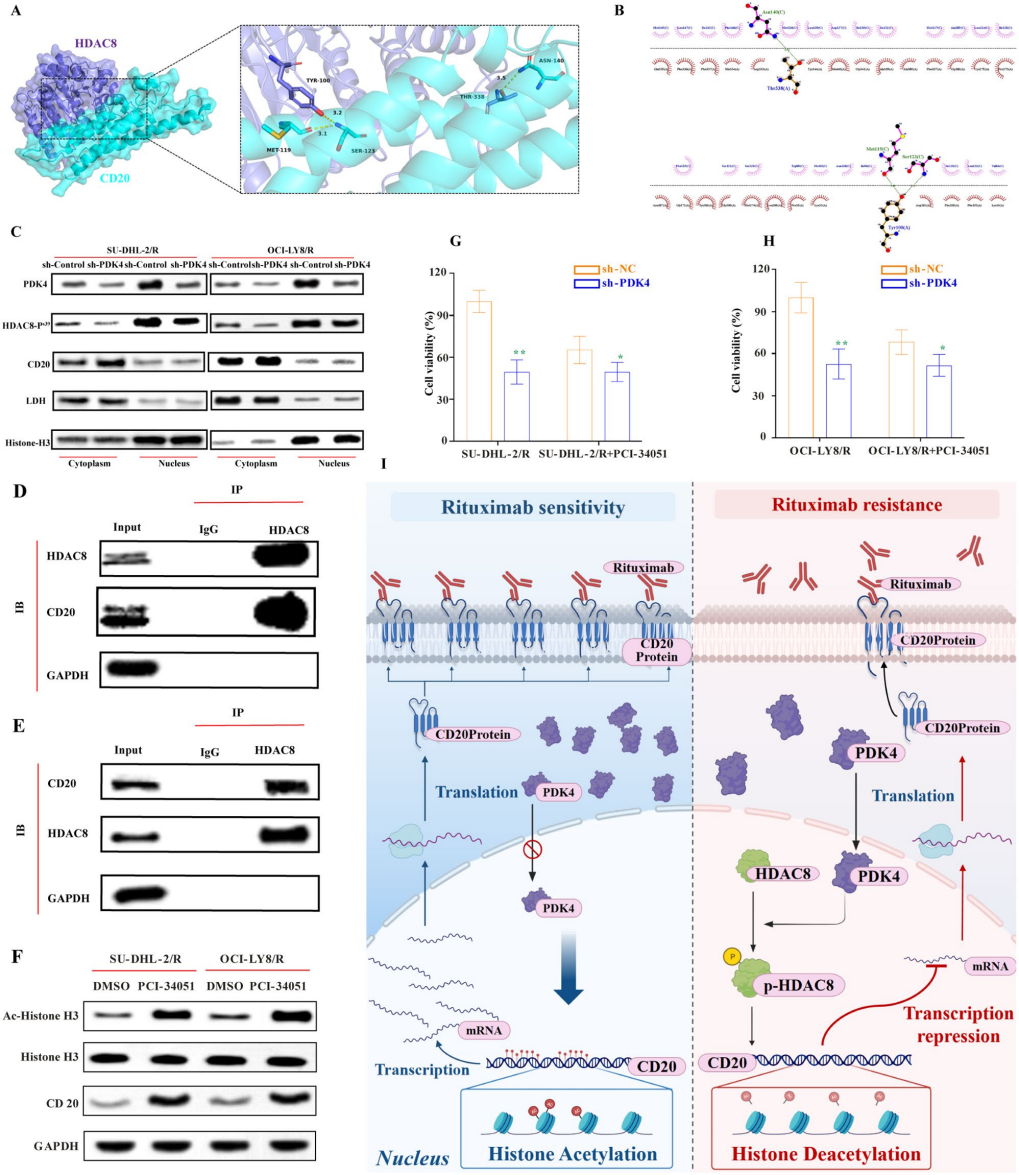
(**A**) Analysis of the protein-protein interaction interface between HDAC8 and CD20 based on structure. The image represents the predicted structure of the HDAC8-CD20 complex, where interactive hot spot residues are marked. (**B**) Two-dimensional angle analysis and visualization of the interaction forces between HDAC8 and CD20 proteins. The 100th Tyr amino acid residue of HDAC8 protein can bind through hydrogen bonds with the 119th Met and the 123rd Ser amino acid residues of the CD20 protein, with lengths of 2.48Å and 3.16Å, respectively; the 338th Thr amino acid residue of HDAC8 can bind through a hydrogen bond with the 140th Asn amino acid residue of CD20 protein, with a length of 3.54Å; in the two-dimensional graph, green dashed lines represent hydrogen bonds. (**C**) Nuclear-cytoplasmic separation, Western blot detects the expression and localization of PDK4, p-HDAC8, and CD20 after downregulation of PDK4, where LDH and Histone-H3 are used as cytoplasmic and nuclear internal references, respectively (**D**) In SU-DHL-2/R cells, HDAC8 was immunoprecipitated using an anti-HDAC8 antibody, then CD20 was detected by a western blotting assay. The control immunoprecipitation was conducted with IgG. (**E**) In SU-DHL-2/R cells, CD20 was immunoprecipitated using an anti-CD20 antibody, and then HDAC8 was detected by a western blotting assay. The control immunoprecipitation was conducted with IgG. (**F**) Western blot confirms that the HDAC8 specific inhibitor PCI-34,051 can inhibit the deacetylase activity of HDAC8 and upregulate the expression of CD20 protein; (**G**-**H**) CCK-8 assay verifies that downregulation of PDK4 reverses rituximab resistance, but the HDAC8 inhibitor PCI-34,051 does not further enhance the reversal of rituximab resistance by downregulating PDK4. **vs. sh-NC: *P* < 0.01; *vs. sh-NC: *P* < 0.05; (**I**) A model diagram of the mechanism by which PDK4 downregulates CD20 expression and leads to rituximab resistance by phosphorylating HDAC8, promoting the deacetylation of CD20

To further explore whether HDAC8 mediates PDK4’s negative regulation of CD20, we first conducted molecular docking studies between HDAC8 and CD20. In the three-dimensional image, HDAC8 is represented as a dark blue cartoon model, and CD20 is displayed as a cyan cartoon model, with their binding points shown as sticks in corresponding colors **(**Fig. 2A**)**. In the two-dimensional image, multiple residues between HDAC8 and CD20 form hydrogen bonds, such as the hydrogen bonds formed by TYR100 of HDAC8 and MET119 and SER123 of CD20. Under these interactions, the scoring of HDAC8-CD20 is 2200.938 kcal/mol. Generally, the higher the binding energy between proteins, the more stable the binding. The above results suggest that there may be a strong interaction between HDAC8 and CD20 **(**Fig. 2B**)**. After separating the cytoplasm and nuclei of DLBCL-resistant cells, it was found that the knockdown of PDK4 could significantly downregulate the expression levels of nuclear PDK4 and phosphorylated HDAC8 but upregulate the cytoplasmic expression of CD20 **(**Fig. 2C**)**, where Histone H3 was used as an internal reference for the nucleus. LDH was the internal reference for the cytoplasm. Our experimental results found that PDK4 could phosphorylate the Ser-39 site of nuclear HDAC8, but since HDAC8 is mainly localized in the nucleus, the leading promoter of HDAC8 phosphorylation is likely nuclear PDK4. Studies have shown that the HDAC family can regulate the transcription of CD20, but whether HDAC8 regulated by PDK4 phosphorylation can effectively regulate the expression of CD20 in DLBCL cells requires further investigation. To further explore whether HDAC8 can effectively regulate CD20, we observed a strong interaction between HDAC8 and CD20 in SU-DHL-2/R cells through co-immunoprecipitation (Co-IP) experiments **(**Fig. 2D-E**)**. In summary, this experiment demonstrates that in DLBCL-resistant cells, there is an interaction between HDAC8 and CD20 proteins. Based on the results, PDK4, during the process of resistance to rituximab in DLBCL, could transcriptionally suppress the expression of CD20 by phosphorylating HDAC8, and there is an interaction between HDAC8 and CD20. However, the regulatory role of HDAC8 on CD20 warrants further investigation. To this end, we treated the drug-resistant cell lines SU-DHL-2/R and OCI-LY8/R with different concentrations of PCI-34,051 to determine the optimal concentration. This concentration should effectively inhibit the deacetylase activity of HDAC8 without affecting its expression levels (Supplementary Fig. 2E-F). At the selected optimal concentration, PCI-34,051 was applied to the resistant cells SU-DHL-2/R and OCI-LY8/R for 24 h, with DMSO used as a control. After treatment, we collected cell proteins and assessed the protein expression levels of HDAC8, acetylated histone H3 (Ac-Histone H3), histone H3, and CD20 using Western Blot analysis. As shown in Fig. 2F, PCI-34,051 could effectively inhibit the deacetylase activity of HDAC8 and upregulate the expression of CD20 protein. The results prove that HDAC8 plays a role in suppressing CD20 expression through its deacetylase activity. To verify that the suppression of CD20 expression by HDAC8, thereby mediating resistance to rituximab, is regulated by PDK4, the resistant cells of both the PDK4 knockdown and control knockdown groups were treated with the HDAC8 specific inhibitor PCI-34,051 for 24 h. CCK-8 assay revealed that the downregulation of PDK4 reverses rituximab resistance, but the knockdown control group was relatively insensitive to the reversal of rituximab resistance. The HDAC8 inhibitor PCI-34,051 significantly improved the reversal of rituximab resistance in the knockdown control group. Still, the effect of PCI-34,051 on reversing rituximab resistance in the PDK4 knockdown group did not show a significant enhancement **(**Fig. 2G-H**)**. In conclusion, nuclear PDK4 activates HDAC8 by phosphorylating the Ser-39 site of HDAC8, thereby deacetylating and suppressing the expression of CD20 protein **(**Fig. 2I**)**. Therefore, downregulating PDK4 can effectively promote the expression of CD20 and enhance the effect of rituximab, offering new strategies and methods for the clinical treatment of DLBCL patients with negative or low expression of CD20.

### Preparation and characterization of aCD20@Exo^CTX^/siPDK4 nanoparticles

**Fig. 3:**
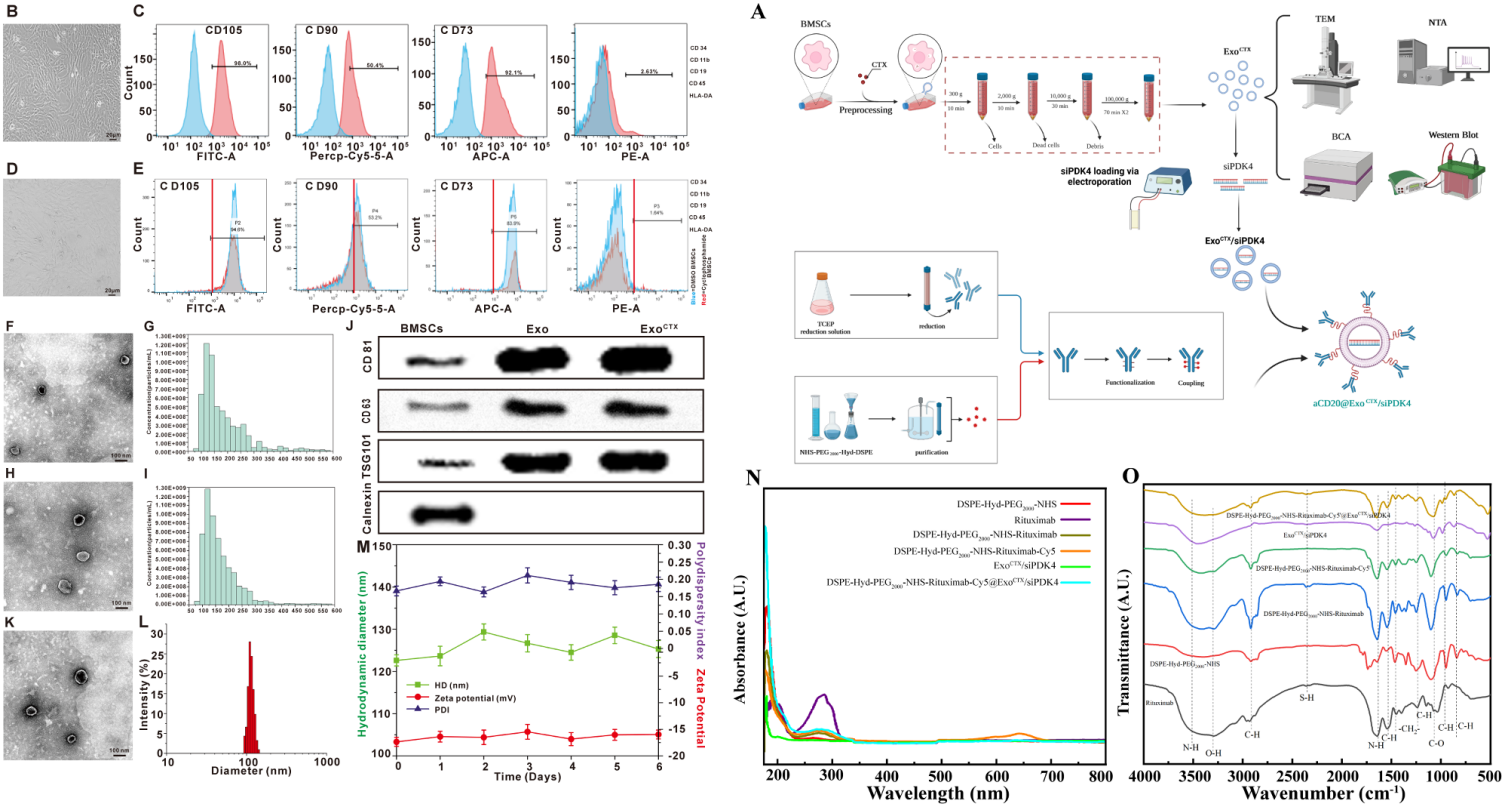
(**A**) Schematic diagram of the synthesis of aCD20@Exo^CTX^/siPDK4 nanoparticles. (**B**) Typical cellular morphology of BMSCs; (**C**) Flow cytometry analysis of BMSC lineage biomarkers, identifying specific surface markers that confirm the mesenchymal stem cell identity; (**D**) Typical cell morphology after cyclophosphamide (CTX) pretreatment of BMSCs; (**E**) Flow cytometry analysis of lineage biomarkers in CTX-pretreated BMSCs; (**F**) Morphology of mesenchymal stem cell-derived exosomes (Exo) observed under transmission electron microscopy (TEM), showing the typical vesicle-enclosed structures; scale bar: 100 nm. (**G**) Particle size range and concentration of Exo; (**H**) Morphology of exosomes extracted from CTX-pretreated BMSCs (Exo^CTX^) under TEM, maintaining the characteristic exosome appearance; scale bar: 100 nm; (**I**) Particle size range and concentration of Exo^CTX^; (**J**) Western blot analysis of surface markers on both types of exosomes; (**K**) TEM analysis of the morphology of the aCD20@Exo^CTX^/siPDK4 exosomal nanoparticle drug; (**L**) Particle size of aCD20@Exo^CTX^/siPDK4 exosomal nanoparticle drug; (**M**) Dynamic light scattering analysis of aCD20@Exo^CTX^/siPDK4 over six days, measuring hydrodynamic diameter (HD), zeta potential, and polydispersity index (PDI), providing insights into the stability and dispersion of the nanoparticles in solution. (**N**) UV–vis spectra of the Exo^CTX^/siPDK4, Rituximab, DSPE-Hyd-PEG_2000_-NHS, DSPE-Hyd-PEG_2000_-NHS-Rituximab, DSPE-Hyd-PEG_2000_-NHS-Rituximab-Cy5, andDSPE-Hyd-PEG_2000_-NHS-Rituximab-Cy5@Exo^CTX^/siPDK4(aCD20@Exo^CTX^/siPDK4).The inset shows the magnified UV–vis spectra from 200 to 800 nm. (**O**) Fourier-transform infrared spectroscopy (FTIR) analysis of the Exo^CTX^/siPDK4, Rituximab, DSPE-Hyd-PEG_2000_-NHS, DSPE-Hyd-PEG_2000_-NHS-Rituximab, DSPE-Hyd-PEG_2000_-NHS-Rituximab-Cy5, andDSPE-Hyd-PEG_2000_-NHS-Rituximab-Cy5@Exo^CTX^/siPDK4(aCD20@Exo^CTX^/siPDK4)

Based on the full bone marrow adherent method, bone marrow mesenchymal stem cells (BMSCs) are improved and isolated for culture. The cells adhere in a spindle shape, resembling fibroblasts, and increase in a radial and swirling pattern, arranged orderly **(**Fig. 3B**)**. Flow cytometry detects the lineage markers of the third-generation BMSCs: positive for CD73, CD90, and CD105, and negative for hematopoietic and endothelial markers (CD34, CD11b, CD19, CD45), and HLA-DR is negative **(**Fig. 3C**)**. To analyze the optimal working concentration of cyclophosphamide without affecting the vitality of bone marrow mesenchymal stem cells in our study, BMSCs were co-incubated with CTX at concentrations of 0 µmol/L, 75 µmol/L, 125 µmol/L, 250 µmol/L, 500 µmol/L, and 1000 µmol/L for 24 h, and cell vitality was assessed using the CCK-8 assay. The results (Supplementary Fig. 3) show that compared to the control group, a slight decrease in cell survival rate (> 90%) occurs when BMSCs are treated with 500 µmol/L cyclophosphamide, but a significant reduction in cell survival rate is observed when the concentration of cyclophosphamide reaches 1000 µmol/L. Additionally, pre-treatment of bone marrow mesenchymal stem cells with cyclophosphamide, followed by extraction of exosomes, does not affect the internalization rate of exosomes into SU-DHL-2/R cells (Supplementary Fig. 4). Therefore, in this study, exosomes were extracted after pretreating the bone marrow mesenchymal stem cells (BMSCs) with 500 µmol/L cyclophosphamide for 24 h in all experiments. Observation under an inverted fluorescence microscope revealed no apparent abnormal morphology in BMSCs after CTX pretreatment **(**Fig. 3D**)**, further indicating the low sensitivity of BMSCs to cyclophosphamide. Flow cytometry results showed that CTX does not affect the stemness characteristics of BMSCs **(**Fig. 3E**)**. After the successful isolation of exosomes, their morphology was identified and analyzed using transmission electron microscopy (TEM) **(**Fig. 3F and H**)** for exosomes secreted by normally cultured bone marrow mesenchymal stem cells (Exo) and those pretreated with cyclophosphamide (CTX) (Exo^CTX^). According to electron microscopy analysis, both types of exosomes were round or elliptical, vesicle-enclosed structures, with no significant morphological differences observed between the two groups. The team used the iZON qNano nanoparticle analysis system to measure the exosomes secreted by normally cultured BMSCs (Exo) and those pretreated with CTX (Exo^CTX^). The results **(**Fig. 3G and I**)** showed that the diameter of the exosomes primarily ranged from 80 to 170 nm, with an average diameter of about 120 nm. The concentration of exosomes in the samples was approximately 6.19 × 10^8 particles/ml, as analyzed by qNano. Western Blot was used to detect the markers of surface proteins on Exo and Exo^CTX^; the proteins CD81, CD63, and TSG101 were highly expressed in both groups of exosomes, and the protein Calnexin was almost absent **(**Fig. 3J**)**. Thus, it can be **see**n that the applicant successfully isolated and extracted the required exosomes secreted by BMSCs pretreated with CTX. Through WB and qPCR, the superior anti-PDK4 ability of SiPDK4 was validated (Supplementary Fig. 5A-B).The binding efficiency of exosomes and siRNA is a prerequisite for effective siRNA delivery. We used agarose gel electrophoresis to determine the loading rate of PDK4 siRNA in Exo, as shown in Supplementary Fig. 5C, indicating that the Exo^CTX^/siRNA nanoparticle complex was completely formed when the mass ratio of siRNA to exosomes was 4:1; under the conditions of a pulse time of 2 s, 400 V, and 125 µF, the loading rate of PDK4 siRNA in exosomes was approximately 31.71 ± 2.36%. The results suggest that exosomes have potential application value in delivering siRNA. To test the stability/anti-degradation capability of Exo^CTX^/siPDK4, we co-incubated Exo^CTX^/siPDK4 with RNase A (simulating the bodily fluid environment), with naked siPDK4 as the control group for 45 min. qRT-PCR results showed that the content of siPDK4 in Exo^CTX^/siPDK4 was significantly higher than that in naked siPDK4. After 45 min of treatment with RNase A, the Exo^CTX^/siPDK4 group retained 76.9 ± 9.48% of siPDK4 residues, while the naked siPDK4 group had 42.1 ± 6.32%. This indicates that exosomes, as natural carriers of siRNA, show good encapsulation ability and effectively prevent the degradation of siRNA in bodily fluids (Supplementary Fig. 5D). Further, we measured the transfection efficiency of Exo^CTX^/siPDK4 using flow cytometry. SU-DHL-2/R and OCI-LY8/R cells were cultured for 24 h, then incubated with Exo^CTX^/siPDK4 (100 nmol/L) for another 24 h before being collected by centrifugation at 1,000 g for 10 min. As shown in Supplementary Fig. 6, compared to the naked siPDK4 control group, 24 h after transfection, the transfection efficiency of Exo^CTX^/siPDK4 in SU-DHL-2/R and OCI-LY8/R cells was 82.1 ± 10.3% and 77.5 ± 8.6%, respectively, significantly higher than that of the naked siPDK4 group (*p* < 0.001).As shown in Supplementary Fig. 7, nuclear magnetic resonance (NMR), mass spectrometry results, and non-denaturing protein electrophoresis indicate that DSPE-Hyd-PEG2000-NHS and rituximab (RTX) were successfully coupled; the compound (named aCD20) formed by coupling RTX with NHS-Hyd-PEG2000-DSPE was then mixed with the exosome nanoparticles Exo^CTX^/siPDK4 in a 1:100 ratio in PBS, and gently rotated overnight at 4 °C. A novel exosomal nanoparticle drug, RTX-NHS-Hyd-PEG2000-DSPE @ExoCTX/siPDK4, was formed through dialysis and ultracentrifugation. The morphology of the exosomal nanoparticle drug aCD20@Exo^CTX^/siPDK4 shown in Fig. 3K-L did not exhibit significant differences from Exo and Exo^CTX^, with its particle size slightly increased to about 125 nm. Figure 3M study results indicate a zeta potential of -16 mV, suggesting that the negatively charged nanoparticles of aCD20@Exo^CTX^/siPDK4 can induce electrostatic repulsion, which ensures the physical stability of nanoparticles during storage and prevents the formation of aggregate [33]. Furthermore, nanoparticles carrying a negative charge often exhibit increased plasma circulation time [34, 35]. The average particle size of aCD20@Exo^CTX^/siPDK4 exosomal nanoparticle drug remained at 130 ± 5.0 nm with a polydispersity index (PDI) of 0.18 ± 0.03 and a zeta potential of -16 ± 1.2 mV over six days. Thus, the particle size, zeta potential, and dispersion index (PDI) of aCD20@Exo^CTX^/siPDK4 did not show significant changes, and additional research indicates that a PDI value below 0.2 signifies a uniform distribution and homogeneity of nanoparticles [36]. This further indicates the stability of aCD20@Exo^CTX^/siPDK4 over six days. Figure 3N depicted exosomes “re-educated” with cyclophosphamide (Exo^CTX^) did not show absorption bands in the near-infrared region. In contrast, Rituximab, DSPE-Hyd-PEG2000-NHS-Rituximab, DSPE-Hyd-PEG2000-NHS-Rituximab-Cy5 (as demonstrated in Supplementary Fig. 8, with an excitation wavelength of 666 nm and optimal excitation at 640 nm, where the fluorescence spectrum of Cy5 was detected, indicating successful modification of Cy5 on DSPE-Hyd-PEG2000-NHS-Rituximab), and DSPE-Hyd-PEG2000-NHS-Rituximab-Cy5@Exo^CTX^ displayed broad absorption bands in the near-infrared region. This difference in transverse and longitudinal resonance wavelengths between the DSPE-Hyd-PEG2000-NHS-Rituximab-Cy5@Exo^CTX^ exosome complex and unmodified Exo^CTX^ confirmed the interaction between Exo^CTX^ and DSPE-Hyd-PEG2000-NHS-Rituximab-Cy5. Furthermore, the UV-visible spectra of DSPE-Hyd-PEG2000-NHS-Rituximab, DSPE-Hyd-PEG2000-NHS-Rituximab-Cy5, and DSPE-Hyd-PEG2000-NHS-Rituximab-Cy5@Exo^CTX^ showed the characteristic absorption peak of Rituximab at 275 nm, while DSPE-Hyd-PEG2000-NHS did not exhibit a significant peak, indicating that DSPE-Hyd-PEG2000-NHS-Rituximab was successfully modified on Exo^CTX^. The Fourier-transform infrared spectroscopy (FTIR) revealed vibrations at 2930 cm − 1, 1532 cm − 1, 1230 cm − 1, 946 cm − 1, and 833 cm − 1 attributed to –CH– vibrations; 3520 cm − 1 and 1639 cm − 1 attributed to N–H vibrations; 3300 cm − 1 attributed to O–H vibrations; 2359 cm − 1 attributed to S–H vibrations; 1449 cm − 1 attributed to –CH2– vibrations; and 1069 cm − 1 attributed to C–O vibrations. Absorption bands in the functional group region above 1500 cm − 1 have a clear correspondence between functional groups and frequencies, with sparse and easily identifiable bands; the region below 1500 cm − 1, known as the fingerprint region, features a series of absorptions characteristic of the molecule generated by its overall weak vibrations **(**Fig. 3O**)**. These results further indicate that DSPE-Hyd-PEG2000-NHS-Rituximab was successfully modified on Exo^CTX^, and the newly synthesized exosomal nanoparticle complex is named aCD20@Exo^CTX^/siPDK4.

### The in vitro release characteristics, blood compatibility, and cellular uptake of aCD20@Exo^CTX^/siPDK4 nanoparticles

**Fig. 4:**
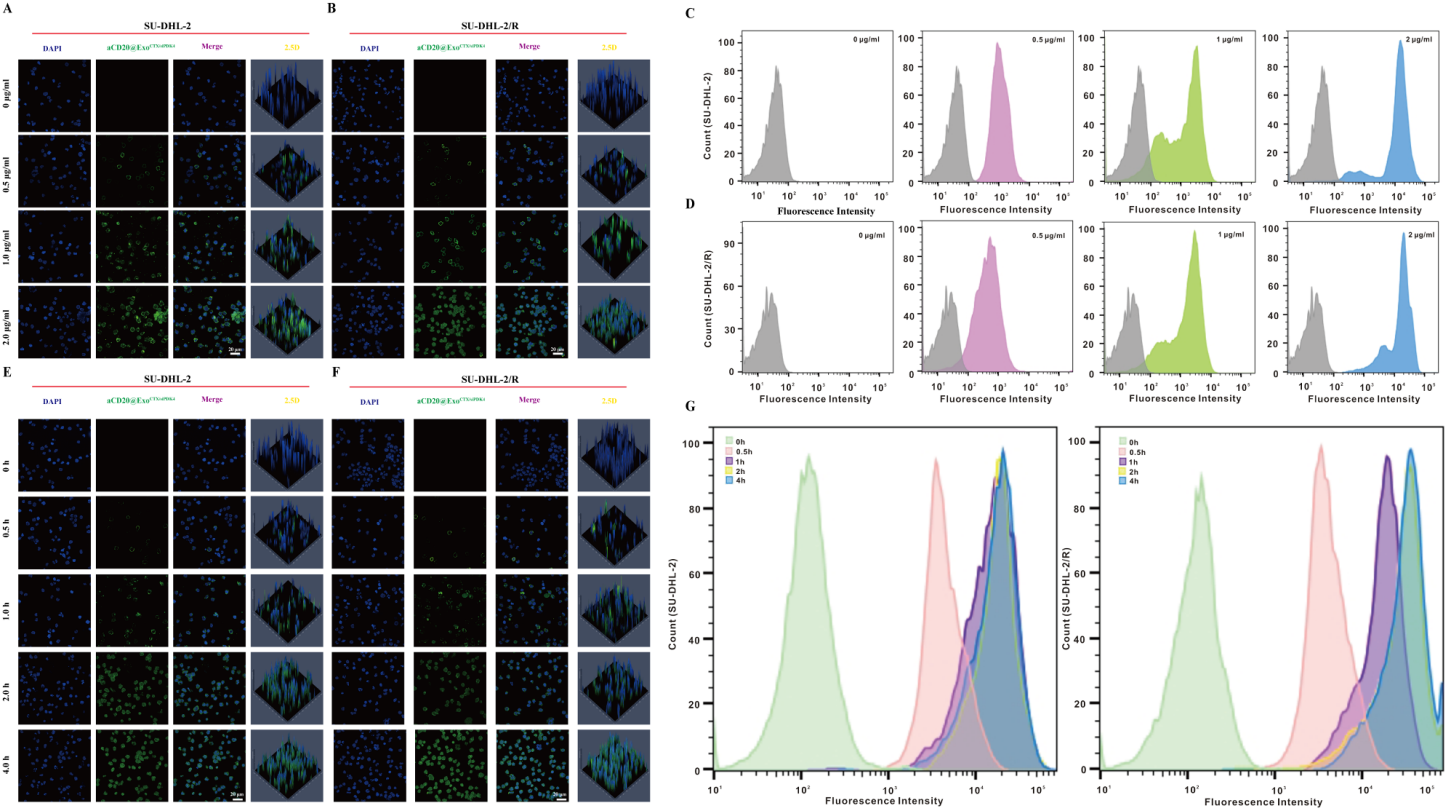
(**A**-**B**) CLSM images of SU-DHL-2 or SU-DHL-2/R cells after incubation with increasing PKH67-labeled aCD20@ExoCTX/siPDK4 concentrations. Scale bar: 20 μm. (**C**) FCM analysis of intercellular uptake of aCD20@Exo^CTX^/siPDK4 NPs in SU-DHL-2 and SU-DHL-2/R cells at various doses. (**E**-**F**) Fluorescence images and (**G**) FCM analysis of intercellular uptake of aCD20@Exo^CTX^/siPDK4 NPs in SU-DHL-2 and SU-DHL-2/R cells at different times. Scale bar: 20 μm

To investigate the in vitro release properties of the nanoparticle aCD20@Exo^CTX^/siPDK4, the nanoparticles were suspended in PBS at pH 7.4 (representing the pH conditions of blood or normal organs) or pH 6.5 (mimicking the extracellular microenvironment of tumor cells), and the dissociation process is shown in Supplementary Fig. 9A. The suspensions were incubated at 37 °C in a constant temperature shaker at 100 rpm, and supernatants were collected every 24 h. The BCA method detected the release of antibodies, and the release rate of nanoparticles under different pH conditions was calculated. The results showed (Supplementary Fig. 9B) that the release rate within 72 h was 21.2 ± 1.8% under neutral conditions at pH 7.4 and 90.3 ± 2.8% under acidic conditions at pH 6.5, indicating that nanoparticles could effectively release antibodies under acidic conditions to exert their functions. Given that the nanoparticles are intended for intravenous injection to function inside the body, various concentrations of nanoparticles were co-incubated with mouse erythrocytes at 37 °C. The absorbance of supernatants at 540 nm was measured with a microplate reader to calculate the hemolysis rate of erythrocytes. The experimental results demonstrated that nanoparticles at different concentrations exhibited good blood compatibility, with a hemolysis rate below 5% for all concentrations tested (Supplementary Fig. 10A). Subsequently, the protein adsorption capacity of the nanoparticles was tested, revealing that under both pH 6.5 and pH 7.4 conditions, all nanoparticles showed minimal protein adsorption, indicating good stability of the nanoparticles (Supplementary Fig. 10B). Cytotoxicity assays (Supplementary Fig. 10C) revealed that even at a high dosage (2 mg/mL), all formulations had virtually no effect on the viability of normal B cells. These results suggest that the nanoparticles possess good blood compatibility, stability, and safety, laying an experimental foundation for the in vivo application of the nanoparticles. DLBCL is classified into two main types: Germinal Center B-cell-like (GCB type) and Activated B-cell-like (ABC type). Compared to the GCB subtype, the ABC subtype exhibits more resistance to the existing standard treatment regimen based on rituximab [37]. Therefore, we selected the ABC subtype cell line SU-DHL-2 and the rituximab-resistant cell line SU-DHL-2/R for subsequent experiments. To explore the cellular uptake of nanoparticles, we first examined the relationship between nanoparticle cellular uptake and nanoparticle dosage. Different concentrations of aCD20@Exo^CTX^/siPDK4 (labeled with PKH67) were co-incubated with SU-DHL-2 or SU-DHL-2/R cells. One hour later, the fluorescence intensity of the exosome nanoparticle complexes entering the cells was observed under a laser confocal microscope. As the concentration of aCD20@Exo^CTX^/siPDK4 increased, the fluorescence intensity in the tumor cells gradually increased, reaching its maximum when the cells were co-cultured with 2 µg/ml of aCD20@Exo^CTX^/siPDK4 nanoparticles **(**Fig. 4A-B**)**, and the same trend was confirmed by flow cytometry analysis **(**Fig. 4C**)**. Subsequently, the same concentration of aCD20@Exo^CTX^/siPDK4 nanoparticles was co-incubated with SU-DHL-2 or SU-DHL-2/R cells for different periods, and the fluorescence intensity was observed under a laser confocal microscope. The results showed that cellular uptake of nanoparticles increased over time, with significant cellular uptake occurring as early as 0.5 h of co-incubation. After 1 h, cellular uptake reached a stable state with no significant changes **(**Fig. 4E-F**)**. The fluorescence intensity within the cells was measured by flow cytometry, which was consistent with the results shown by the confocal microscope images indicating the fluorescence signal intensity **(**Fig. 4G**).**

**Validation of the antitumor effect and mechanism of aCD20@Exo**^**CTX**^**/siPDK4 nanoparticles in reversing drug resistance in vitro**.

**Fig. 5:**
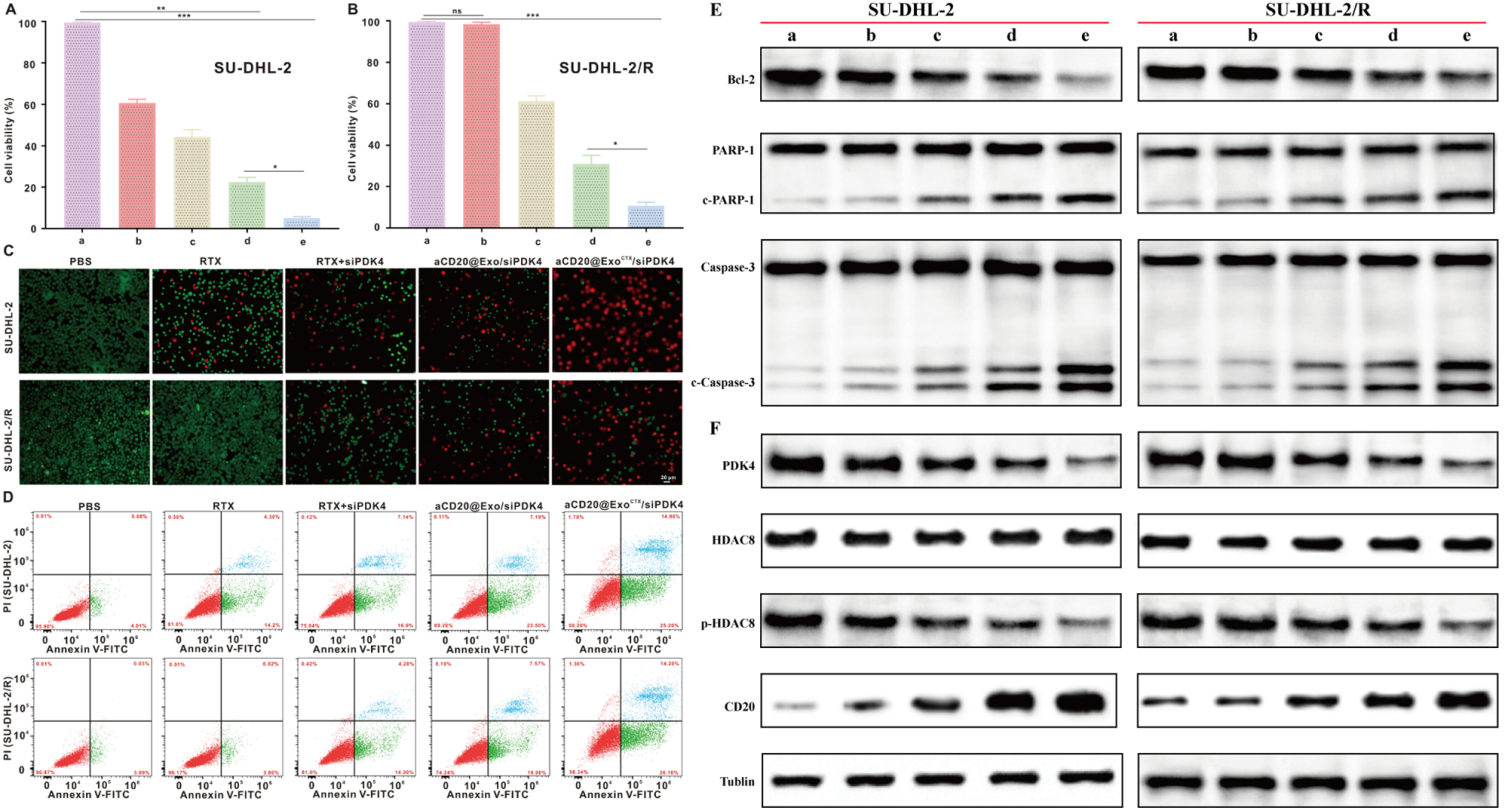
(**A**-**B**) After co-incubation for 24 h with PBS, RTX, RTX + siPDK4, aCD20@Exo/siPDK4, and aCD20@Exo^CTX^/siPDK4, the cytotoxicity towards SU-DHL-2 and SU-DHL-2/R cells was determined using a CCK-8 assay kit; (**C**) Fluorescent images of SU-DHL-2 and SU-DHL-2/R cells after 24 h co-incubation with PBS, RTX, RTX + siPDK4, aCD20@Exo/siPDK4, and aCD20@Exo^CTX^/siPDK4. Green: Calcein-AM; Red: PI. Scale bar: 20 μm; (**D**) The apoptosis rate of SU-DHL-2 and SU-DHL-2/R was detected by FCM. (**E**-**F**) The expression of Bcl-2, cleaved PARP, cleaved caspase-3, PDK4, HDAC8, p-HDAC8, and CD20 were assessed by western blot assay. Data are presented as means ± SD. *p < 0.05, **p < 0.01, ***p < 0.001. (Different groups are represented by a: PBS; b: RTX; c: RTX + siPDK4; d: aCD20@Exo/siPDK4; e: aCD20@Exo^CTX^/siPDK4)

To verify the in vitro reversal of drug resistance and antitumor effects of aCD20@ExoCTX/siPDK4 nanoparticles, we first examined their impact on the proliferation of SU-DHL-2 and SU-DHL-2/R cells using the CCK-8 assay and live/dead cell staining assay. The CCK-8 assay results showed that the proliferation of SU-DHL-2 and SU-DHL-2/R cells was significantly inhibited in the aCD20@ExoCTX/siPDK4 treatment group, with a marked statistical difference compared to other control groups (Fig. 5A-B). This demonstrates the specific cytotoxic effect of aCD20@ExoCTX/siPDK4 on drug-resistant SU-DHL-2/R cells. To visually confirm the cytotoxicity of aCD20@ExoCTX/siPDK4 nanoparticles, the study further employed the Calcein-AM/PI double staining method to detect cell viability. Following 24 h of co-incubation with PBS, RTX, RTX + siPDK4, aCD20@Exo/siPDK4, and aCD20@ExoCTX/siPDK4, fluorescence staining was performed to identify live cells (Calcein-AM, green) and dead cells (PI, red). As shown in Fig. 5C, the aCD20@ExoCTX/siPDK4 treatment group displayed clear red fluorescence in SU-DHL-2 and SU-DHL-2/R cells (with the largest area), indicating that cell viability was impaired by the nanoparticles, leading to a significant difference in cell viability compared to other groups. These experiments indicate that aCD20@ExoCTX/siPDK4 nanoparticles can effectively inhibit the proliferation of DLBCL cells. After 24 h of co-incubation of SU-DHL-2 and SU-DHL-2/R cells with different treatments, apoptosis was detected using flow cytometry. The results showed that the proportion of apoptotic cells was significantly increased in the aCD20@ExoCTX/siPDK4 treatment group compared to other control groups, indicating that early apoptosis in drug-resistant SU-DHL-2/R cells can be induced, further demonstrating the reversal of drug resistance (Fig. 5D and Supplementary Fig. 11A-B). Many biological events during the process of cell apoptosis are closely related to mitochondria, including the release of caspase activators and changes in the electron transport chain. Western blot results indicated that in SU-DHL-2 and SU-DHL-2/R cells treated with aCD20@Exo^CTX^/siPDK4 exosome nanoparticle complexes, the expression of Bcl-2 protein, which plays a vital role in the development of tumors, drug resistance, and apoptosis signaling, was significantly lower compared to other control groups. Additionally, clear cleavage bands of caspase-3 and PARP were observed **(**Fig. 5E and Supplementary Fig. 12A-B). These experimental results suggest that aCD20@Exo^CTX^/siPDK4 treatment can effectively induce apoptosis in DLBCL cells and reverse their drug resistance. Previous studies have shown that PDK4 activates HDAC8 by phosphorylating the Ser-39 site of HDAC8, leading to the deacetylation and suppression of CD20 expression. Therefore, after co-incubating SU-DHL-2 or SU-DHL-2/R cells with different treatments for 24 h, Western blot results also showed that in the drug-resistant cells treated with aCD20@Exo^CTX^/siPDK4 nanoparticles, the expression of PDK4 and phosphorylated HDAC8 was significantly inhibited, thereby further promoting the high expression of CD20 **(**Fig. 5F and Supplementary Fig. 12A-B). Hence, aCD20@Exo^CTX^/siPDK4 nanoparticles can effectively promote the expression of CD20 by inhibiting the PDK4/HDAC8/CD20 signaling axis and downregulating PDK4, thereby sensitizing the effect of rituximab. This provides a new strategy and theoretical basis for treating DLBCL patients resistant to therapy.

### Single-cell resolution overview of the DLBCL immune microenvironment and detection of aCD20@Exo^CTX^/siPDK4-induced in vitro ICD formation and immune cell activation

**Fig. 6:**
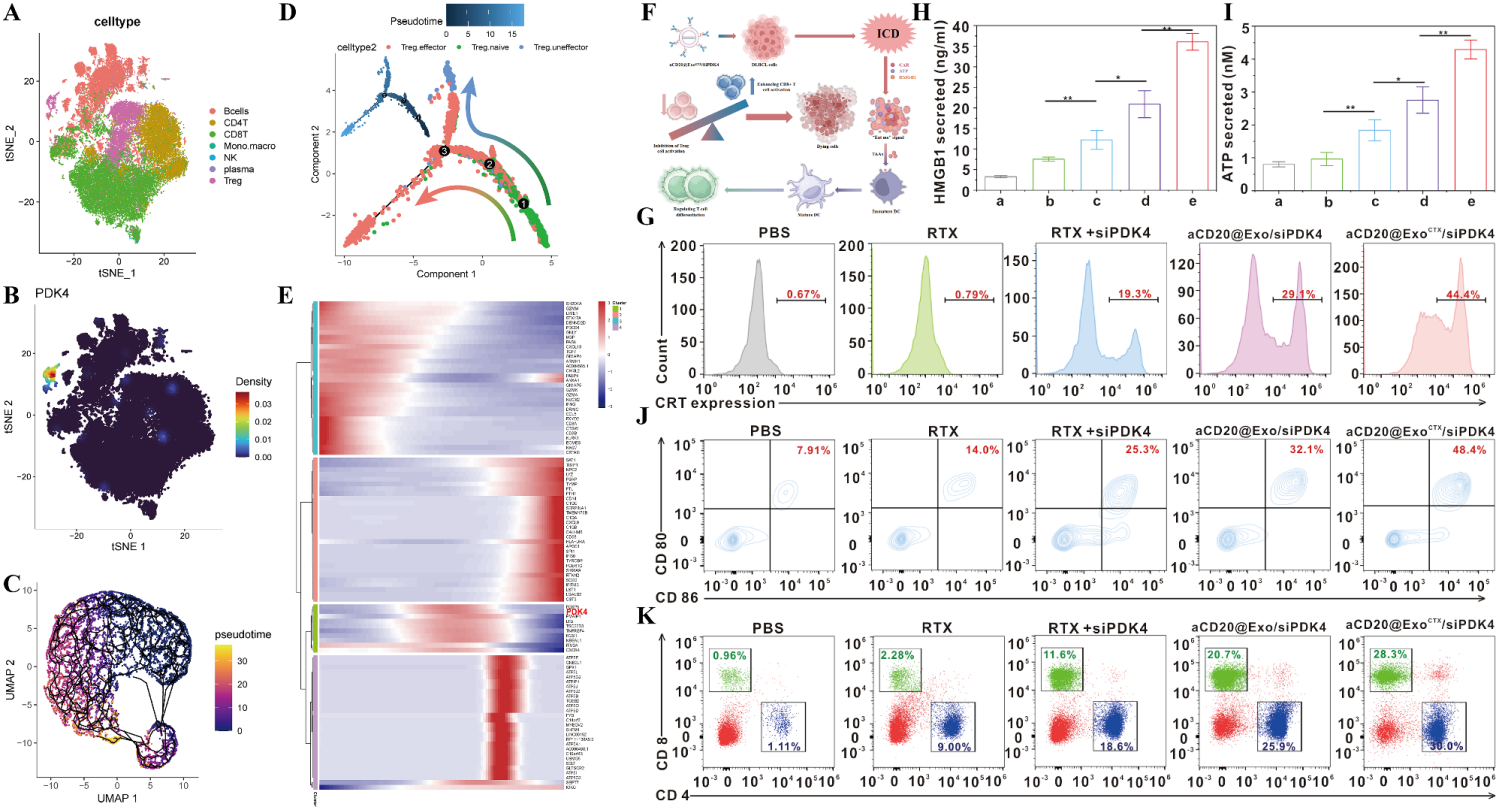
(**A**) t-SNE plot categorized by cell types; (**B**) t-SNE plot of PDK4 relative expression; (**C**) Monocle3 showing the differentiation time and pathways of Tregs; (**D**) Monocle2 showing the differentiation time and pathways of Tregs; (**E**) Heatmap of hierarchical clustering for developmental timing and subgroup-specific marker genes; (**F**) Schematic illustration of the exosome nanoparticle aCD20@Exo^CTX^/siPDK4 inducing immunogenic cell death (ICD) in tumor cells and activating anti-tumor immunity; (**G**) Flow cytometry analysis of Calreticulin (CRT) expression on the surface of SU-DHL-2/R cells; (**H**) ELISA detection of HMGB1 release from SU-DHL-2/R cells; (**I**) Enhanced ATP assay kit detection of ATP release from SU-DHL-2/R cells under different treatments; (**J**) DC maturation (CD80^+^CD86^+^ of CD11c^+^ DCs) after cocultured with SU-DHL-2/R cells with different treatments; (**K**) T cells extracted from mouse spleen treated with different formulations, stained with anti-CD4 and anti-CD8, flow cytometry detection of the ratio of CD4^+^ and CD8^+^ T cells. Data are shown as mean ± SD. (Different groups are represented by a: PBS; b: RTX; c: RTX + siPDK4; d: aCD20@Exo/siPDK4; e: aCD20@Exo^CTX^/siPDK4)

This study utilized publicly available single-cell transcriptome databases (GSE182434, heiDATA-VRJUNV, GSE252455, and GSE252608) to explore cell types and molecular characteristics in DLBCL. We first excluded cells with high mitochondrial gene expression, then applied highly variable genes (HVGs) and principal component analysis (PCA) for dimensionality reduction and clustering analysis while also examining the impact of cell cycle genes on cell classification (Supplementary Fig. 13A-C), and assessing the association between mitochondrial-related genes, cell counts (ncount), and features (nfeature) (Supplementary Fig. 13D-E). We further detailed each patient’s nfeature, ncount, and mitochondrial gene proportion (Supplementary Fig. 13F-H). After quality control and batch effect correction, 53,916 valid cells were obtained. Furthermore, we demonstrated the impact of different clustering resolutions on cell grouping through dendrogram analysis (Supplementary Fig. 13I). We showcased the uniform distribution of cells in two-dimensional space after batch effect removal through t-SNE (Supplementary Fig. 13J). By integrating and analyzing marker genes from published literature, we successfully identified seven significant immune cell populations, including B cells, CD8^+^ T, CD4^+^ T, Treg, Momo.macro, NK, and Plasma cells, and further analyzed the differentially expressed genes and their functions in the seven major cell types in DLBCL (Fig. 6A and Supplementary Fig. 14A-B). To further distinguish malignant B cells from normal B cells, we performed inferCNV analysis and successfully identified 12,689 malignant B cells (Supplementary Fig. 14C-D). We found that the level of CNV and the expression of PDK4 were significantly higher in malignant B cells compared to normal B cells (Supplementary Fig. 14E-F). To explore the distribution of PDK4 among tumor cell clusters, we further extracted malignant B cells for dimensionality reduction and clustering (Supplementary Fig. 14G). Interestingly, we found that the Malignant2 subgroup with high PDK4 expression was mainly enriched in the ABC subtype, which has a poorer prognosis, and exhibited the highest CNV levels. This further suggests the critical role of PDK4 in DLBCL (Supplementary Fig. 14H-L). We explored the relationship between the PDK4 gene and cell composition in the DLBCL-TME by combining bulk-seq and scRNA-seq analyses. We discovered that the TME of DLBCL is predominantly composed of B cells, showing significant heterogeneity in immune infiltration. This is consistent with previous studies [38–40]. Patients with high expression of CD8 + T cells present a better prognosis (Supplementary Fig. 15A-C).Interestingly, our data revealed that PDK4 is associated with most cell types in the TME(Supplementary Fig. 15D),especially highly expressed in Treg cells(Fig. 6B, Supplementary Fig. 14L),and patients with a high proportion of PDK4 + Treg cells have a poorer prognosis(Supplementary Fig. 15E-F). Cell communication results show that PDK4^+^ Tregs play a crucial role in comprehensive cell-to-cell communication, both as senders and receivers, with their communication strength and frequency significantly outperforming those of PDK4^−^ Tregs. The interaction between PDK4 + Tregs and B cells was also closer (Supplementary Fig. 16A-E). These findings suggest that PDK4 may significantly impact the biological functions of Tregs. To further understand the role of PDK4 in Tregs, we conducted a detailed subgrouping of Tregs, dividing them into Treg.naive, Treg.effector, and Treg.uneffector types found that PDK4 is mainly highly expressed in Treg.effector (Supplementary Fig. 17A-C). Based on the expression levels of PDK4, differential gene analysis was conducted on Tregs, followed by further KEGG, GO, GSVA, and GSEA analyses. We discovered that PDK4 may drive the formation of an immunosuppressive environment through multiple mechanisms, including promoting T cell differentiation and aberrant activation of B cell signaling pathways (Supplementary Fig. 17D-H). This finding suggests that PDK4 may play a vital role in the adverse prognosis of DLBCL by regulating the differentiation of Tregs. Our further pseudotime analysis depicted the differentiation process of Tregs from naïve to effector and then to uneffector (Fig. 6C-D), with the differentiation time heatmap highlighting the significant role of PDK4 in this process (Fig. 6E and Supplementary Fig. 18A-E).

Generally, tumor cells modulate immune cells to establish an immunosuppressive microenvironment [41]. However, under certain conditions, tumor cell death, such as immunogenic cell death (ICD), can release danger signals that enhance the immunogenicity of tumor cells, promote immune cell recognition of tumor cells, and activate anti-tumor immune responses [42]. Dendritic cells (DCs) are known to be one of the most potent antigen-presenting cells (APCs) in the immune system [43]. Chemotherapy has been shown to induce ICD, promoting DC maturation [44]. Mature DCs efficiently process antigens and then present them to T cells, promoting Their activation. CRT exposure, HMGB1 release, and ATP release are the three most essential signals in ICD [45]. This study will utilize the exosome nanomedicine aCD20@Exo^CTX^/siPDK4 to induce immunogenic death signals in DLBCL-resistant cells, promote immune cell recognition, and activate anti-tumor immunity (Fig. 6F). Next, the exposure of CRT in SU-DHL-2/R cells treated with PBS, RTX, RTX + siPDK4, aCD20@Exo/siPDK4, and aCD20@Exo^CTX^/siPDK4 will be detected using Alexa Fluor^®^ 488-labeled CRT antibody (green fluorescence) by flow cytometry. As shown in Fig. 6G and Supplementary Fig. 19A, the fluorescence intensity of cells treated with aCD20@Exo^CTX^/siPDK4 was significantly higher than that of the other groups, indicating that aCD20@Exo^CTX^/siPDK4 can lead to more CRT being transported to the cell surface. The results in Fig. 6H show no statistical difference between the RTX and the PBS groups (*P* > 0.05), indicating that RTX does not cause an increase in HMGB1 release from tumor cells. The concentration of HMGB1 in the aCD20@Exo/siPDK4 treatment group was 6.12 ± 0.76 times that of the PBS group, and the concentration of HMGB1 in the aCD20@Exo^CTX^/siPDK4 treatment group was 11.0 ± 1.39 times that of the PBS group, indicating that it can induce an increase in HMGB1 release from SU-DHL-2/R cells. Moreover, compared to the aCD20@Exo/siPDK4 group, the release of HMGB1 in the aCD20@Exo^CTX^/siPDK4 group significantly increased (*P* < 0.01). An enhanced ATP detection assay kit detected changes in ATP levels released from cells into the extracellular environment. The results in Fig. 6I show that the concentration of ATP in the aCD20@Exo/siPDK4 group was 2.76 ± 0.39 nmol/L, about 3.5 times that of the PBS group, and the concentration of ATP in the aCD20@Exo^CTX^/siPDK4 group was 4.29 ± 0.29 nmol/L, about 5.4 times that of the control group. The results above indicate that aCD20@Exo^CTX^/siPDK4 can cause a more significant release of ATP. The maturation of dendritic cells (DCs) marked by CD80 and CD86 was further evaluated through flow cytometry. The results showed that the PBS group and the RTX treatment group had almost no effect on DC maturation, while RTX + siPDK4, aCD20@Exo/siPDK4, and aCD20@Exo^CTX^/siPDK4 treatments induced DC maturation by 30.5%, 38.3%, and 56.0%, respectively, consistent with the ICD detection results **(**Fig. 6J and Supplementary Fig. 19B). Subsequently, the impact of aCD20@Exo^CTX^/siPDK4 on the maturation state of T cells in vitro was assessed through an in vitro T cell activation assay. Flow cytometry analysis showed that, compared to the control group PBS and other experimental groups RTX, RTX + siPDK4, aCD20@Exo/siPDK4, the proportion of CD8^+^ T cells was highest in the aCD20@Exo^CTX^/siPDK4 treatment group **(**Fig. 6K and Supplementary Fig. 19C). These results suggest that the exosome nanoparticle aCD20@Exo^CTX^/siPDK4 has advantages in promoting DC maturation and can further effectively induce antigen-specific T-cell responses.

### Construction of a subcutaneous tumor model in mice and in vivo validation of the anti-tumor efficacy of aCD20@Exo^CTX^/siPDK4 in reversing drug resistance

**Fig. 7:**
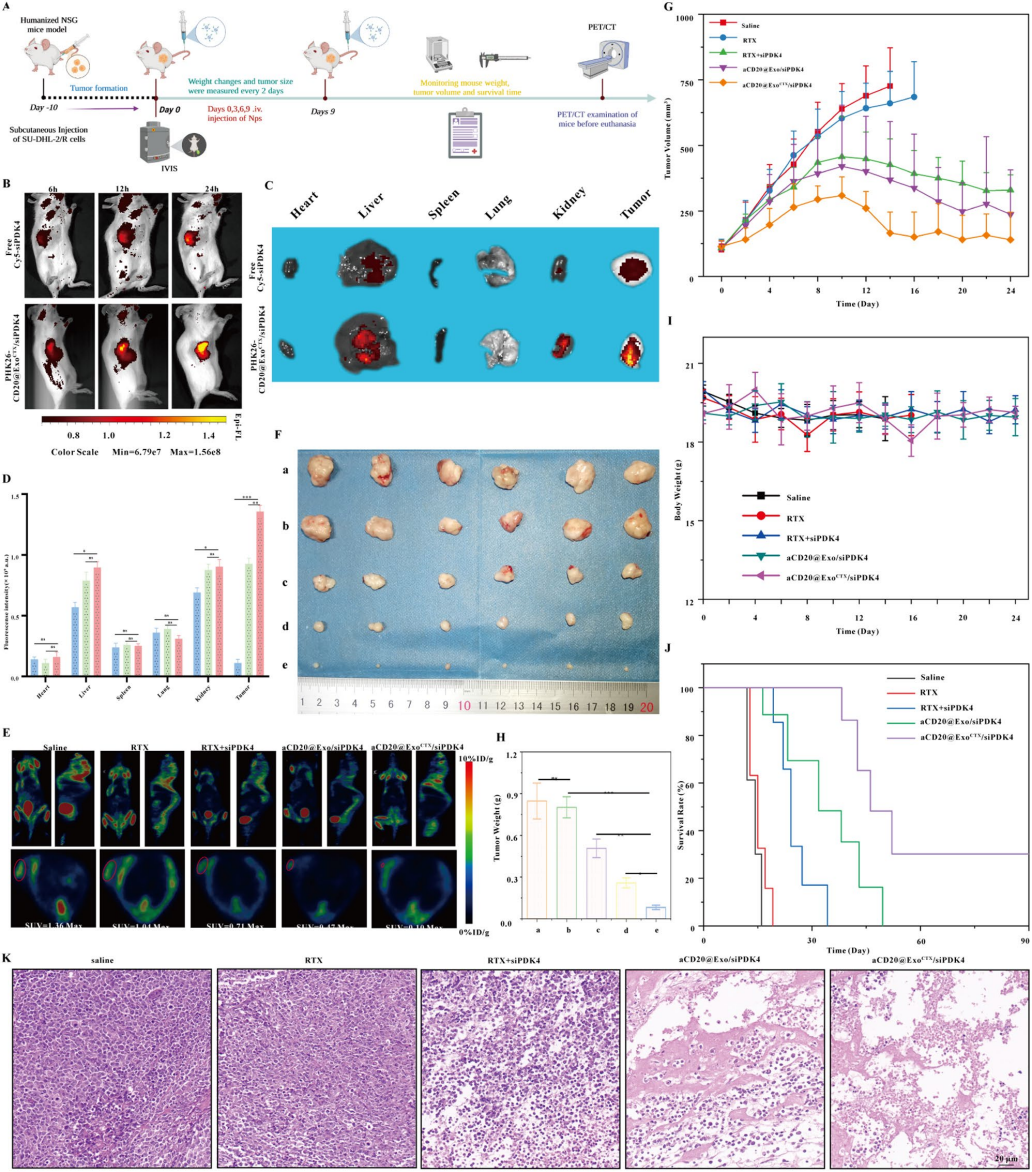
(**A**) Experimental scheme of aCD20@Exo^CTX^/siPDK4 reverses drug resistance and antitumor. (**B**) Fluorescent images were monitored in vivo at 6, 12, and 24 h following tail vein administration of Saline, Cy5-siPDK4, and PKH26-aCD20@Exo^CTX^/siPDK4 to the mice. (**C**) Fluorescence images of the main organs (heart, liver, spleen, lung, and kidney) and tumors after treatment with Saline、Cy5-siPDK4 and PKH26-aCD20@Exo^CTX^/siPDK4 for 24 h. (**D**) Semiquantitative assessment of fluorescence signal in main organs and tumors at 24 h. Data are presented as the means ± SD (*n* = 3) (intergroup comparisons: *p < 0.05, **p < 0.01, ***p < 0.001, ns, no significant difference.); (**E**) Representative PET/CT images for each group, obtained 24 h after mice were intravenously injected with ^18F-FDG (including coronal, lateral, and axial views). The red arrows indicate the liver tumor regions. Standard uptake values (SUV) for ^18F-FDG detection are presented. (**F**) Representative images of tumors following different treatment regimens; The tumor growth curves (**G**), Tumor weights (**H**), Body weights (**I**), and survival rates (**J**) of subcutaneousSU-DHL-2/R-tumor-bearing mice received various treatments as indicated (*n* = 6); (**K**) H&E staining of tumor slices collected from mice of different groups after treatments. Scale bar: 100 μm. (other groups are represented as follows: a: PBS; b: RTX; c: RTX + siPDK4; d: aCD20@Exo/siPDK4; e: aCD20@Exo^CTX^/siPDK4.)

**Fig. 8:**
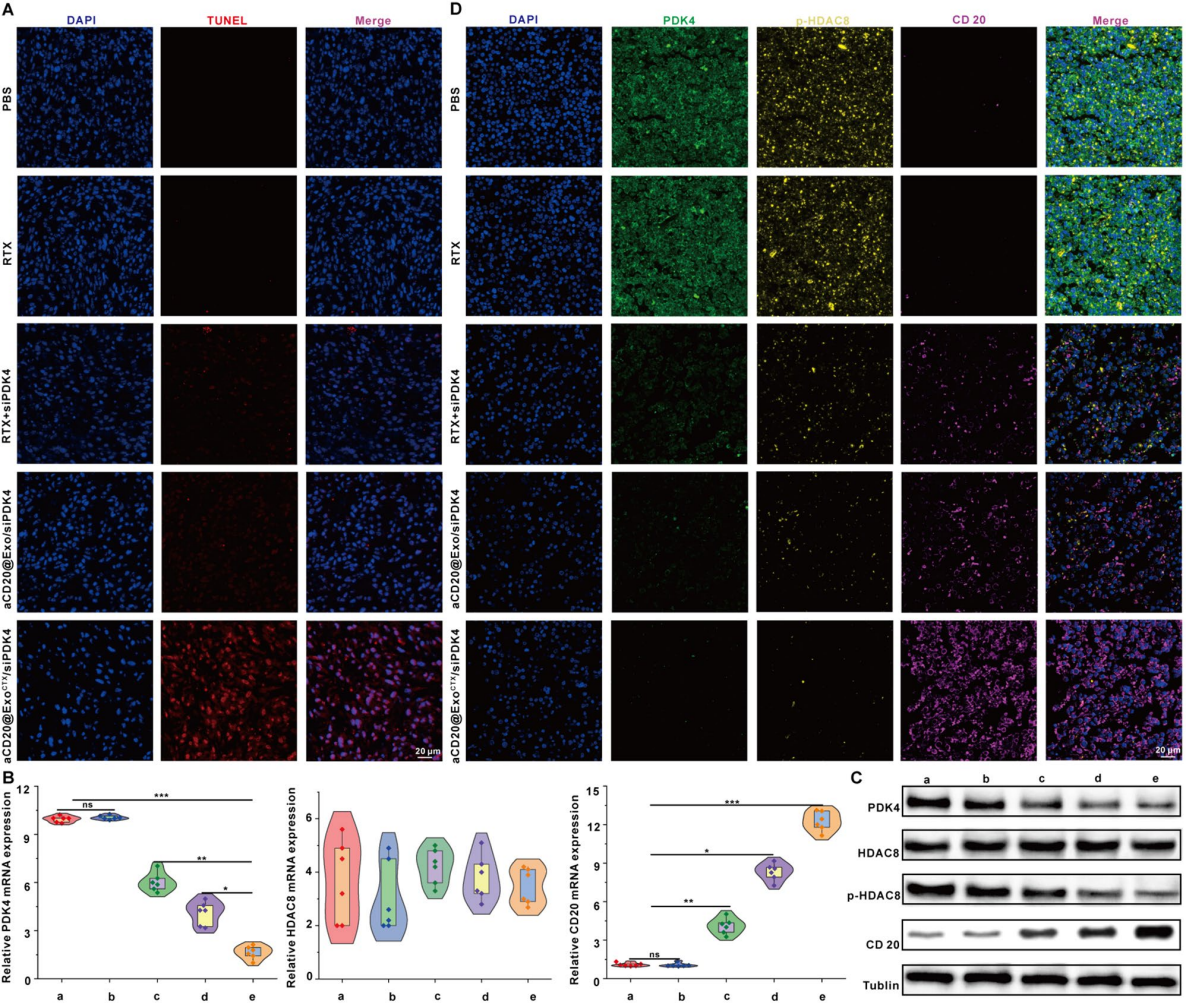
(**A**) TUNEL staining (red) of tumor tissues by immunofluorescence after injection with various treatments. Scale bar: 20 μm. (**B**) Expression levels of PDK4 mRNA, HDAC8 mRNA, and CD20 mRNA were detected by qRT-PCR in tumor tissues of mice after PBS(a), RTX(b), RTX + siPDK4(c), aCD20@Exo/siPDK4(d) and aCD20@Exo^CTX^/siPDK4(e), respectively. (**C**) The expression of PDK4, HDAC8, p-HDAC8 and CD20 in tumor tissues was detected by western blotting assay after PBS(a), RTX(b), RTX + siPDK4(c), aCD20@Exo/siPDK4(d) and aCD20@Exo^CTX^/siPDK4(e), respectively. (**D**) The animals were under the treatment of PBS、RTX、RTX + siPDK4、aCD20@Exo/siPDK4 and aCD20@Exo^CTX^/siPDK4, respectively, and PDK4 (green), p-HDAC8 (yellow) and CD20 (deep purple) in the tumor tissues were analyzed by immunofluorescence staining. Scale bar: 20 μm

To further confirm the reversal of drug-resistant antitumor effects of aCD20@Exo^CTX^/siPDK4 on subcutaneous tumors in mice, experiments were conducted after the successful construction of the SU-DHL-2/R subcutaneous drug-resistant tumor model **(**Fig. 7A**)**. Free Cy5-siPDK4 and PKH26-aCD20@Exo^CTX^/siPDK4 (40 µg siRNA equivalent, 150µL siRNA of 20 µM stock) were injected into mice via tail vein to explore the in vivo biological distribution of aCD20@Exo^CTX^/siPDK4^31^. 6–24 h post-injection, the fluorescence imaging of PKH26-aCD20@Exo^CTX^/siPDK4 was higher than that of the free siRNA, indicating that aCD20@Exo^CTX^/siPDK4 effectively accumulated in the tumor tissue **(**Fig. 7B**)**. This is likely related to the exosome’s ability to homing back to the tumor site [12]. 24 h after injection, the main organs and tumor tissues were extracted for ex vivo imaging. It was observed that the fluorescence intensity at the tumor sites of mice treated with aCD20@Exo^CTX^/siPDK4 exosome nanomedicine was higher than that in mice treated with free siRNA **(**Fig. 7C**)** and that aCD20@Exo^CTX^/siPDK4 was primarily cleared by the liver and kidneys **(**Fig. 7D**)**. This is consistent with previous reports that the liver can rapidly uptake, degrade, and eliminate nanomaterials [46], and the kidneys are an excretory organ where nanomaterials can be excreted into the urine [47]. Humanized mice were generated by myeloablation and transplantation of hCD34^+^ HSCs^30^, and the detailed construction process is shown in (Supplementary Fig. 20). The drug-resistant tumor model mice were randomly divided into five groups (each group *n* = 6): the Saline group, the RTX group, the RTX + siPDK4 group, the aCD20@Exo/siPDK4 group, and the aCD20@Exo^CTX^/siPDK4 group. The size and weight of the mice’s tumors were measured every other day. Further, ^18F-FDG was used as a metabolic marker for mouse tumors for PET/CT scanning. The PET/CT scan process, as shown in Fig. 7E, clearly indicated reduced ^18F-FDG accumulation and lower SUV values in the subcutaneous tumor regions marked with arrows in the aCD20@Exo^CTX^/siPDK4 treatment group compared to other groups, demonstrating significant antitumor effects of aCD20@Exo^CTX^/siPDK4 exosome nanomedicine on the subcutaneous drug-resistant tumor model. The tumor growth inhibition effect of the aCD20@Exo^CTX^/siPDK4 group was the most significant compared to the other groups, as shown in Fig. 7F. Additionally, the tumor volume and weight were the smallest in the aCD20@Exo^CTX^/siPDK4 group among all four groups **(**Fig. 7G-H**)**, with a tumor suppression efficiency of about 70.3%±6.69 compared to the PBS control group. Throughout the experiment, the weight of the mice in all groups was not affected by any treatment measures, indicating that aCD20@Exo^CTX^/siPDK4 has no apparent toxicity **(**Fig. 7I**)**. Moreover, compared to other experimental groups, the survival rate of the mice treated with aCD20@Exo^CTX^/siPDK4 was the highest **(**Fig. 7J**)**. Subsequently, H&E staining was used to analyze the tumor tissue morphology of each group, and the results from Fig. 7K suggest a reduction in tumor cell numbers, nuclear condensation, and a potential presence of a significant amount of apoptotic cells in the aCD20@Exo^CTX^/siPDK4 group. Furthermore, the apoptosis in tumor tissues was examined using the TUNEL assay. Compared to other groups, most tumor cells in the aCD20@Exo^CTX^/siPDK4 treated group showed red fluorescence, indicating that aCD20@Exo^CTX^/siPDK4 treatment promotes tumor cell apoptosis **(**Fig. 8A**)**.

### aCD20@Exo^CTX^/siPDK4 for in vivo reversal of drug resistance and anti-tumor mechanism validation

Subsequently, we investigated the molecular mechanisms of aCD20@Exo^CTX^/siPDK4 in reversing drug resistance in vivo. After treating mice with PBS, RTX, RTX + siPDK4, aCD20@Exo/siPDK4, and aCD20@Exo^CTX^/siPDK4, we employed immunofluorescence, qRT-PCR and Western blotting to determine whether aCD20@Exo^CTX^/siPDK4 exerts its drug resistance reversal effects via targeting the PDK4/HDAC8/CD20 signaling pathway. Further validation of the resistance reversal mechanism of aCD20@Exo^CTX^/siPDK4 at the transcriptional and translational levels was conducted. As shown in Fig. 8B, qRT-PCR analysis indicated that aCD20@Exo^CTX^/siPDK4 could suppress PDK4 mRNA expression while enhancing CD20 mRNA expression. Similar results were confirmed in Fig. 8C, where Western blotting results showed a significant decrease in PDK4 and p-HDAC8 protein expression in the tumor tissues of mice treated with aCD20@Exo^CTX^/siPDK4, while CD20 protein expression was significantly higher compared to other groups. Additionally, the fluorescence intensity of PDK4 (green), p-HDAC8 (yellow), and CD20 (deep purple fluorescence) in the mouse tumor tissues was detected using immunofluorescence. As shown in Fig. 8D, compared to other groups, the aCD20@Exo^CTX^/siPDK4 group significantly reduced the green and yellow fluorescence intensities of PDK4 and p-HDAC8 in mouse tumor tissues, indicating that the expression of PDK4 and p-HDAC8 was inhibited in vivo. Additionally, this group enhanced the fluorescence intensities of CD20 (deep purple) and Caspase-3 (red) in the mouse tumor tissues while suppressing the green fluorescence intensity of Bcl-2 (Supplementary Fig. 21). These results demonstrate that the aCD20@Exo^CTX^/siPDK4 exosome nanoparticle complex effectively inhibits PDK4 and, through dephosphorylation of nuclear HDAC8, upregulates CD20 expression, thereby reversing the molecular mechanism of rituximab resistance in DLBCL.

### aCD20@Exo^CTX^/siPDK4 induces antigen exposure and activates antitumor immune responses in vivo

**Fig. 9:**
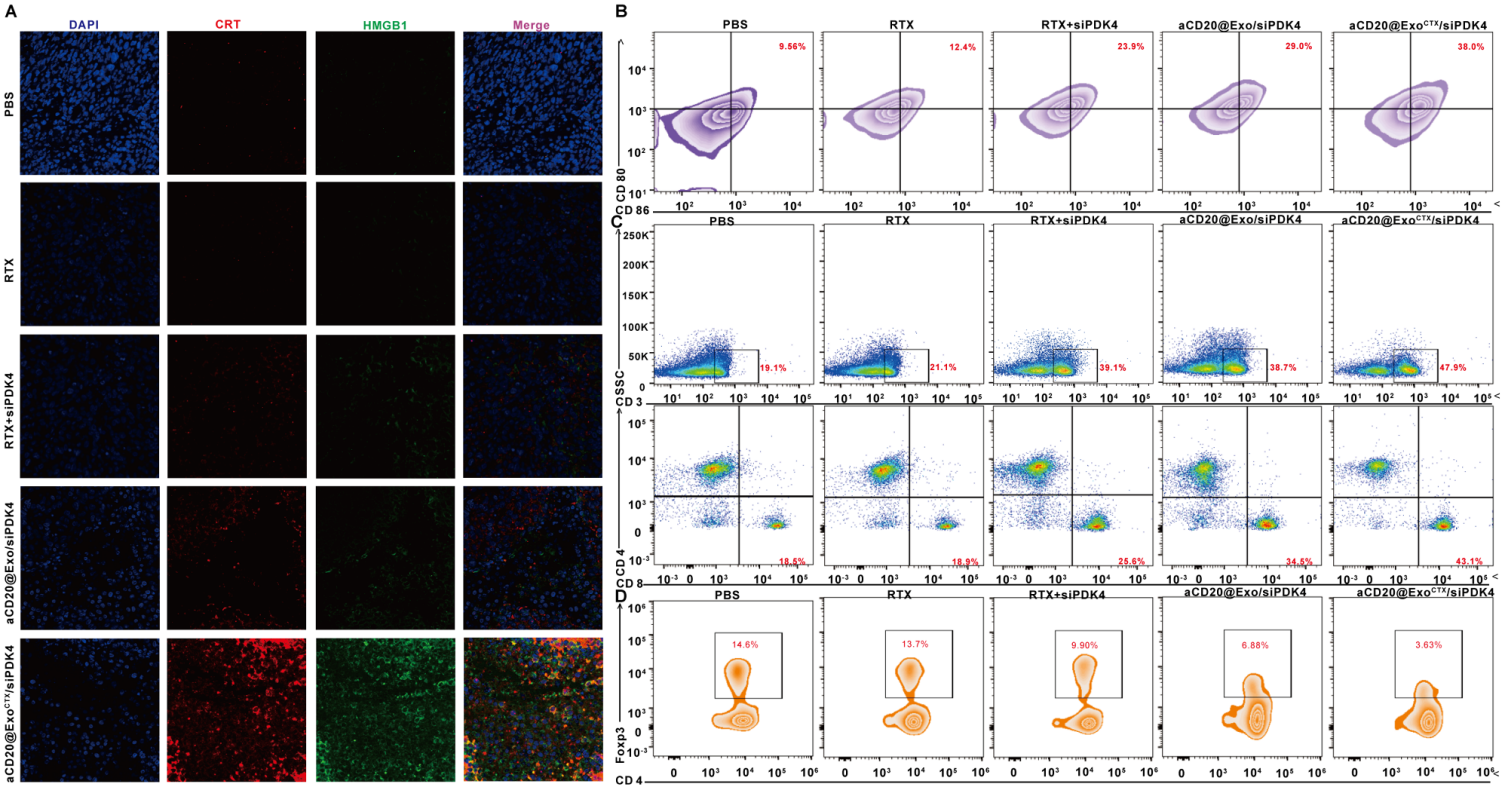
Antigen exposure and antitumor immune response induction in vivo. (**A**) Immunofluorescence staining of intratumoral CRT/HMGB1. Scale bar 20 μm. (**B**) DC maturation in the lymph nodes was detected by flow cytometry. (**C**) Proportion of tumor tissues CD3^+^ T cells on the day of euthanasia in mice, and Proportion of CD8^+^ T cells and CD4^+^ T cells (gated on CD3^+^T cells) on the day of euthanasia in mice. (**D**) The Treg cells (CD4^+^Foxp3^+^) were analyzed by flow cytometry

Immunofluorescence was used to observe the expression of ICD-related molecules in tumor cells. As shown in Fig. 9A, nearly all intratumoral cells in the aCD20@Exo^CTX^/siPDK4 group exhibited high fluorescence signals for CRT (red fluorescence) and HMGB1 (green fluorescence), which were completely different from the low fluorescence signals of CRT and HMGB1 displayed by other groups. The results indicate that aCD20@Exo^CTX^/siPDK4 can significantly enhance tumor ICD, thereby promoting the maturation of dendritic cells (DCs) and the activation of T cells, laying the foundation for triggering an effective immune response. Subsequently, flow cytometry was used to assess the maturation of lymph node DCs. As shown in Fig. 9B and Supplementary Fig. 22A, the proportion of CD80^+^CD86^+^ DCs in the aCD20@Exo^CTX^/siPDK4 group reached (37.21 ± 3.69%), significantly higher than the other groups due to the enhanced ICD, thereby inducing more robust DC maturation. Tumor tissues were then collected for T cell analysis, as shown in Fig. 9C and Supplementary Fig. 22B-C, the proportion of CD3^+^T and CD8^+^T cells in the aCD20@Exo^CTX^/siPDK4 group (42.78 ± 2.16%) was significantly higher than the other four groups. BMSC-derived exosomes still possess the immunosuppressive characteristics similar to their parent cells, inhibiting T cell activation and DC maturation and promoting the proliferation and differentiation of regulatory T cells (Tregs) (Supplementary Fig. 23). [17]. Further, using flow cytometry, the proportions of Tregs (CD4^+^CD25^+^Foxp3^+^) cells in the peripheral blood of healthy volunteers, newly diagnosed DLBCL patients, rituximab chemotherapy-sensitive patients, and rituximab-resistant patients were measured. The results (Supplementary Fig. 24A-E) indicated that the proportion of Tregs in rituximab-resistant DLBCL patients was significantly higher compared to the other three groups. This confirms that regulatory T cells (Tregs) are often present at high levels in cancer patients, and their function is to suppress immune responses [48]. Therefore, we employed cyclophosphamide to “re-educate” bone marrow mesenchymal stem cells and then constructed the exosome nanoparticle complex aCD20@Exo^CTX^/siPDK4. This approach was used to assess the cells collected from tumor tissues and measure CD4 and Foxp3 expression using flow cytometry, where the aCD20@Exo^CTX^/siPDK4 group significantly inhibited the activity of Treg cells in tumor tissues compared to other groups **(**Fig. 9D and Supplementary Fig. 22D). Additionally, serum cytokines, including IL-12, IFN-γ, and TNF-α, play a crucial role in cellular anticancer immunity. ELISA analysis showed that the levels of these cytokines were significantly elevated in mice treated with aCD20@Exo^CTX^/siPDK4 (Supplementary Fig. 25A-C). Similar to those of mature DCs and CD8 + T cells analysis, these results indicate establishing an antitumor immune response. In summary, these findings demonstrate that the “smart” exosome targeting delivery strategy of aCD20@Exo^CTX^/siPDK4 not only kills tumors but also enhances the tumor immune microenvironment by promoting immunogenic cell death, maturing DCs, activating CD8^+^ T cells, and suppressing Treg cells. This results in increased expression of serum cytokines, thereby triggering a systemic antitumor immune response and synergistically reversing tumor resistance to rituximab.

### Biocompatibility of aCD20@Exo^CTX^/siPDK4 in mice

The biocompatibility of nanomaterials is increasingly gaining attention in addressing medical and biological issues46. This section verifies the in vivo biosafety of aCD20@Exo^CTX^/siPDK4. By utilizing H&E staining, the histological changes and inflammatory cell infiltration in the major organs of mice (heart, liver, spleen, lungs, and kidneys) were examined to assess whether aCD20@Exo^CTX^/siPDK4 causes organ damage. The hematological and hepato-renal toxicity of aCD20@Exo^CTX^/siPDK4 in mice was evaluated by analyzing routine blood counts and biochemical contents in the peripheral blood. As shown in Supplementary Fig. 26, compared to other groups, the microscopic examination of the structural integrity of the heart, liver, spleen, lungs, and kidneys in the aCD20@Exo^CTX^/siPDK4 group revealed no signs of inflammation or fibrosis, nor was there any evidence of atrophy, hyperplasia, necrosis, or inflammation. IL-6 is a well-recognized inflammatory mediator [49]. Therefore, the potential inflammatory effects of aCD20@Exo^CTX^/siPDK4 could be assessed by measuring the plasma levels of interleukin 6 (IL-6). As previously mentioned in the literature, the average level of IL-6 in the standard control group is approximately 30 pg/ml [50]. As shown in Supplementary Fig. 27, compared to the PBS control group, the IL-6 levels in rituximab-resistant mice treated with aCD20@Exo^CTX^/siPDK4 did not show significant changes at 24 h, 7 days, 14 days, and 21 days post-treatment. Additionally, compared to other treatment groups, the leukocyte, erythrocyte, hemoglobin, and platelet counts in tumor-bearing mice treated with aCD20@ExoCTX/siPDK4 showed no significant decrease. Furthermore, there were no abnormal changes in ALT, AST, TP, BUN, CRE, TBIL, DBIL, CK, and Myo levels; all tested parameters remained within the normal range (Supplementary Fig. 28). These results confirm that the intravenous injection of the exosome nanoparticle medicine aCD20@Exo^CTX^/siPDK4, which we designed and constructed, is safe and does not induce acute or chronic toxicity.

## Discussion

Although rituximab significantly improves the clinical prognosis of DLBCL patients, many still relapse after receiving R-CHOP therapy, and many of these patients are challenging to cure. Acquired resistance to rituximab is a crucial reason for the recurrence and refractoriness of DLBCL. Studies have shown that the expression of CD20 protein in DLBCL cells is downregulated after applying rituximab [51, 52]. Our various experiments have demonstrated that rituximab can significantly downregulate the expression of CD20 protein in DLBCL cells and further confirmed that PDK4 expression in DLBCL cells is associated with rituximab resistance. The expression of PDK4 and CD20 is negatively correlated, where PDK4 suppresses the expression of CD20 through transcriptional inhibition, thus regulating the sensitivity of DLBCL cells to rituximab. Subsequently, we proved that PDK4 is primarily located in the nucleus in rituximab-resistant DLBCL patient tissues and rituximab-resistant DLBCL cell lines. In these contexts, PDK4 activates HDAC8 by phosphorylating the Ser-39 site of nuclear HDAC8, which deacetylates and inhibits the expression of CD20 protein, inducing rituximab resistance in DLBCL cells. Therefore, this study discovers a new role for PDK4 as a kinase in the nucleus and reveals how PDK4 regulates CD20 expression in diffuse large B-cell lymphoma cells for the first time, leading to rituximab resistance. This provides a new direction for the development of novel targeted drugs.

**Fig. 10:**
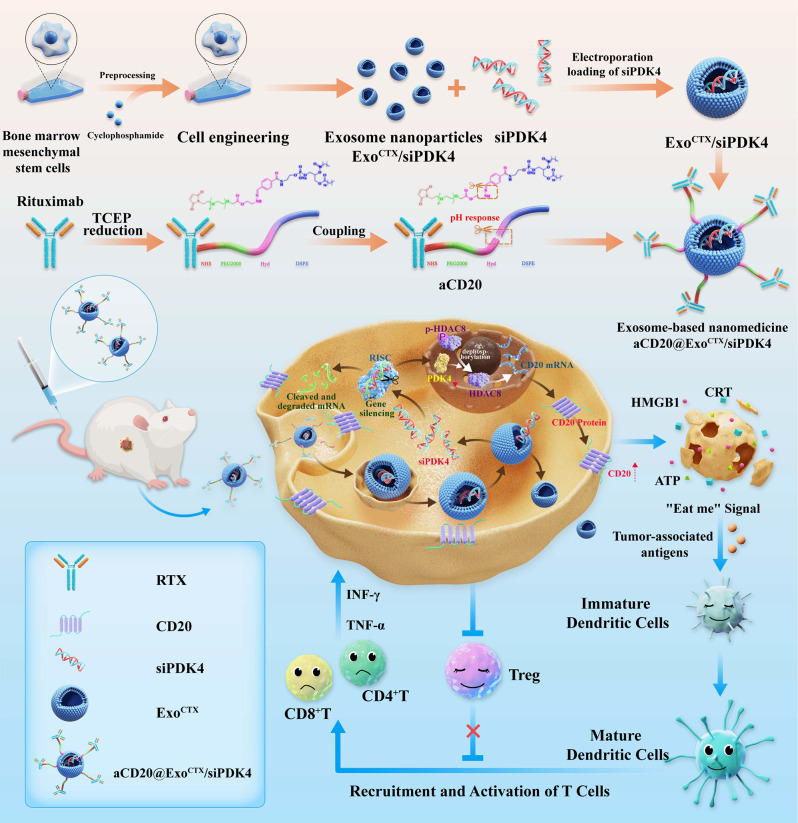
Schematic diagram of aCD20@ExoCTX/siPDK4 construction and its targeted therapeutic mechanisms in DLBCL

In this study, we designed and constructed a “smart” exosome-based targeted delivery strategy to reverse PDK4-mediated rituximab resistance in DLBCL **(**Fig. 10**)**. In our research, we achieved targeted delivery of rituximab and intracellular delivery of siPDK4, enhancing the expression of the membrane protein CD20 by inhibiting the PDK4/HDAC8/CD20 signaling pathway, ultimately eliminating the pathogenicity and resistance of DLBCL cells. siRNA has a short half-life, weak membrane penetration ability, can be rapidly degraded in circulation, and has very low stability [9], which complicates the therapeutic targeting of diffuse large B-cell lymphoma, especially in recurrent/refractory DLBCL patients and resistant cells where CD20 antigen expression is reduced (or lost), making targeting with rituximab (RTX) more challenging. [14]。Therefore, constructing an effective carrier is crucial for successful delivery. To address this challenge, we have adopted exosomes as the delivery vehicle, which offer numerous advantages such as high biocompatibility, low immunogenicity, inherent cell-targeting capabilities, and robust drug-carrying capacity. Compared to other antibody and siPDK4 delivery methods, aCD20@Exo^CTX^/siPDK4 offers several advantages: (1) Leveraging the intrinsic active targeting ability of exosomes allows for efficient delivery of rituximab and PDK4 siRNA; (2) Utilizing pH-responsive chemical bond cleavage, rituximab is designed as a “gatekeeper” molecule, enabling drug release responsive to environmental conditions; (3) By “re-educating” parent cell-derived exosomes, they are endowed with the ability to induce immunogenic cell death (ICD) and immunosuppressive functions, facilitating synergistic anti-tumor immunotherapy. aCD20@Exo^CTX^/siPDK4 is not eliminated in the bloodstream. Fluorescence imaging of mouse organs and tumor tissues shows that after modification, the exosome delivery vehicle has enhanced targeting of DLBCL-resistant cells. Additionally, routine blood tests, biochemical indicators in the peripheral blood, and histological H&E staining results demonstrate that exosome-based nanomedicine exhibits excellent biocompatibility in mice. This increased targeting capability and proven biocompatibility highlight the potential of aCD20@Exo^CTX^/siPDK4 as an effective and safe therapeutic option for targeting resistant DLBCL cells.

Our study demonstrated that treatment with aCD20@Exo^CTX^/siPDK4 effectively promotes rituximab sensitivity, inhibits the proliferation of resistant DLBCL cells, and induces apoptosis, showing excellent anti-tumorigenic efficacy in vitro. To prove the efficacy of the exosome-based nanomedicine in a DLBCL-resistant tumor model, results indicated that aCD20@Exo^CTX^/siPDK4 effectively downregulated PDK4 gene expression in vivo, enhancing the antitumor activity of rituximab and thereby inhibiting the generation of DLBCL tumor cells. Moreover, our in vitro studies showed that aCD20@Exo^CTX^/siPDK4-induced ICD leads to exposure of CRT, the release of HMGB1 and ATP, thus activating dendritic cells (DCs) against a broad range of tumors and increasing the proportion of CD8^+^ T cells, providing an immunotherapeutic effect. In vivo results showed that aCD20@Exo^CTX^/siPDK4 helps to prolong circulation time in the body and enhance fusion with tumor cells, thus ensuring the accumulation of rituximab and siPDK4 at the tumor site. Furthermore, aCD20@Exo^CTX^/siPDK4 exosome nanoparticles promote the activation of DCs and initiate an adaptive immune response. In the antigen presentation process, the maturation of DCs plays a crucial role and does not require the addition of immune stimulants, thereby amplifying the efficacy of chemo-immunotherapy. This enhances the infiltration of effector T cells (CD4^+^ and CD8^+^ T cells) into the tumor, promoting the release of pro-inflammatory cytokines (TNF-α, IL-6, and IL-12), killing the cancer and reversing tumor resistance. These results suggest that aCD20@Exo^CTX^/siPDK4 can promote high expression of the membrane protein CD20, thus providing a promising therapeutic tool for improving outcomes in DLBCL patients with negative or low CD20 expression.

## Conclusions

In summary, this study reveals that PDK4 induces resistance to rituximab in DLBCL by regulating CD20 expression through the phosphorylation of nuclear HDAC8. By highlighting the potential value of PDK4 as a promising drug target for treating DLBCL resistance, our work provides deeper insights into the resistance mechanisms of DLBCL. Subsequently, our developed “smart” exosome-based targeting strategy can induce apoptosis in DLBCL-resistant cells and promote effective immunogenic cell death (ICD), thereby reversing the immunosuppressive TME, showing a synergistic therapeutic effect in subcutaneous mouse tumor resistance models. Therefore, due to its inherent biocompatibility, precise structure and composition, and highly effective therapeutic results, this type of exosome-based nanomedicine holds excellent promise for future clinical translation in reversing DLBCL resistance, opening new avenues in drug delivery.

### Electronic supplementary material

Below is the link to the electronic supplementary material.

Supplementary Material 1

## Data Availability

No datasets were generated or analysed during the current study.

## Abbreviations

DLBCL: Diffuse Large B-cell Lymphoma
PDK4: Pyruvate Dehydrogenase Kinase 4
CD20: Membrane Spanning 4-Domains A1
shRNA: short hairpin RNA
HDAC8: Histone Deacetylase 8
DLS: Dynamic Light Scattering
TEM: Transmission Electron Microscopy
NHL: Non-Hodgkin Lymphoma
R: CHOP-Rituximab, Cyclophosphamide, Doxorubicin, Vincristine, Prednisone
CAR: T-Chimeric Antigen Receptor T-cell
siRNA: Small Interfering RNA
Exo: Exosomes
BMSCs: Bone Marrow Mesenchymal Stem Cells
RTX: Rituximab
CTX: Cyclophosphamide
KEGG: Kyoto Encyclopedia of Genes and Genomes
Co: IP-Co-Immunoprecipitation
sh: NC-Short hairpin RNA Negative Control
NMR: Nuclear Magnetic Resonance
FTIR: Fourier-Transform Infrared Spectroscopy
PDI: Polydispersity Index
UV: visible-Ultraviolet-visible Spectroscopy
FCM: Flow Cytometry
CLSM: Confocal Laser Scanning Microscopy
PARP: Poly ADP ribose polymerase
ICD: Immunogenic Cell Death
DCs: Dendritic Cells
APCs: Antigen-Presenting Cells
CRT: Calreticulin
HMGB1: High Mobility Group Box 1
ATP: Adenosine Triphosphate
TME: Tumor Microenvironment
Tregs: Regulatory T cells
HVGs: Highly Variable Genes
PCA: Principal Component Analysis
t: SNE-t-Distributed Stochastic Neighbor Embedding
GO: Gene Ontology
GSVA: Gene Set Variation Analysis
GSEA: Gene Set Enrichment Analysis
ELISA: Enzyme-Linked Immunosorbent Assay
TUNEL: Terminal deoxynucleotidyl transferase dUTP nick end labeling
