# Supplementary material for: Unveiling the PDK4-centered rituximab-resistant mechanism in DLBCL: the potential of the “Smart” exosome nanoparticle therapy
Source: Mol Cancer. 2024 Jul 15;23:144. doi: 10.1186/s12943-024-02057-0 (PMC11247735; doi:10.1186/s12943-024-02057-0)
Supplement: Supplementary file 1 — Supplementary Material 1 [file 12943_2024_2057_MOESM1_ESM.doc]

**Supplementary materials**

Table of Contents

[1. Experimental Procedure 3](#__RefHeading___Toc168313802)

[**1.1 Immunohistochemistry of DLBCL Patients** 3](#__RefHeading___Toc168313803)

[**1.2**  3](#__RefHeading___Toc168313804)

[**Construction and validation of the PDK4 gene silencing system** 3](#__RefHeading___Toc168313805)

[**1.3 Transcriptome Sequencing** 4](#__RefHeading___Toc168313806)

[**1.4 Real-time fluorescence quantitative PCR was used to detect the mRNA expression levels in cells and tumor tissues** 5](#__RefHeading___Toc168313807)

[**1.5 Co-immunoprecipitation (Co-IP)** 7](#__RefHeading___Toc168313808)

[**1.6 Prediction of Protein Interactions** 7](#__RefHeading___Toc168313809)

[**1.7 Isolation and Culture of Human Bone Marrow Mesenchymal Stem Cells** 7](#__RefHeading___Toc168313810)

[**1.8** **Western Blot Verification of Exosomal Surface Protein Expression** 8](#__RefHeading___Toc168313811)

[**1.9 Effects of Different Concentrations of Cyclophosphamide on the Viability of Bone Marrow Mesenchymal Stem Cells and Preparation of Exo^CTX^** 8](#__RefHeading___Toc168313812)

[**1.10 Determination of hydrodynamic diameter (HD), polydispersity index (PDI) and zeta potential** 9](#__RefHeading___Toc168313813)

[**1.11 Flow Cytometry Analysis of Exosome Internalization After Cyclophosphamide Pretreatment** 9](#__RefHeading___Toc168313814)

[**1.12** **Public Database Sources** 10](#__RefHeading___Toc168313815)

[**1.13 scRNA-seq Data Quality Control and Analysis** 10](#__RefHeading___Toc168313816)

[**1.14 Correlation Between Drug Sensitivity IC50 and PDK4 Gene Expression** 11](#__RefHeading___Toc168313817)

[**1.15 Deconvolution Analysis** 11](#__RefHeading___Toc168313818)

[**1.16 Cell Communication Analysis** 12](#__RefHeading___Toc168313819)

[**1.17 Differential Analysis and Pathway Analysis** 12](#__RefHeading___Toc168313820)

[**1.18 Single-cell trajectory analysis.** 12](#__RefHeading___Toc168313821)

[**1.19 PET and CT (PET/CT) Scanning for Whole-Body Imaging in Mice** 13](#__RefHeading___Toc168313822)

[**1.20 Hematoxylin and Eosin (H&E) Staining of Tumor Tissues and Organs** 13](#__RefHeading___Toc168313823)

[**1.21 Tissue Immunofluorescence Staining and Immunohistochemistry Testing** 14](#__RefHeading___Toc168313824)

[**1.22 Western Blotting for Protein Expression in Tumor Tissues** 15](#__RefHeading___Toc168313825)

[**1.23 Preparation of Single-Cell Suspension from Tumor Tissue** 15](#__RefHeading___Toc168313826)

[**1.24 Cytokine detection** 15](#__RefHeading___Toc168313827)

[2.Result 16](#__RefHeading___Toc168313828)

[2.1 RNA-Seq Analysis of PDK4 as a Potential Regulator of CD20 Expression in Resistant Cells 16](#__RefHeading___Toc168313829)

[2.2 Validation of Bioinformatics Results by qRT-PCR 19](#__RefHeading___Toc168313830)

[2.3 Effect of Different Concentrations of Cyclophosphamide on the Viability of Bone Marrow Mesenchymal Stem Cells 22](#__RefHeading___Toc168313831)

[2.4 Flow Cytometry Analysis of Exo^CTX^ Internalization 23](#__RefHeading___Toc168313832)

[2.5 Loading Efficiency and Degradation Resistance of Exo^CTX^ Loaded with siPDK4. 24](#__RefHeading___Toc168313833)

[2.6 Flow Cytometry Analysis of Exo^CTX^/siPDK4 Transfection Efficiency 25](#__RefHeading___Toc168313834)

[2.7 Characterization Results of mAb(Rituximab)-NHS-PEG2000-Hyd-DSPE 27](#__RefHeading___Toc168313835)

[2.8 Fluorescence Spectroscopy Analysis of Cy5 Labeled on DSPE-Hyd-PEG2000-NHS-Rituximab 28](#__RefHeading___Toc168313836)

[2.9 In Vitro Release Characteristics of aCD20@Exo^CTX^/siPDK4 Nanoparticles 29](#__RefHeading___Toc168313837)

[2.10 In Vitro Hemocompatibility, Stability, and Safety Assessment of aCD20@Exo^CTX^/siPDK4 Nanoparticles 30](#__RefHeading___Toc168313838)

[2.11 In Vitro Induction of Apoptosis in SU-DHL-2 and SU-DHL-2/R Cells by aCD20@Exo^CTX^/siPDK4 31](#__RefHeading___Toc168313839)

[2.12 In Vitro Expression of Bcl-2, Cleaved PARP, Cleaved Caspase-3, PDK4, HDAC8, Phosphorylated HDAC8, and CD20 Proteins Induced by aCD20@Exo^CTX^/siPDK4 32](#__RefHeading___Toc168313840)

[2.13 Quality control analysis for the Diffuse sizeable B-cell lymphoma single-cell RNA-seq database. 33](#__RefHeading___Toc168313841)

[2.14 Identification of major cell types and malignant B cells and their corresponding subpopulations analysis. 34](#__RefHeading___Toc168313842)

[2.15 PDK4's role in reshaping the tumor microenvironment (TME) in DLBCL. 36](#__RefHeading___Toc168313843)

[2.16 PDK4^+^ Tregs Play a Crucial and Efficient Role in Cellular Communication 37](#__RefHeading___Toc168313844)

[2.17 Significant Impact of PDK4 on the Biological Functions of Tregs 38](#__RefHeading___Toc168313845)

[2.18 PDK4 Promotes the Differentiation of Tregs into More Suppressive Subtypes 39](#__RefHeading___Toc168313846)

[2.19 Statistical Data on In Vitro CRT Expression, DC Maturation, and CD8+ T Cell Expression 40](#__RefHeading___Toc168313847)

[2.20 Scheme for the generation of the humanized mouse model 41](#__RefHeading___Toc168313848)

[2.21 Immunofluorescence Detection of Apoptosis Protein Expression Induced by aCD20@Exo^CTX^/siPDK4 In Vivo 42](#__RefHeading___Toc168313849)

[2.22 Statistical Data on In Vivo DC Maturation, Expression of CD3^+^ T Cells, CD8^+^ T Cells, and Treg Cells 43](#__RefHeading___Toc168313850)

[2.23 Proliferation, Differentiation, Maturation, or Activation of Immune Cells Induced by BMSC-Derived Exosomes 44](#__RefHeading___Toc168313851)

[2.24 Flow Cytometry Analysis of the Proportion of Tregs (CD4^+^CD25^+^Foxp3^+^) in Healthy Human Controls and Human DLBCL Patients 45](#__RefHeading___Toc168313852)

[2.25 Detection of Cytokines in Mouse Serum 46](#__RefHeading___Toc168313853)

[2.26 H&E Staining of Key Organs in Mice 47](#__RefHeading___Toc168313854)

[2.27 Determining the potential inflammatory effects of aCD20@Exo^CTX^/siPDK4 through the levels of interleukin-6 (IL-6) in plasma. 48](#__RefHeading___Toc168313855)

[2.28 Hematological and biochemical tests of mouse blood. 49](#__RefHeading___Toc168313856)

[3. References 50](#__RefHeading___Toc168313857)

# Experimental Procedure

**1.1 Immunohistochemistry of DLBCL Patients**

Paraffin-embedded slides were baked at 70 ℃ for 1 h and then successively placed in xylene, absolute ethanol, 90% ethanol, 80% ethanol, 70% ethanol, and PBS for deparaffinization and rehydration. Antigen retrieval was performed in a pressure cooker with citrate antigen retrieval solution (pH = 6.0). After cooling, the retrieval solution was washed off with PBS. Sections were blocked at room temperature for 20 min, then incubated overnight at 4℃ with PDK4 antibody (diluted 1:1000 in antibody dilution buffer). After washing the slides 3 times with 0.1% PBST, they were incubated with Alexa Fluor 555 labeled IgG antibody (diluted 1:600 in antibody dilution buffer) at room temperature for 20 min. After washing off the antibodies with 0.1% PBST, slides were mounted with a mounting medium containing DAPI and observed under a fluorescence microscope.

**1.2** **Construction and validation of the PDK4 gene silencing system**

shRNA section：One pairs of shRNA sequences (Tsingke Biotechnology) targeting human PDK4 were annealed and ligated into the Plk0.1‐puro lentiviral vector. The targeting sequences of PDK4 shRNA were 5′‐ACTGCAACGTCTCT GAGGTG‐3′. A scrambled shRNA was used as a control. Human PDK4 coding sequence (Vigene Bioscience) was cloned into the pcDNA3.1 plasmid to generate a pcDNA/PDK4 expression plasmid. The pcDNA3.1 was used as empty vector control for analysis. To generate PDK4 stable knockdown and overexpression cells, recombinant lentivirus was produced by transient transfection of HEK293T cells. The transfection efficacy was determined by qRT‐PCR and western blot analysis. siRNA Section: Logarithmically growing SU-DHL-2/R and OCI-LY8/R cells were seeded in 6-well plates. When they reached 70-80% confluence, transfection was performed according to the Lip3000 protocol, using the siRNA sequence (PDK4-siRNA-GAUGCUAUCAUCUACUUAAAG). Six hours post-transfection, the cells were placed in fresh medium and cultured for an additional 24 hours. Subsequently, the cells were harvested, and the knockdown efficiency of the siRNA was verified using WB and qPCR. One pair of shRNA sequences (Tsingke Biotechnology) targeting human PDK4 were annealed and ligated into the Plk0.1‐puro lentiviral vector. The targeting sequences of PDK4 shRNA were 5′‐ACTGCAACGTCTCT GAGGTG‐3′. A scrambled shRNA was used as a control. The human PDK4 coding sequence (Vigene Bioscience) was cloned into the pcDNA3.1 plasmid to generate a pcDNA/PDK4 expression plasmid. The pcDNA3.1 was used as an empty vector control for analysis. To generate PDK4 stable knockdown and overexpression cells, recombinant lentivirus was produced by transient transfection of HEK293T cells. QRT-PCR and western blot analysis determined the transfection efficacy.

**1.3 Transcriptome Sequencing**

Total RNA from the SU-DHL-2/R cells in both sh-NC and sh-PDK4 groups was extracted using the TRIzol (Invitrogen) method. The purity and concentration of the extracted RNA were assessed with the NanoDrop 2000 spectrophotometer. RNA integrity was evaluated through agarose gel electrophoresis with an Agilent Bioanalyzer 2100. All samples passed the quality assessment. Following the manufacturer's recommendations, sequencing libraries were generated with a Hieff NGS Ultima Dual-mode mRNA Library Prep Kit for Illumina. The library quality was assessed on the Agilent Bioanalyzer 2100 system. Subsequently, the samples were sequenced on an Illumina NovaSeq6000 platform by the Wuhan Huada Gene Detection Center. Differential gene analysis was performed using the DE-seq2 method with a selection threshold of |log2FC| ≥ 1 and FDR < 0.05. Based on the differential gene detection results, further analysis was conducted on the candidate genes.

**1.4 Real-time fluorescence quantitative PCR was used to detect the mRNA expression levels in cells and tumor tissues**

RNAiso plus was used for mRNA extraction from tumor tissue samples and cells, and the reverse transcription kit was used for cDNA synthesis using the appropriate primers, including GAB1、RAC3、HDAC8、CHKB、MGST2、KMT2A、PTPN6、DDIT3、FZD7、LNPEP、REV3L、PIK3R1、BRCA1、MDM2、SSR4、APAF1、BIRC3、MET、JUN、NCOA3、PDK4 and CD20. The used primer sequences are listed in Supplementary Table 1. The reaction conditions were as follows: 95 °C for 10 min, 95 °C for 10 s, 60 °C for 10 s, 72 °C for 10 s, and 45 amplification cycles. GAPDH was used as the internal control. The Cq value of each reaction was recorded, and the double Δ method was used to calculate the data.

| Supplementary Table 1. Primers used for the qRT-PCR analysis. | |
| --- | --- |
| Primer name | Sequence (5'→3') |
| GAB1-forward  GAB1-reverse  RAC3-forward  RAC3-reverse  HDAC8-forward  HDAC8-reverse  CHKB-forward  CHKB-reverse  MGST2-forward  MGST2-reverse  PTPN6-forward  PTPN6-reverse  DDIT3-forward  DDIT3-reverse  FZD7-forward  FZD7-reverse  LNPEP-forward  LNPEP-reverse  REV3L-forward  REV3L-reverse  PIK3R1-forward  PIK3R1-reverse  BRCA1-forward  BRCA1-reverse  MDM2-forward  MDM2-reverse  APAF1-forward  APAF1-reverse  BIRC3-forward  BIRC3-reverse  MET-forward  MET-reverse  JUN-forward  JUN-reverse  NCOA3-forward  NCOA3-reverse  SSR4-forward  SSR4-reverse  KMT2A-forward  KMT2A-reverse  PDK4-forward | CAGCGAGGAGGAGATGAATAAG  TGGATGGTGGTGCTGTATTT  GACGACAAGGACACCATTGA  GCTGAGCACTCCAGGTATTT  TTTGGGAGGAGGAGGCTATAA  CTGGGATCTCAGAGGATAGTGT  CTCTGGCATCCCATTTCTTCT  CTGGGCATAGTCCAAGTAACC  AGAGAGTATTTCGGGCACAAC  CAGACCCAGACAAGTAGCAAA  TGGGAGGAGTTTGAGAGTTTG  GGTTCTTGCCCTTGTTCTCT  TCACTCTCCAGATTCCAGTCA  ACCACTCTGTTTCCGTTTCC  CTTCATAGGCACGTCCTTCTT  CATGAGCTTCTCCAGCTTCT  GTTGTGCCACTACGCTATGA  CCTGAAGAGCCTGAACTGAAA  GGGCGGAAAGGCACTATTT  AGGTTGGCTCCGACATTTAC  GCTTTGCCGAGCCCTATAA  ACATTGAGGGAGTCGTTGTG  CAGTCGGGAAACAAGCATAGA  GCACATTCCTCTTCTGCATTTC  GCCTGGCTCTGTGTGTAATAA  CCCTGCCTGATACACAGTAAC  TCAAAGCTGAAACAGGAGAGAA  CCACTGAGCAGGTTGCTATAA  TGCTCGTGCTGGTTTCTATT  TCAGTAGGACTGTCTCCTCTTT  TCGTGCTCCTGTTTACCTTG  GGTGAAGTTGGGAAGCTGATA  TTCTATGACGATGCCCTCAAC  TCAGGGTCATGCTCTGTTTC  CGGCAGAATGGAACCTATGAA  CGAAGAGGCAATGTGGGAATA  CAGGAAGGCTCAGAGGAATAAC  GGCCAAGTAGTAGATCACAAGG  AGCTGTCCAAAGATCGAGATG  TTCCTTGACTCCCGCTTATTC  CACGATGTGAATTGGTTGGT |
| PDK4-reverse | TGCCTTTGAGTGTTCAAGGA |
| MS4A1-forward | ATGAAAGGCCCTATTGCTATG |
| MS4A1-reverse | GCTGGTTCACAGTTGTATATG |
| β-actin-forward | GGACTTCGAGCAAGAGATGG |
| β-actin-reverse | AGCACTGTGTTGGCGTACAG |

**1.5 Co-immunoprecipitation (Co-IP)**

Cellular proteins were extracted with the Cell lysis buffer for IP (Beyotime). Proteins (500 μg) were incubated overnight at 4 °C with anti-PDK4 anti-HDAC8 or anti-CD20 antibody or a control IgG. The immune complexes were immunoprecipitated with Protein A/G Plus-Agarose beads (Santa Cruz), washed thrice with TBST, subjected to 12% SDS-PAGE, and detected PDK4, HDAC8, and CD20.

**1.6 Prediction of Protein Interactions**

The X-ray crystal structures of PDK4 (2E0A), HDAC8 (1T64), and CD20 (6VJA) were obtained from the Protein Data Bank. To ensure the accuracy of the docking results, the two protein structures were manually prepared by removing water and adding hydrogens using AutoDockTools-1.5.7^1^. Subsequently, protein-protein docking was performed using the docking server (ZDock)^2, 3^. The resulting protein-protein complexes were then similarly prepared by removing water and adding hydrogens using AutoDockTools-1.5.7. Finally, the protein interactions were predicted and visualized using PyMOL to generate protein-protein interaction diagrams.

**1.7 Isolation and Culture of Human Bone Marrow Mesenchymal Stem Cells**

During total hip arthroplasty, bone marrow was collected from the femoral shaft marrow cavity of patients. 5 mL of the collected bone marrow and 5 mL of serum-free culture medium for human bone marrow mesenchymal stem cells (with 2% human platelet lysate) were mixed by pipetting in a centrifuge tube and then inoculated into a 10 cm culture dish. After 48 hours, half of the medium was replaced with a complete culture medium, and after 72 hours, all medium was replaced to remove non-adherent cells. Cell growth was monitored under a microscope after each medium change. When cell confluence reached about 90%, the cells were passaged at 1:3. Third-generation bone marrow mesenchymal stem cells were used for surface antigen detection. Cells were digested with 0.25% trypsin, and the cell suspension was transferred to EP tubes. Fluorescently labeled monoclonal antibodies were added to each tube, with isotype and negative controls set up simultaneously. The samples were incubated in the dark at 4°C for 20 minutes and then washed with PBS. Analysis was performed on a flow cytometer. This study was approved by the Ethics Committee of Qinghai Provincial People’s Hospital [Ethics approval number: 2022-19], and the patients' bone marrow samples were collected with written consent.

**1.8** **Western Blot Verification of Exosomal Surface Protein Expression**

Take 50 µL of exosome suspension, add an appropriate amount of RIPA lysis buffer, and mix well. After complete lysis, obtain the exosome protein suspension and aliquot and store it at -80 °C for later use. Quantitatively measure the exosome protein concentration using a BCA protein assay kit. Load 20 µL per well and add 5 µL of pre-stained protein marker. Start the electrophoresis at 100 V to monitor the gel running speed and bands' migration. Adjust the voltage as necessary. Electrophorese the stacking gel at 80 V for 30 minutes, then continue with the separating gel at 100 V until the bromophenol blue marker reaches the bottom edge of the gel. Transfer the protein to a PVDF membrane at a constant current of 300 mA for 90 minutes. Block the membrane at room temperature with 5% TBST-skimmed milk for 1 hour, then incubate it overnight at 4 °C with primary antibody dilutions (CD9, CD63, both diluted 1:1000) in TBST. Wash the PVDF membrane three times with TBST, each for 10 minutes. Add diluted HRP-conjugated secondary antibody and incubate at room temperature for 1 hour. Wash the membrane three times with TBST, each for 10 minutes. Mix high-sensitivity ECL solutions A and B in equal proportions, cover the PVDF membrane adequately with the mixture, expose, and capture the image.

**1.9 Effects of Different Concentrations of Cyclophosphamide on the Viability of Bone Marrow Mesenchymal Stem Cells and Preparation of Exo^CTX^**

To determine the optimal working concentration of cyclophosphamide (CTX) that does not affect the vitality of bone marrow mesenchymal stem cells (BMSCs) in our study, based on previous research^4^， BMSCs were incubated with CTX at concentrations of 0 μmol/L, 75 μmol/L, 125 μmol/L, 250 μmol/L, 500 μmol/L, and 1000 μmol/L for 24 hours. Cell viability was then assessed using the CCK-8 assay. The preparation of Exo^CTX^ was slightly modified from previous studies^5^。In brief, 1×10^8 BMSCs cultured in a medium containing 500 μmol/L cyclophosphamide were exposed to ultraviolet B (UVB) radiation (300J/m^2) for 1 hour in a biosafety cabinet, followed by an additional 18 hours of culture. The supernatant was collected and centrifuged at 300×g for 15 minutes and then at 2000×g for 20 minutes to remove cells and cell debris. Subsequently, the supernatant was centrifuged at 10,000×g for 30 minutes and then twice at 100,000×g, each time for approximately 1 hour. Finally, the precipitate was resuspended in PBS and filtered through a 0.22 μm filter to obtain the exosomes (Exo^CTX^).

**1.10 Determination of hydrodynamic diameter (HD), polydispersity index (PDI) and zeta potential**

The physicochemical parameters, including hydrodynamic diameter, PDI, and zeta potential of aCD20@Exo^CTX^/siPDK4, were recorded over the period of 6 days by dynamic light scattering (DLS) using Zeta Sizer. All measurements were carried out after dilution (1:20) with distilled water.

**1.11 Flow Cytometry Analysis of Exosome Internalization After Cyclophosphamide Pretreatment**

In brief, according to previous reports, the transfection efficiency of Exo^CTX^/siPDK4 was evaluated using flow cytometry^6^. Initially, exosomes (5 μg/well) were stained with PKH-67 according to the manufacturer's instructions. Log-phase growing SU-DHL-2/R and OCI-LY8/R cells were seeded at 2×10^5 cells/well in a 6-well plate. After overnight incubation for 24 hours, the cells were treated with PKH67-labeled Exo^CTX^/siPDK4. The transfection occurred at 37°C and 5% CO_2_ for 24 hours. Cells were then collected by centrifugation at 800 × g for 5 minutes and resuspended in 200 μL of PBS. Flow cytometry was used to analyze and calculate the percentage of cells positive for PKH67 fluorescence, thus determining the transfection efficiency.

**1.12** **Public Database Sources**

Public datasets of diffuse large B-cell lymphoma were retrieved from the Gene Expression Omnibus (GEO) and heiDATA databases. Based on the clinical information associated with the samples, the selected public datasets for the study include GSE182434, heiDATA-VRJUNV, GSE252455, GSE252608, and GSE10846^7-10^.

**1.13 scRNA-seq Data Quality Control and Analysis**

The Seurat R package (version 4.1.3) was used for reading, quality controlling, dimension reduction, and clustering of scRNA-seq data. Cells were filtered based on the criteria of fewer than 200 genes and mitochondrial gene expression ratios greater than 10% to eliminate low-quality cells. The filtered cells were then normalized using the "lognormalize" method. The FindVariableFeatures function was used to identify highly variable genes, which were used for subsequent PCA for dimension reduction. Cell clustering was performed using the FindClusters function in Seurat (resolution=1), followed by UMAP (Uniform Manifold Approximation and Projection) and t-SNE (t-Distributed Stochastic Neighbor Embedding) analyses for dimension reduction and visualization. Specific genes for each cluster were identified using the FindAllMarkers function. Cluster relationships at various resolutions were visualized using the clustree visualization tool. Classical marker genes were used to annotate different cell subtypes. Based on the cell annotation results, ClusterGVis was employed for heatmap and enrichment analysis display of differential genes among cell subgroups. The Nebulosa and scCustomize packages were used to visualize marker genes. InferCNV is used for CNV analysis, with CD4+ T cells serving as normal controls. Specific parameter settings and criteria for evaluating tumor cells refer to previous studies ^11^.

**1.14 Correlation Between Drug Sensitivity IC50 and PDK4 Gene Expression**

The OncoPredict R package was utilized to predict in vivo drug responses in DLBCL patients^12^. OncoPredict integrates tissue gene expression profiles with drug IC50 data, which represents the concentration of a drug needed to inhibit biological activity by half, sourced from the Genomics of Drug Sensitivity in Cancer (GDSC; https://www.cancerrxgene.org/). This data is fitted against gene expression from the Cancer Cell Line Encyclopedia (CCLE; https://portals.broadinstitute.org/ccle_legacy/home) profiles. A total of 198 drugs were analyzed. The relationship between drug IC50 values and PDK4 gene expression was assessed using Spearman correlation analysis. Significant correlations were considered for coefficients greater than 0.2 and an FDR (False Discovery Rate) less than 0.05.

**1.15 Deconvolution Analysis**

CIBERSORTx is a machine learning toolkit that quantifies cell type abundances from bulk RNA sequencing and microarray data^13^. We used the previously annotated single-cell RNA-seq (scRNA-seq) dataset to construct a scRNA-seq feature matrix with CIBERSORTx. After formatting and uploading the single-cell reference matrix file according to the instructions, we ran the "Create Feature Matrix" module to build the scRNA-seq feature matrix. Using the generated signature matrix, we then performed CIBERSORTx deconvolution on the GSE10846 dataset. Pearson correlation coefficients were used to assess the relationship between PDK4 gene expression and immune cell types. The R package "ggplot2" was used for visualization. Survival analysis based on different immune cell proportions in patients was conducted using the R language package "survival." Patients were divided into high and low-immune cell proportion groups for survival curve analysis. The Log-Rank test was employed to determine if the survival curves were statistically significant, with a P-value < 0.05 considered statistically significant.

**1.16 Cell Communication Analysis**

CellChat is employed to analyze intercellular communication^14^. A CellChat object was initially created using the "createCellChat" function based on the RNA expression matrix and cell metadata. Subsequently, downstream analysis incorporated expression of ligand-receptor (L-R) interactions, including "Secreted Signaling," "ECM-Receptor," and "Cell-Cell Contact." The "computeCommunProb" function was utilized to calculate the probabilities of communication and to infer the communication networks of cell interactions. The integrated cell communication networks were computed using the "aggregateNet" function. Network centrality scores within the communication networks were calculated and visualized using the "netAnalysis_computeCentrality" function. Visualization of the cell communication results was facilitated using functions like "netAnalysis_signalingRole_scatter," "netVisual_heatmap," and "netVisual_chord_cell," providing detailed insights into the dynamics of intercellular communication.

**1.17 Differential Analysis and Pathway Analysis**

Treg cells based on PDK4 expression were divided into high and low-expression groups, and differentially expressed genes (DEGs) were identified using default parameters. The DEGs were then subjected to Kyoto Encyclopedia of Genes and Genomes (KEGG) enrichment analysis using the clusterProfiler package. Significantly enriched KEGG pathways (P < 0.05) were selected to ascertain the primary biological functions enacted by the DEGs. Gene Set Enrichment Analysis (GSEA) and Gene Set Variation Analysis (GSVA) were performed using the respective R packages, GSEA and GSVA, to explore further and validate the functional implications of the identified DEGs in biological pathways and processes.

**1.18 Single-cell trajectory analysis.**

The R packages Monocle2 and Monocle3 are used for pseudotime ordering analysis of Treg cell subpopulations to map their branching developmental trajectories^15^. Based on genes differentially expressed between clusters, single-cell developmental trajectories are constructed in pseudotime. The Monocle2 analysis achieves dimension reduction using a double data rate tree (DDRTree). The trajectories corresponding to cell differentiation are visualized using the plot_cell_trajectory function, and critical genes are displayed in a heatmap using the plot_pseudotime_heatmap function. In the Monocle3 analysis process, dimension reduction is first performed using UMAP. The developmental trajectory of cells is then constructed using the learn_graph function. Finally, pseudotime values are assigned to each cell, illustrating their progression stages within the trajectory. This approach provides a detailed view of the dynamic processes involved in Treg cell differentiation and development.

**1.19 PET and CT (PET/CT) Scanning for Whole-Body Imaging in Mice**

In the DLBCL drug-resistant tumor subcutaneous mouse model, PET/CT scans are conducted post-treatment. Before scanning, mice are fasted for 8 hours. Before the intravenous injection of ^18F-FDG, mice are placed in a comfortable environment to maintain body temperature. ^18F-FDG is then administered via the tail vein. During the ^18F-FDG absorption period, mice are anesthetized in an induction chamber with 2% isoflurane and kept warm under anesthesia. Approximately 60 minutes later, the PET/CT scanning begins a 5-minute CT scan followed by a 10-minute PET scan. CT data is used for attenuation correction and to localize PET images. Based on the fused PET/CT images, the tumor area is delineated. A semi-quantitative analysis of the ^18F-FDG uptake in the region of interest (ROI) is performed to calculate the maximum standardized uptake value (SUVmax). The formula for SUVmax is SUVmax = maximum tissue activity concentration (μCi/mL) / injected dose (μCi) × body weight (g). This methodology provides crucial insights into tumor-bearing mice's metabolic activity and treatment response.

**1.20 Hematoxylin and Eosin (H&E) Staining of Tumor Tissues and Organs**

Primary organs (heart, liver, spleen, lungs, kidneys) and tumor tissues are fixed in a 4% paraformaldehyde solution for 24 hours. They are then dehydrated in a graded series of alcohols—75%, 80%, 95%, and 100% ethanol, each for 1 hour, followed by clearing in xylene for 2 hours. The tissues are then embedded in paraffin wax for 2 hours. Paraffin-embedded tissues are sectioned at 4-5 µm thickness using a semi-automatic microtome and mounted on glass slides for drying, which can be used for staining or stored in a 4°C refrigerator. Paraffin sections are baked in an oven at 56°C for 1 hour and then dewaxed in xylene for 15 minutes. After dewaxing, the sections are rehydrated in water for 5 minutes. Hematoxylin solution is applied to stain the tissues on the glass slides, followed by soaking in running tap water for 10 minutes and counterstaining with eosin for 1 minute. The slides are dehydrated again through a graded series of 75%, 95%, and 100% alcohol, cleared in xylene and mounted with a neutral resin under a coverslip. Once dry, the slides are ready for examination and photography under a microscope.

**1.21 Tissue Immunofluorescence Staining and Immunohistochemistry Testing**

The paraffin sections are dewaxed to water through xylene and a graded series of alcohols, followed by antigen retrieval. After antigen retrieval, the sections are circled and blocked with BSA for 30 minutes. Diluted primary antibodies are applied, and the slides are incubated overnight at 4°C in a humidified chamber. The slides are then washed thrice in PBS (pH 7.4) on a shaker, each lasting 5 minutes. Corresponding secondary antibodies are added, and the sections are incubated in the dark at room temperature for 50 minutes. After the secondary antibody application, the slides are washed three times in PBS (pH 7.4) on a shaker, each lasting 5 minutes. DAPI stain is then applied to counterstain the nuclei and incubated in the dark at room temperature for 10 minutes. The slides are washed three times in PBS (pH 7.4) on a shaker, each lasting 5 minutes, before examination under a confocal microscope. DLBCL patient tissue samples are fixed in neutral buffered formalin, routinely dehydrated, embedded in paraffin, and sectioned at 3 μm thickness. Necessary immunohistochemical staining is performed on these sections to assess the expression of specific markers.

**1.22 Western Blotting for Protein Expression in Tumor Tissues**

Tumor tissues are finely chopped and ground using liquid nitrogen. The ground tissue is then transferred to a 1.5 ml Eppendorf (EP) tube, and 800 µl of Lysis buffer mixed with PMSF (1:100) is added. The mixture is centrifuged at 4°C at 12,000 rpm for 10 minutes. The supernatant is transferred to a new EP tube, and a small amount is taken for BSA protein quantification. The proteins are denatured by heating at 100°C for 10 minutes and then subjected to SDS-PAGE. Load 15 µl of the sample per well. The electrophoresis starts at 80 V for the first 20 minutes, allowing the bands to enter the separating gel before increasing the voltage to 110 V. Continue electrophoresis until the bands of interest, as determined by the marker, reach the desired position, then stop the electrophoresis. This procedure ensures that the proteins are resolved effectively for accurate analysis.

**1.23 Preparation of Single-Cell Suspension from Tumor Tissue**

To prepare a single-cell suspension from tumor tissue, collect the tumor tissue in 2 ml of PBS buffer and finely mince it with ophthalmic scissors. It is essential to perform the entire process on ice to preserve sample viability, and all instruments used should be thoroughly sterilized to prevent contamination. After mincing, combine the tissue with an equal volume of digestion solution (containing 0.1 µg/ml of type I collagenase, type IV collagenase, and hyaluronidase) and incubate on a shaker at 37°C for 1.5 hours to facilitate digestion. Following digestion, strain the tissue mixture through a 400-mesh sieve to filter out undigested parts and obtain a single-cell suspension. Count the cells to determine the cell yield and viability. This process helps ensure that the cellular components of the tumor are adequately separated and viable for further analysis.

**1.24 Cytokine detection**

Serum samples were isolated and collected from mice after euthanasia of mice given different treatments of PBS, RTX, RTX+siPDK4, aCD20@Exo/siPDK4, and aCD20@Exo^CTX^/siPDK4. After centrifugation, Tumor necrosis factors (TNF-α), IFN-γ, IL-12, and IL-6 in serum were analyzed with ELISA kits.

# 2.Result

## 2.1 RNA-Seq Analysis of PDK4 as a Potential Regulator of CD20 Expression in Resistant Cells


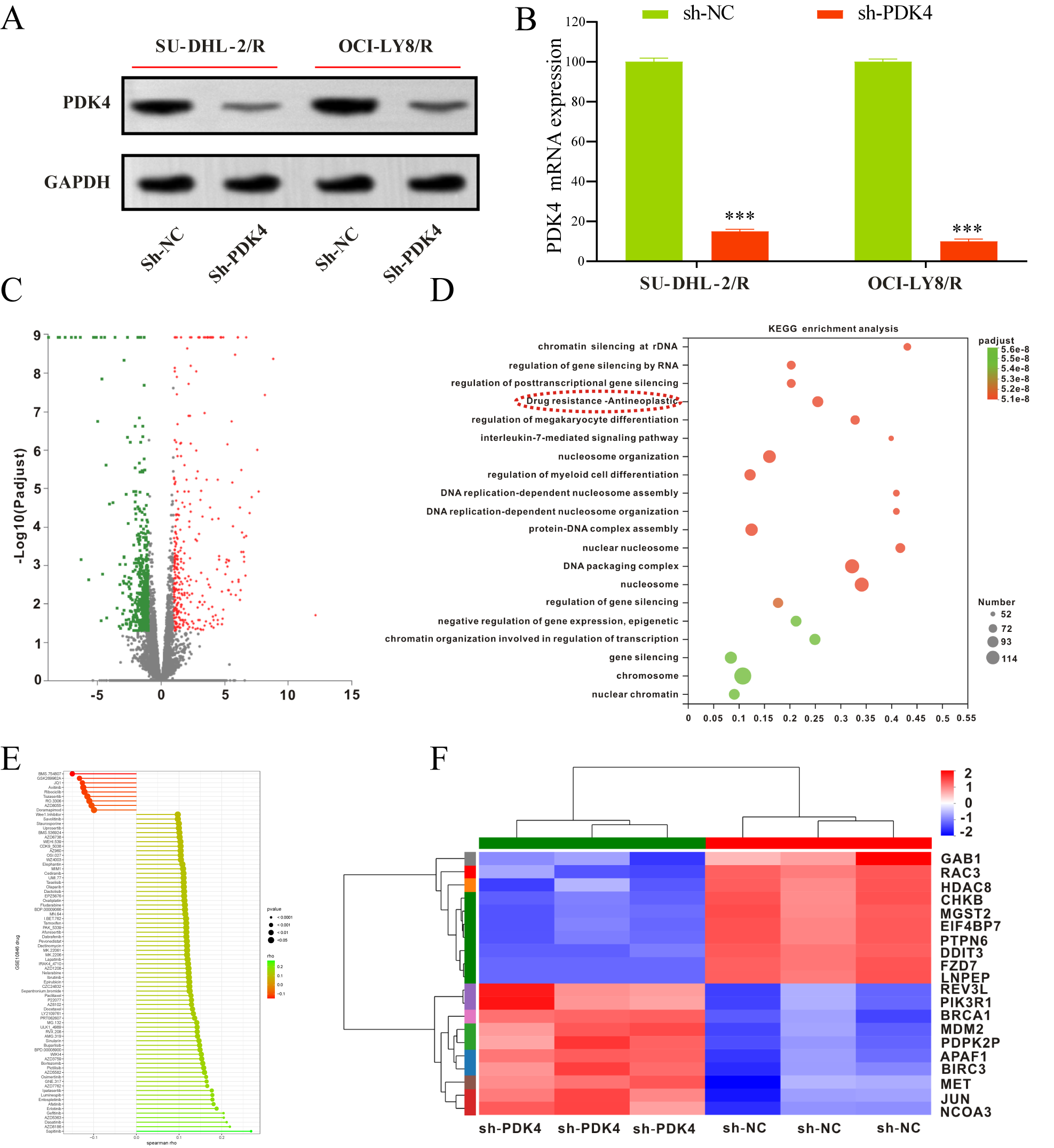


**Supplementary Figure 1. RNA-seq results in DLBCL-resistant cells comparing the sh-PDK4 group versus the sh-NC group.**

A. Western blot comparing PDK4 expression in PDK4-knockdown SU-DHL-2/R and OCI-LY8/R cells. B. Quantitative real-time PCR analysis comparing PDK4 expression in PDK4-knockdown SU-DHL-2/R and OCI-LY8/R cells. C. Differential gene expression analysis was performed using the Limma package in R. The results are displayed in a volcano plot, where significantly overexpressed genes are highlighted in red, and significantly underexpressed genes are shown in green. D. KEGG enrichment analysis was conducted using the clusterProfiler package in R, presenting the top 20 cellular functions differentiated by biological function. E. Drug sensitivity analysis was carried out using the oncopredict package in R, with Spearman’s analysis used to correlate PDK4 expression levels with the IC50 values of various drugs. F. Differential expression analysis was again performed using the Limma package in R, with the top 20 differentially expressed genes displayed in a heatmap.

## 2.2 Validation of Bioinformatics Results by qRT-PCR


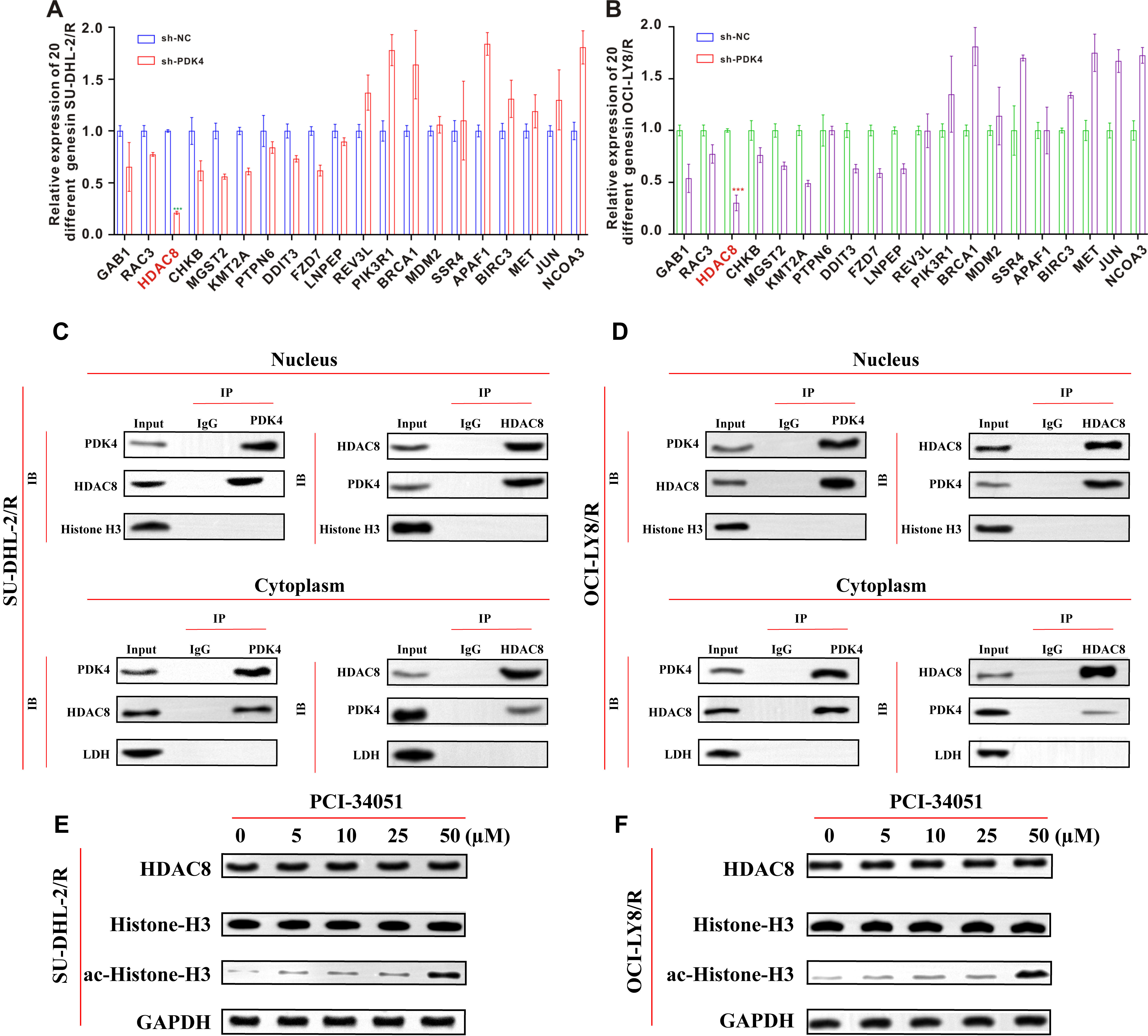


**Supplementary Figure 2. Interaction between HDAC8 and PDK4 and the inhibitory effect of PCI-34051.** A-B. qRT-PCR was utilized to measure the mRNA expression levels of 20 significantly different genes in two resistant cell types with sh-PDK4 knockdown. The results are shown in comparison to the sh-NC control group. (C) After nucleocytoplasmic separation of SU-DHL-2, PDK4 was immunoprecipitated in both nuclear and cytoplasmic fractions using an anti-PDK4 antibody, followed by detection of HDAC8 via western blotting. Control immunoprecipitation was performed using IgG. (D) After nucleocytoplasmic separation of OCI-LY8, PDK4 was immunoprecipitated in both nuclear and cytoplasmic fractions using an anti-PDK4 antibody, followed by detection of HDAC8 via western blotting. Control immunoprecipitation was performed using IgG. (E) HDAC8 expression and histone acetylation in SU-DHL-2/R cells treated with different concentrations of PCI-34051, as detected by WB. (F) HDAC8 expression and histone acetylation in OCI-LY8/R cells treated with different concentrations of PCI-34051, as detected by WB. Differences with *** indicate a highly significant difference with a P-value of less than 0.001.

## 2.3 Effect of Different Concentrations of Cyclophosphamide on the Viability of Bone Marrow Mesenchymal Stem Cells


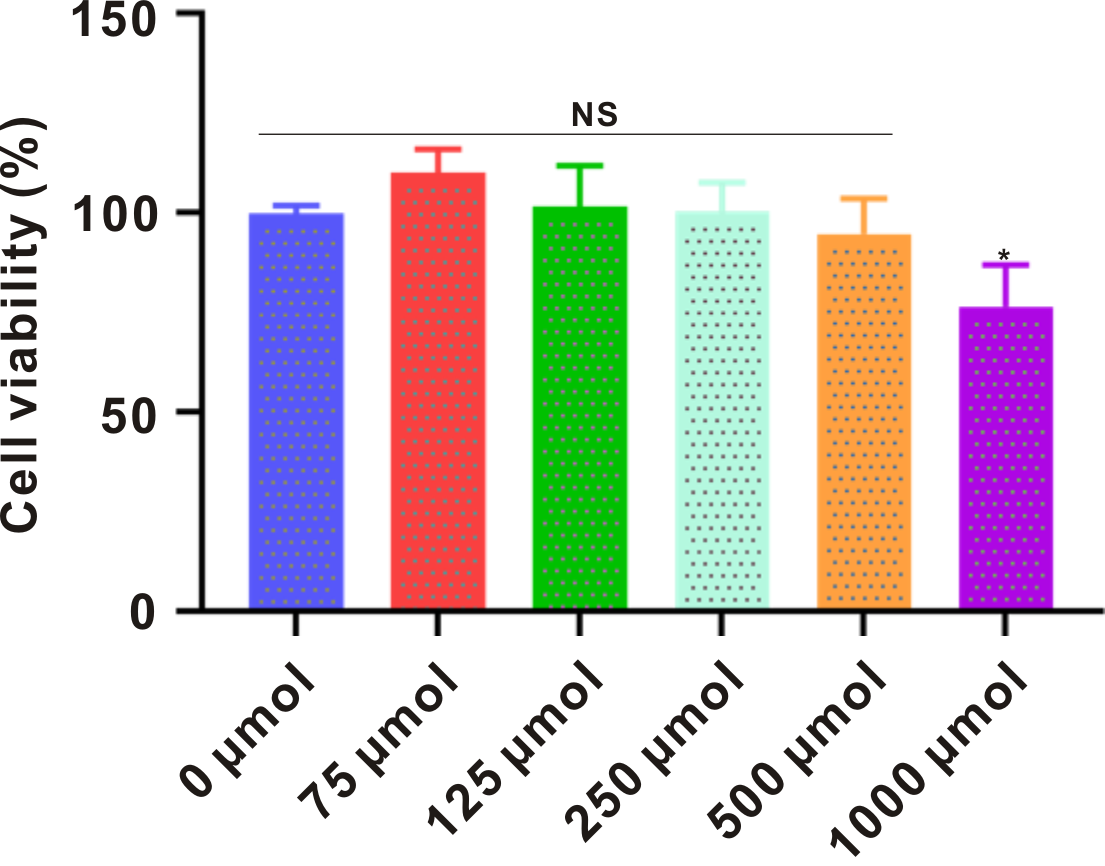


**Supplementary Figure 3.** Cell viability assay of BMSCs treated with various concentrations of cyclophosphamide (CTX) for 24 hours. The graph shows the cell viability percentages compared to the untreated control (0 μmol CTX). Significance indicators used are * for p<0.05, suggesting a statistically significant difference from the control, and NS (not necessary) for p>0.05, indicating no significant difference from the control.

## 2.4 Flow Cytometry Analysis of Exo^CTX^ Internalization


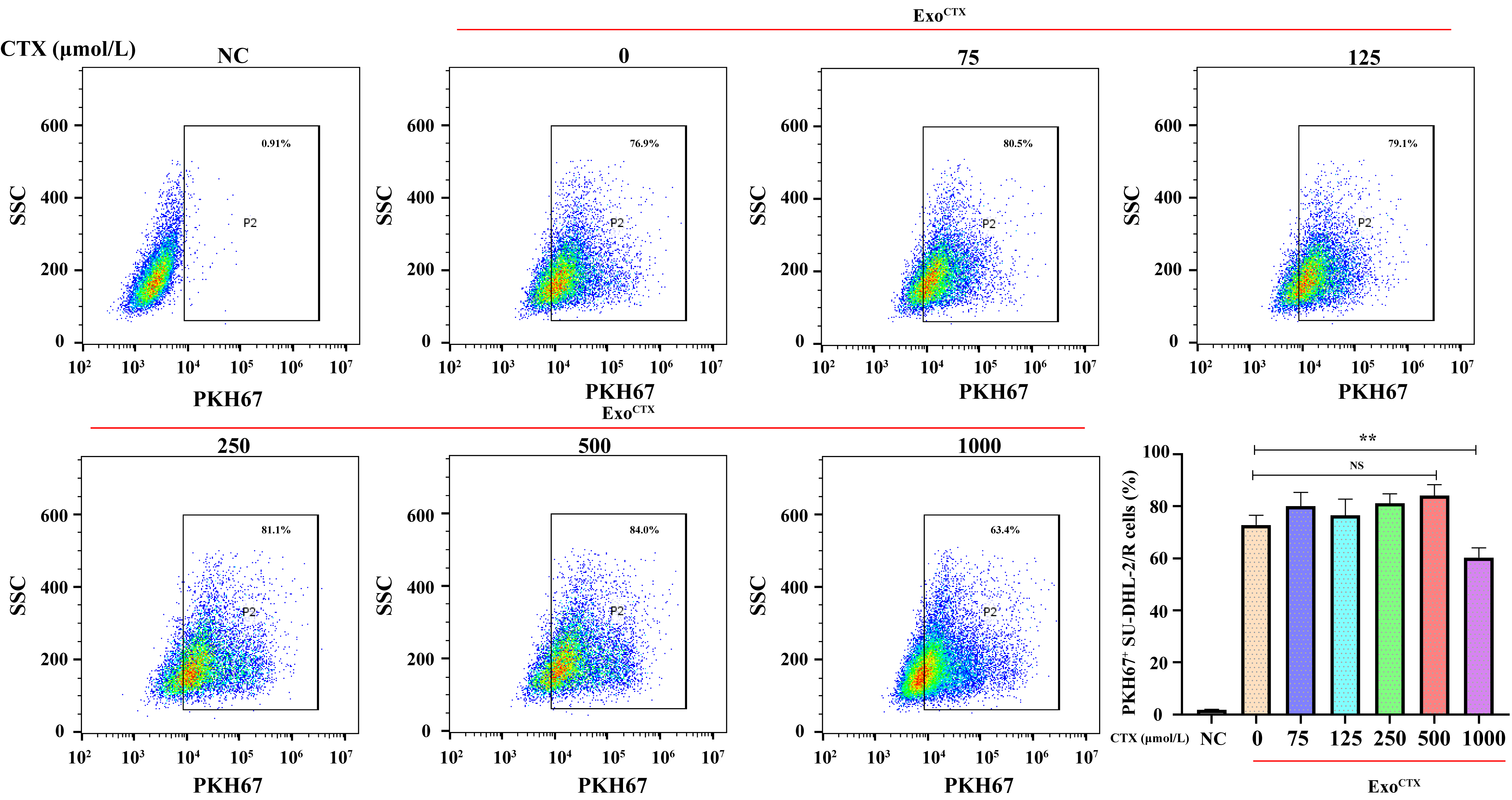


**Supplementary Figure 4.** Flow cytometric exosomes absorption assay of SU-DHL-2/R cells pre-cultured with various concentrations of CTX following PKH-67-labeled exosomes (5 μg/mL) incubation for another 12 h. All data are presented as means ±SEM. NS, no significant difference, ** P < 0.01.

## 2.5 Loading Efficiency and Degradation Resistance of Exo^CTX^ Loaded with siPDK4.


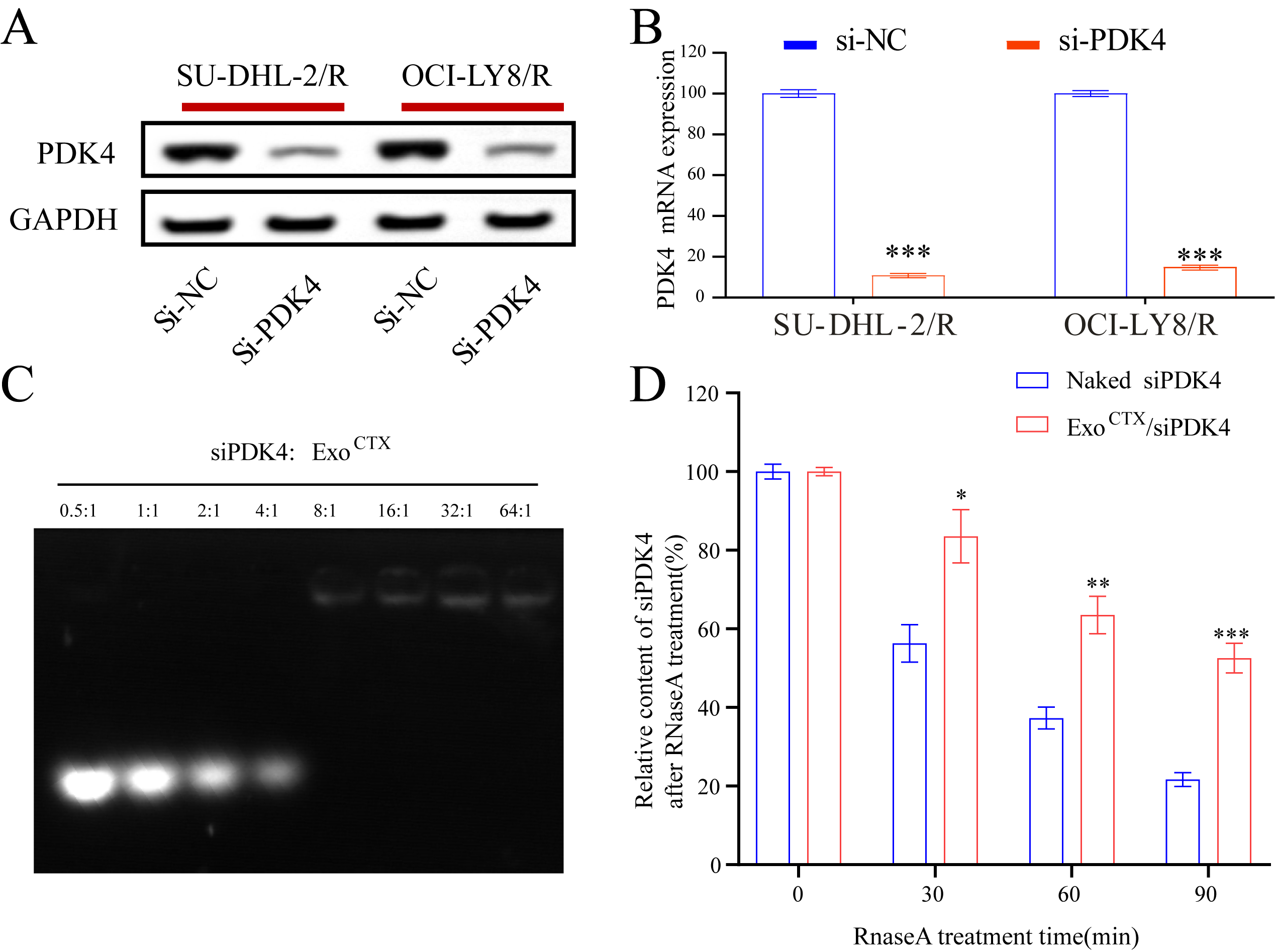


**Supplementary Figure 5.** (A). Western blot analysis compared the expression of PDK4 in Si-PDK4-treated SU-DHL-2/R and OCI-LY8/R cells. (B). Quantitative real-time PCR analysis compared the expression of PDK4 in Si-PDK4-treated SU-DHL-2/R and OCI-LY8/R cells. (C). Agarose gel electrophoresis is used to determine the optimal mass ratio for efficient siPDK4 loading onto ExoCTX；(D)The relative stability of siPDK4 encapsulated within ExoCTX compared to naked siPDK4 after treatment with RNase A is assessed using qRT-PCR. *p < 0.05, **p < 0.01, ***p < 0.001vs Naked siPDK4.

## 2.6 Flow Cytometry Analysis of Exo^CTX^/siPDK4 Transfection Efficiency


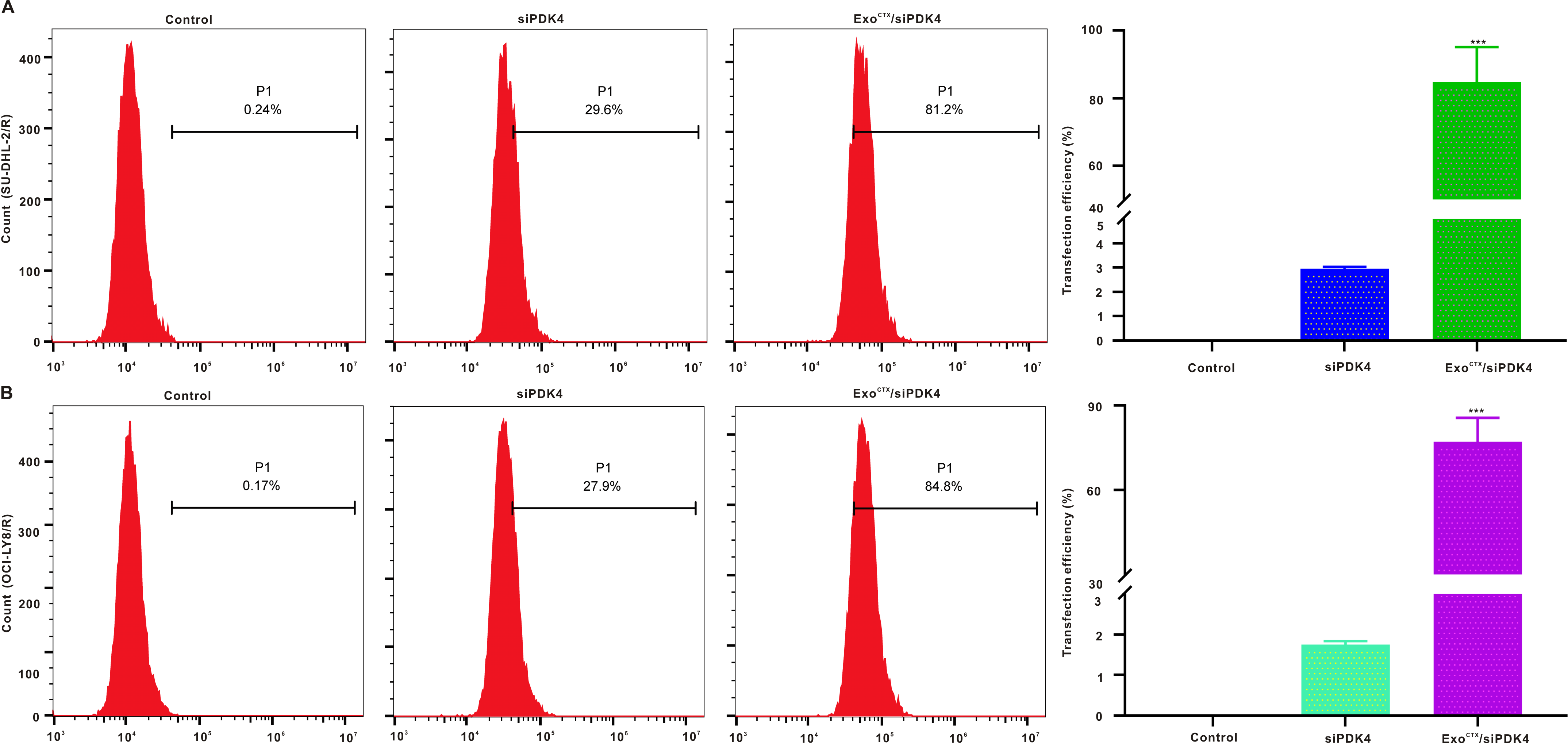


**Supplementary Figure 6.** Flow cytometry analysis of Exo^CTX^/siPDK4 stained with PKH67 in（A）The transfection efficiency in SU-DHL-2/R cells,（B）in OCI-LY8/R cells.***p < 0.001vs siPDK4。

## 2.7 Characterization Results of mAb(Rituximab)-NHS-PEG2000-Hyd-DSPE


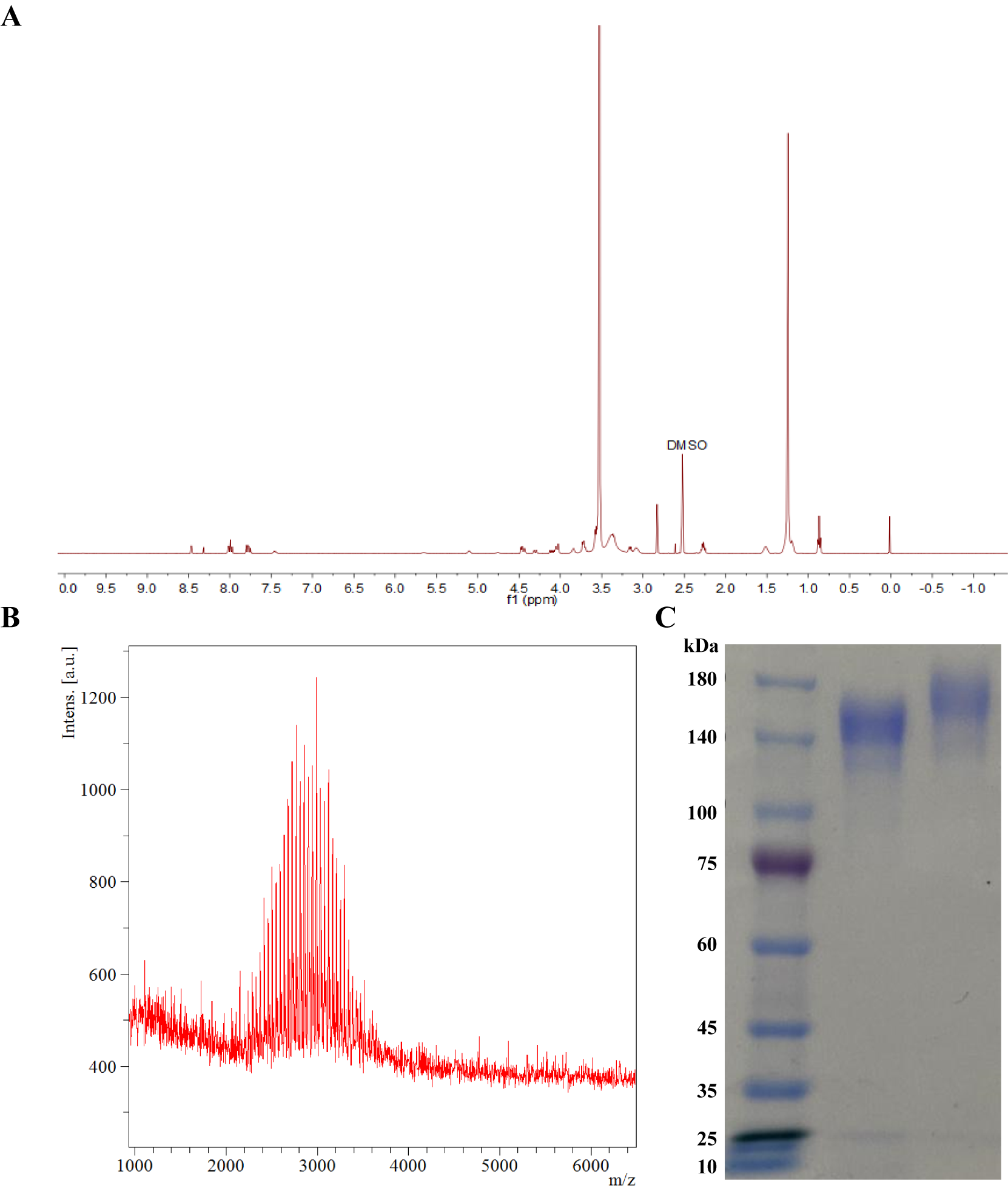


**Supplementary Figure 7.** (A) Nuclear magnetic resonance (NMR) results for DSPE-Hyd-PEG2000-NHS; (B) Mass spectrometry results for DSPE-Hyd-PEG2000-NHS; (C) Non-denaturing protein electrophoresis results for mAb(Rituximab)-NHS-PEG2000-Hyd-DSPE.

## 2.8 Fluorescence Spectroscopy Analysis of Cy5 Labeled on DSPE-Hyd-PEG2000-NHS-Rituximab


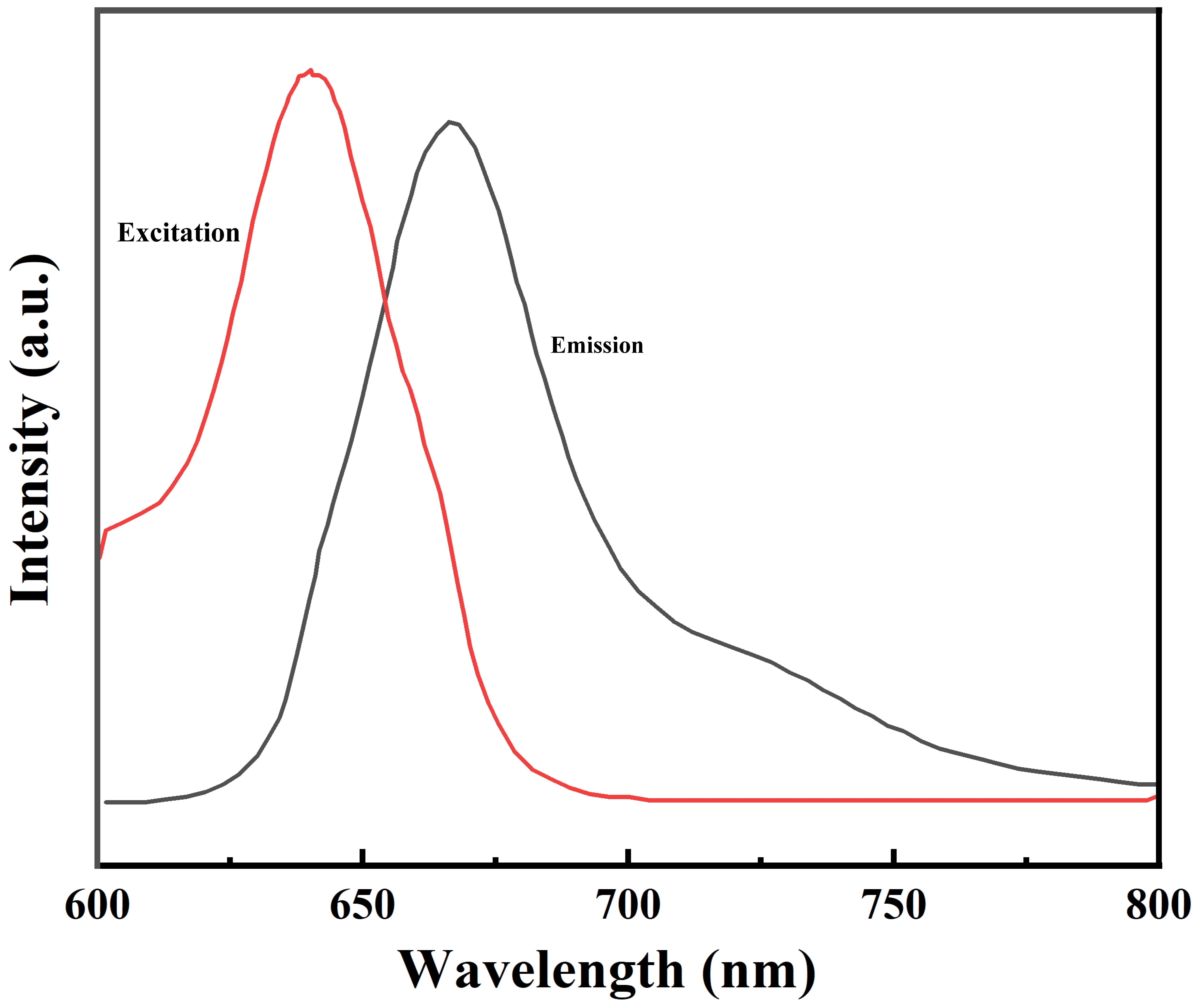


**Supplementary Figure 8.** Spectral analysis of excitation and emission wavelengths for DSPE-Hyd-PEG2000-NHS-Rituximab-Cy5.

## 2.9 In Vitro Release Characteristics of aCD20@Exo^CTX^/siPDK4 Nanoparticles


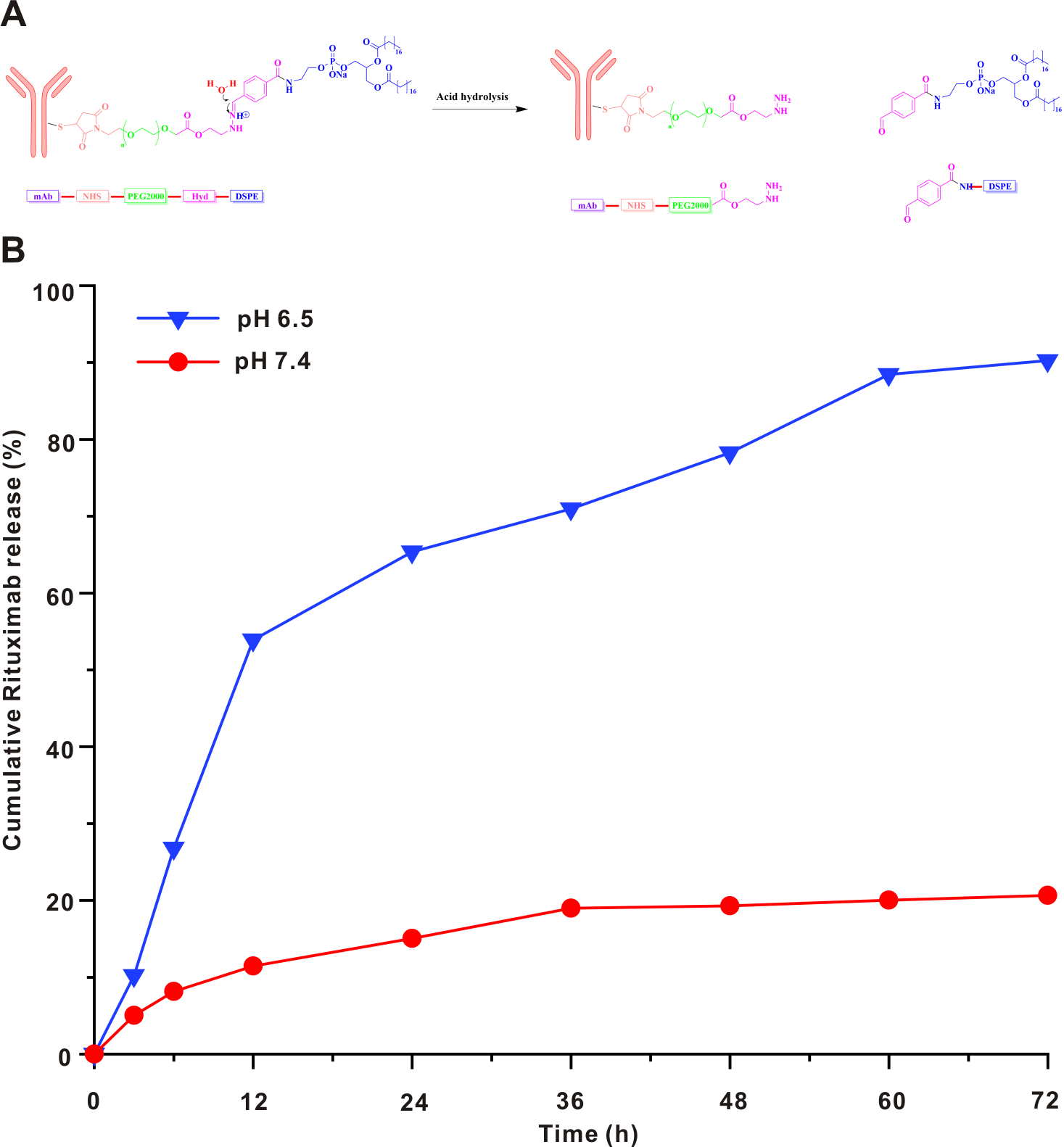


**Supplementary Figure 9.** (A) Schematic of aCD20 dissociation during the acidic disintegration of aCD20@Exo^CTX^/siPDK4 nanoparticles; (B) Release curves of rituximab from aCD20@Exo^CTX^/siPDK4 nanoparticles in PBS at pH 7.4 and pH 6.5. The data are presented as the mean values ± SD (n=3).

## 2.10 In Vitro Hemocompatibility, Stability, and Safety Assessment of aCD20@Exo^CTX^/siPDK4 Nanoparticles


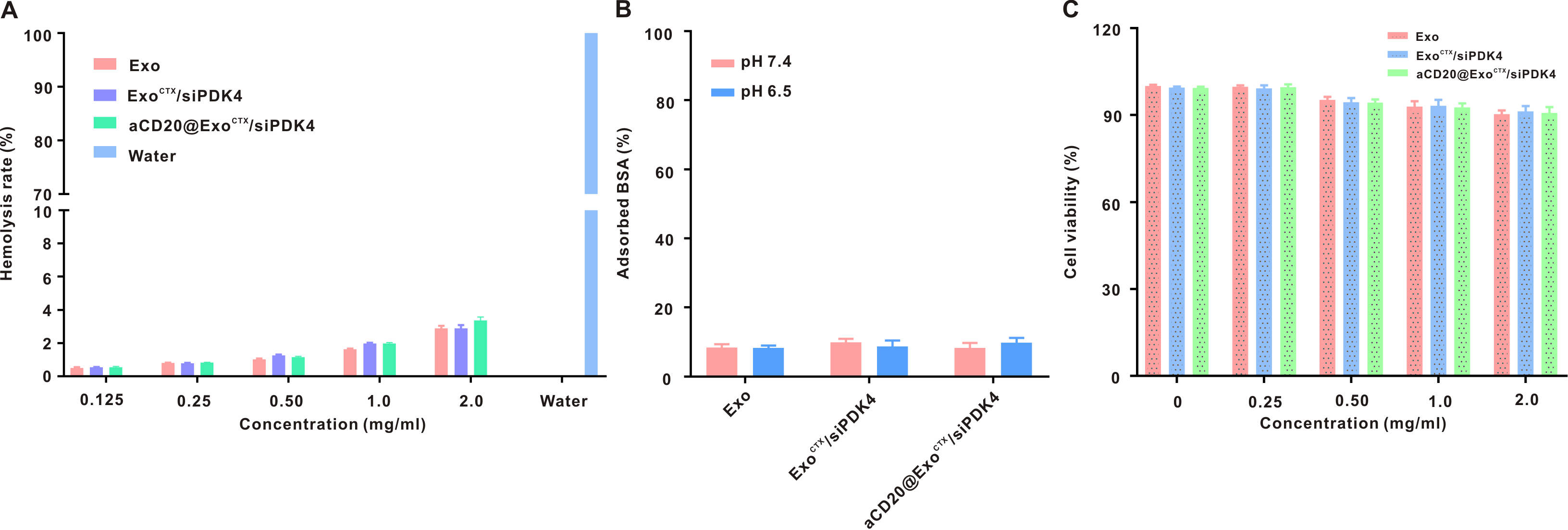


**Supplementary Figure 10.** (A) Hemolysis rate of nanoparticles at various concentrations. (B) Protein adsorption rate of nanoparticles at pH 6.5 and pH 7.4. (C)B cell viability was treated with multiple Exo, Exo^CTX^/siPDK4 concentrations, and aCD20@Exo^CTX^/siPDK4 for 24 h. Data are presented as the means ± SD (n = 3).

## 2.11 In Vitro Induction of Apoptosis in SU-DHL-2 and SU-DHL-2/R Cells by aCD20@Exo^CTX^/siPDK4


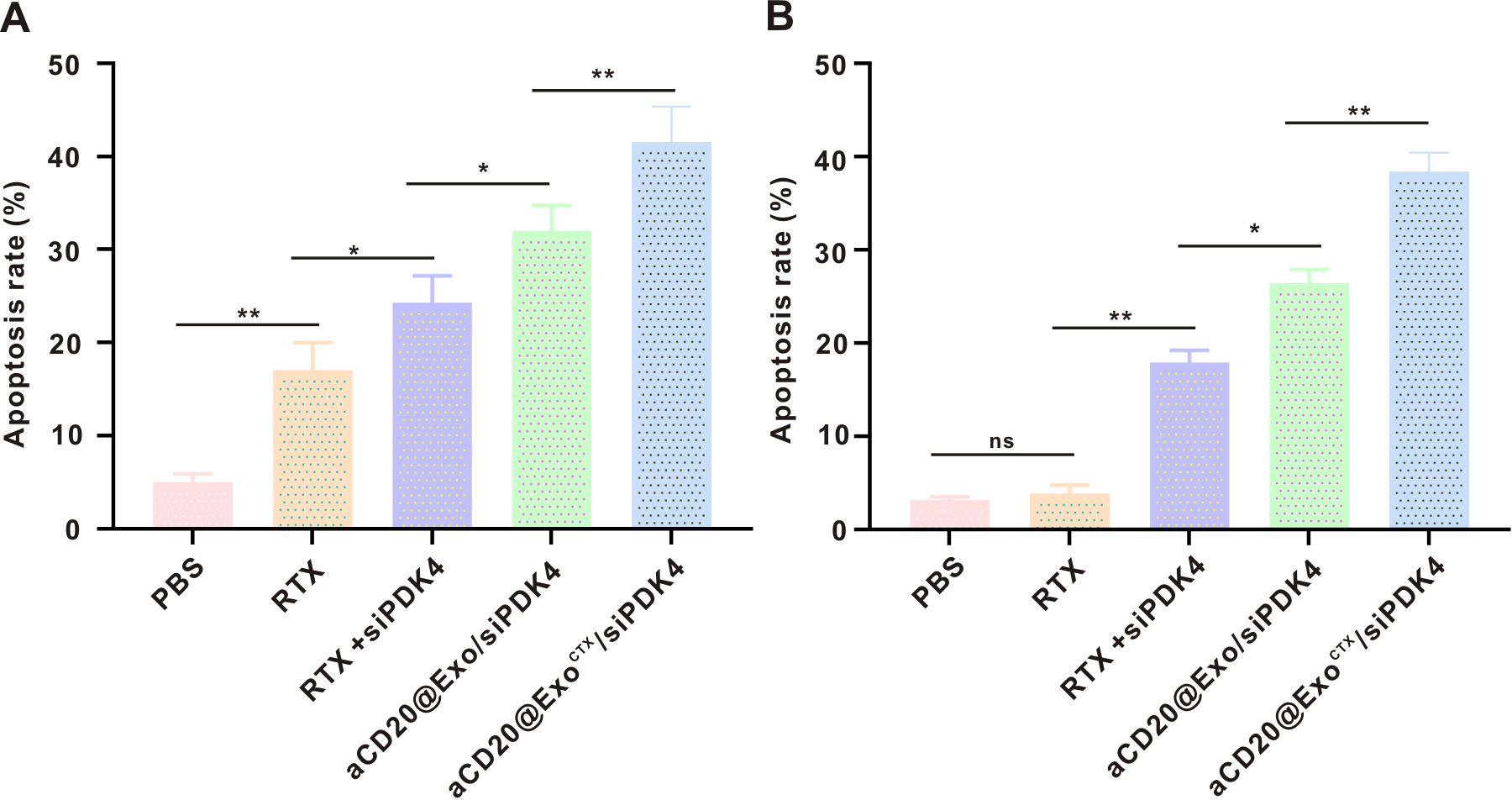


**Supplementary Figure 11 (A-B).** Flow cytometry was used to identify the apoptosis rate of SU-DHL-2 and SU-DHL-2/R cells after 24 hours. Data are expressed as the average ± SD(n = 3). *p < 0.05, **p < 0.01.

## 2.12 In Vitro Expression of Bcl-2, Cleaved PARP, Cleaved Caspase-3, PDK4, HDAC8, Phosphorylated HDAC8, and CD20 Proteins Induced by aCD20@Exo^CTX^/siPDK4


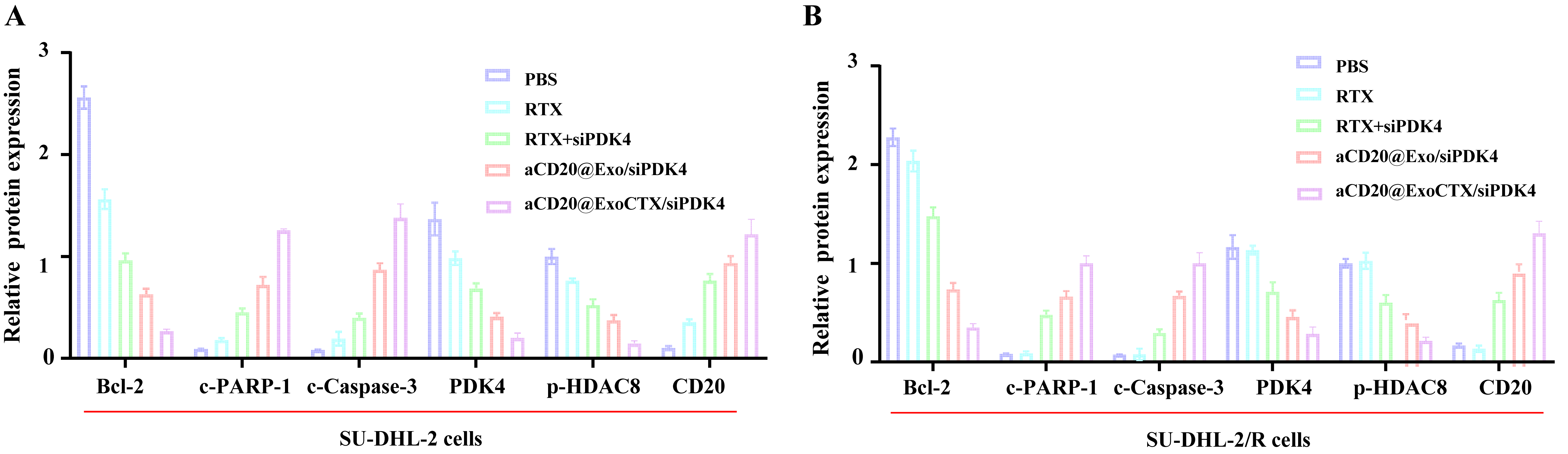


**Supplementary Figure 12 (A-B).** The quantitation data from the immunoblotting analysis were analyzed via the ImageJ program, which was expressed as average ± SD (n = 3).

## 2.13 Quality control analysis for the Diffuse sizeable B-cell lymphoma single-cell RNA-seq database.


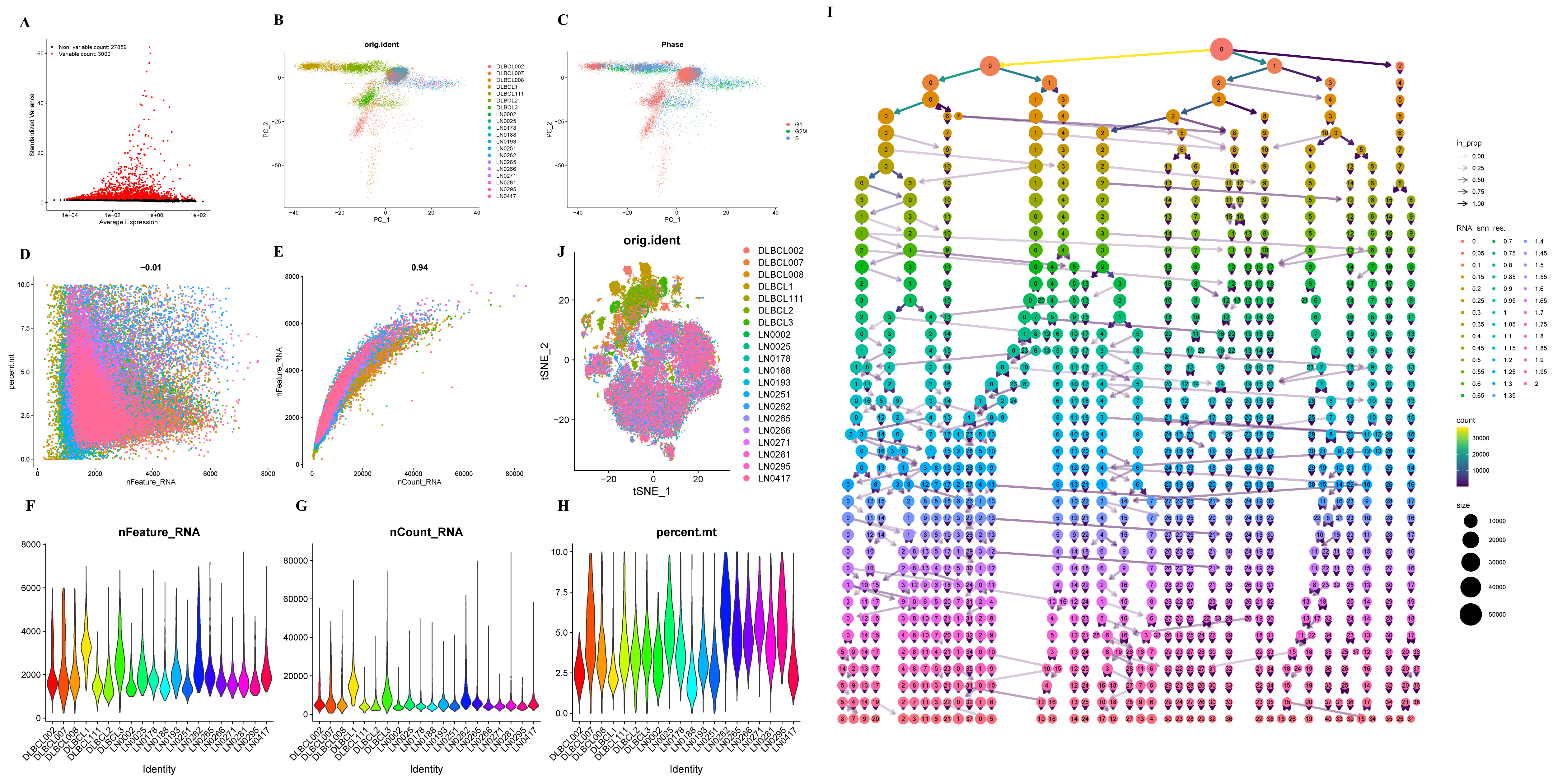


**Supplementary Figure 13.** Quality control analysis for the Diffuse sizeable B-cell lymphoma single database. (A) Identification of genes with high variability across cells; the x-axis represents average expression, and the y-axis denotes normalized variance. (B) PCA representation is colored according to 20 individual samples. (C) PCA depiction of cell cycle distribution across the 20 samples. (D) Scatter plot correlating overall gene expression in cells with the proportion of mitochondrial genes. (E) Scatter plot correlating nfeature with ncount. (F-H) Violin plots illustrating gene counts， total gene expression levels， and the proportion of mitochondrial genes, respectively, for each sample. (I) Display of cell clustering at various resolutions. (J) t-SNE plot showing the detailed Seurat clusters.

## 2.14 Identification of major cell types and malignant B cells and their corresponding subpopulations analysis.


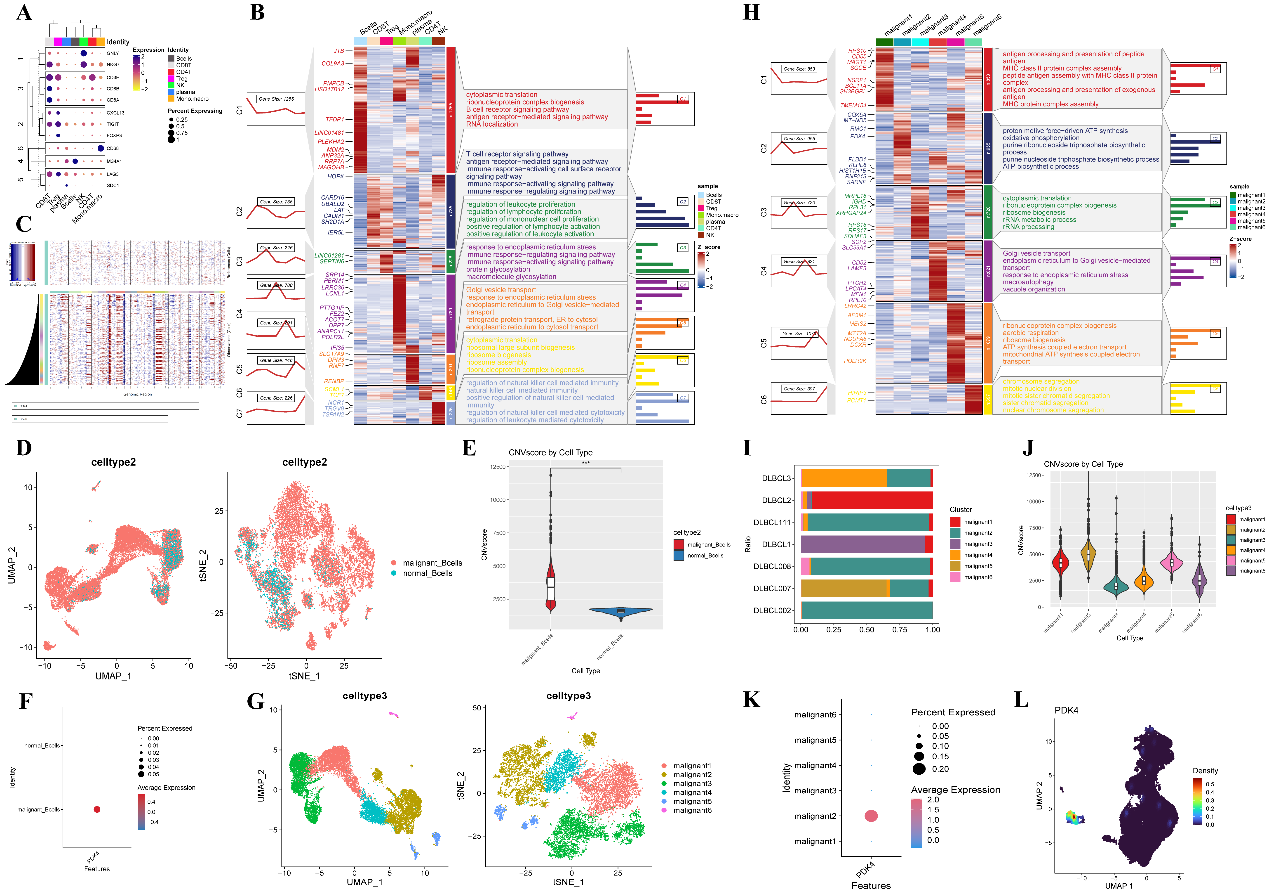


**Supplementary Figure 14. Identification of major cell types and malignant B cells and their corresponding subpopulations analysis.** (A) Gene expression profiles for each cell cluster, with dot size indicating the proportion of cells expressing a specific marker gene and color intensity reflecting average gene expression level. (B) Heatmap showing differential expression of genes (DEGs) in cell subgroups. (C) Hierarchical heatmap showing large-scale copy number variations in B cells. The control is CD4+ T cells. (D) t-SNE and UMAP plots showing malignant and normal B cells. (E) Box plot showing CNV scores of malignant and normal B cells. (F) Feature plot showing PDK4 expression in malignant and normal B cells. (G) t-SNE and UMAP plots showing subgroups of malignant B cells. (H) Hierarchical heatmap displaying differential genes and functions in different malignant B cell subgroups. (I) Proportions of malignant B cell subgroups in different samples. (J) CNV scores of different malignant B cell subgroups. (K) Feature plot showing PDK4 expression among malignant B cell subgroups. (L) The density plot showing the expression of PDK4.

## 2.15 PDK4's role in reshaping the tumor microenvironment (TME) in DLBCL.


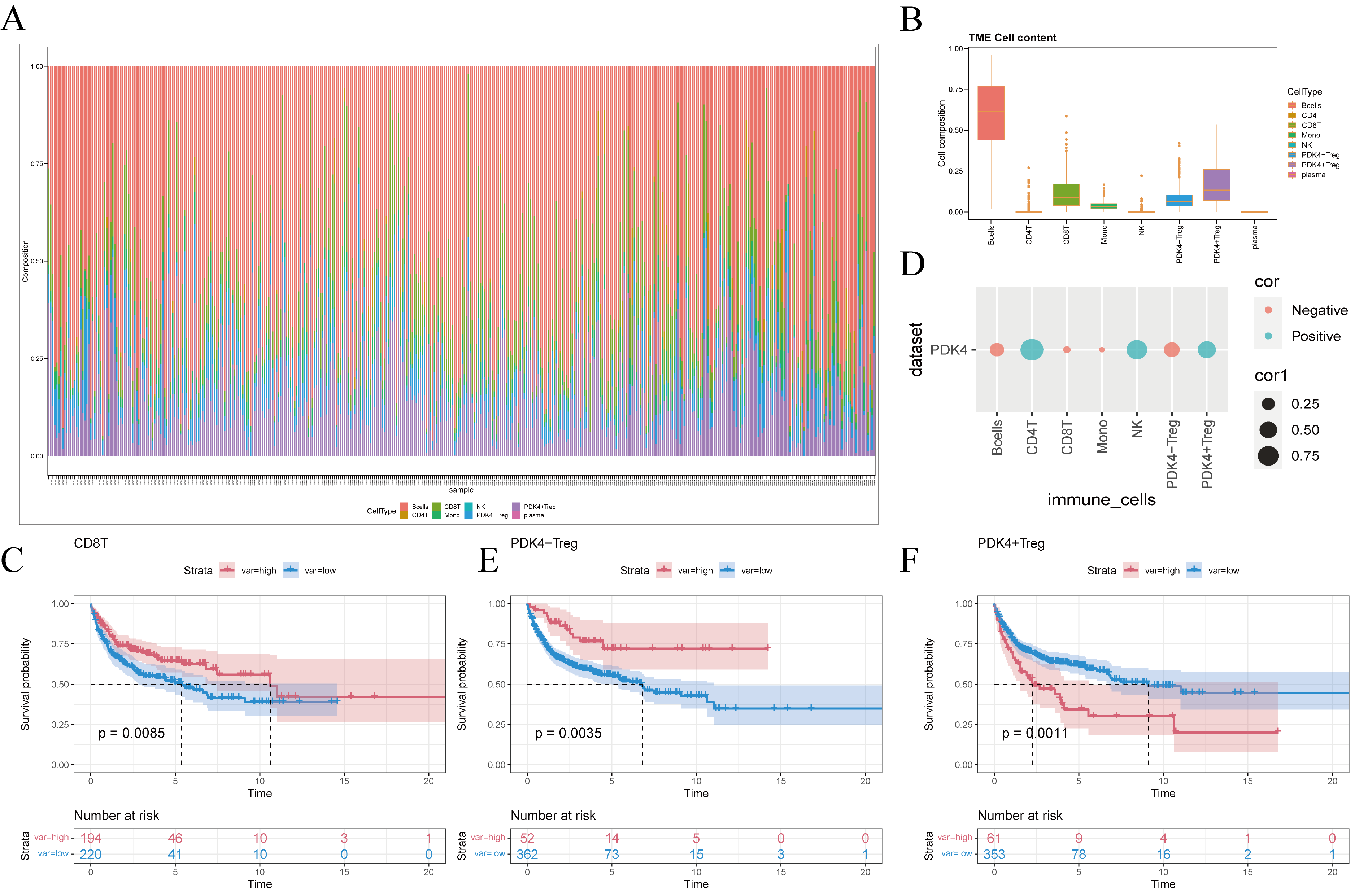


**Supplementary Figure 15.** PDK4's role in reshaping the tumor microenvironment (TME) in DLBCL. (A) Assessment of immune infiltration per sample. (B) Analysis of cellular composition proportions in samples. (C) Survival prognosis analysis for DLBCL patients with high and low proportions of CD8^+^ T cells. (D) Correlation study between PDK4 and cells. (E) Survival prognosis analysis for DLBCL patients with high and low proportions of PDK4^-^ Treg. (F) Survival prognosis analysis for DLBCL patients with high and low proportions of PDK4^+^ Treg.

## 2.16 PDK4^+^ Tregs Play a Crucial and Efficient Role in Cellular Communication


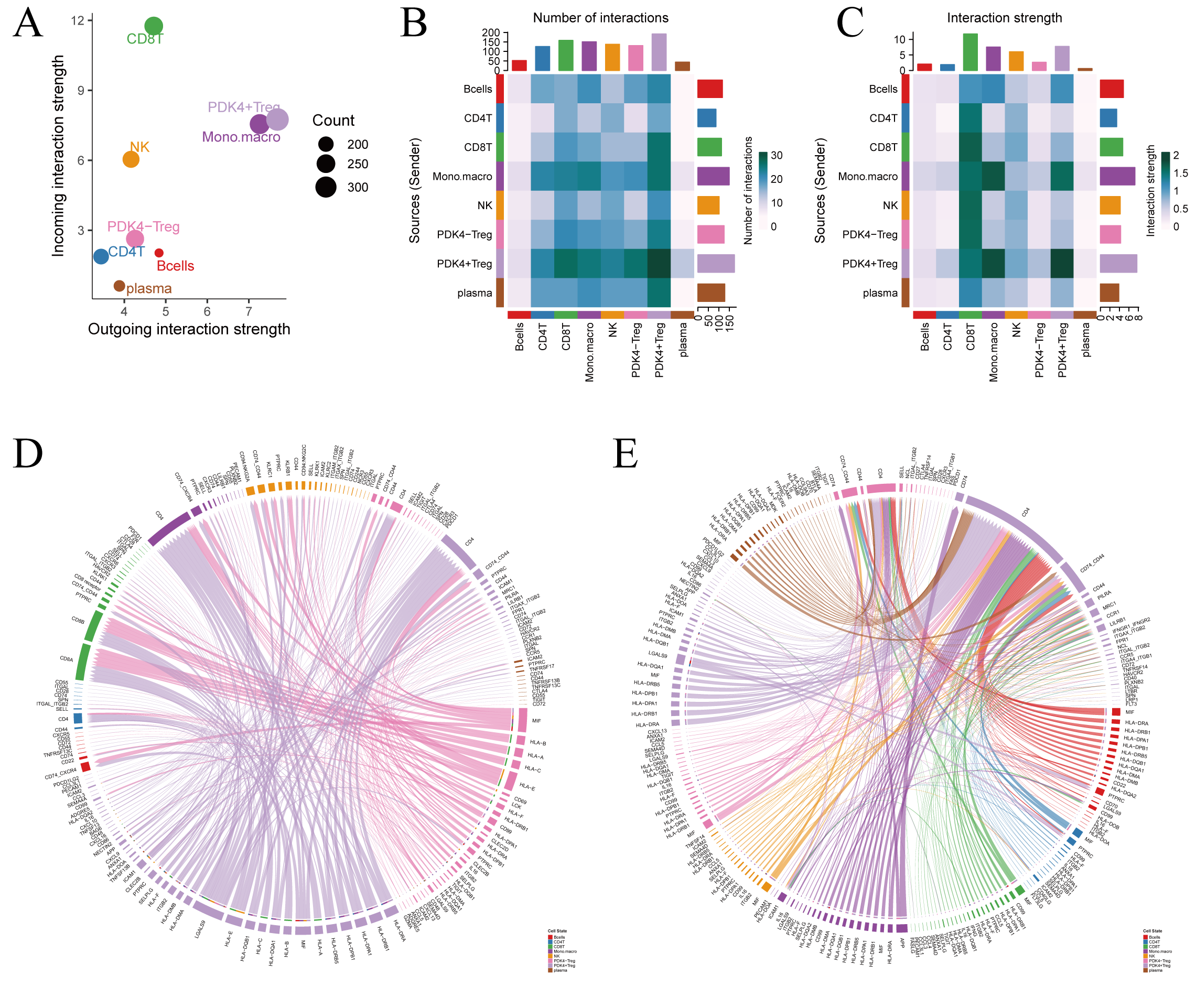


**Supplementary Figure 16.** (A) 2D representation of primary communication sender and receivers. (B) Heatmap showing the number of cellular communications with all cell subtypes acting as senders and receivers. (C) Heatmap displaying the intensity of cellular communications with all cell subtypes acting as senders and receivers. (D) Chord diagram illustrating the influence of PDK4^+^Treg and PDK4^-^Treg acting as senders on other cell subpopulations. (E) Chord diagram illustrating the influence of PDK4^+^Treg and PDK4^-^Treg acting as receivers on other cell subpopulations.

## 2.17 Significant Impact of PDK4 on the Biological Functions of Tregs


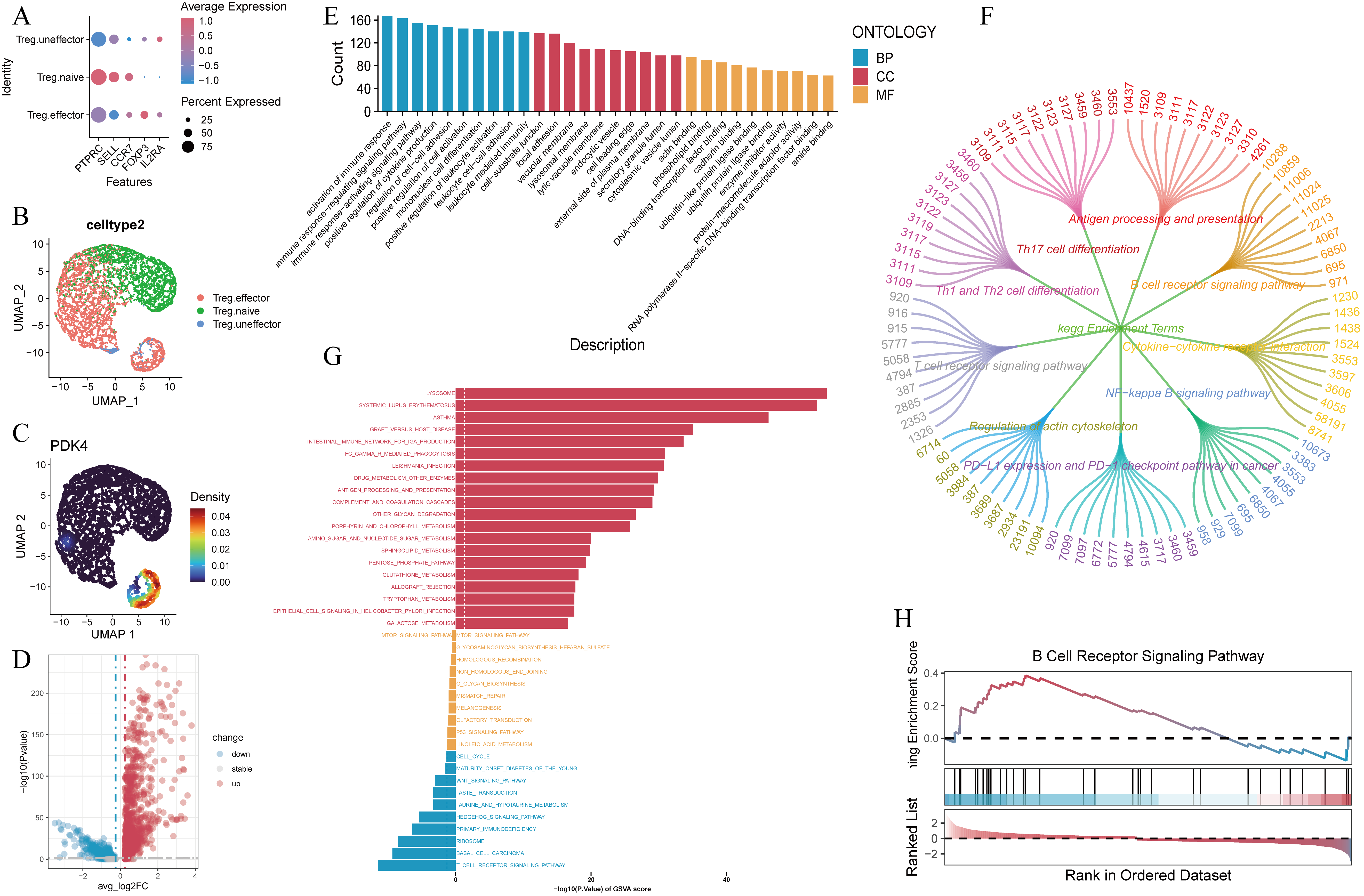


**Supplementary Figure 17.** (A) Dot plot showing classical marker genes of Treg cell subtypes. (B) UMAP plot illustrating different cell subtypes. (C) UMAP plot displaying PDK4 expression levels (colors corresponding to different expression levels). (D) Volcano plot contrasting genes with varying PDK4 expressions. (E) Gene Ontology (GO) analysis of differentially expressed genes. (F) Gene Set Variation Analysis (GSVA) of differential genes. (G) Kyoto Encyclopedia of Genes and Genomes (KEGG) analysis of differential genes. (H) Gene Set Enrichment Analysis (GSEA) of differential genes.

## 2.18 PDK4 Promotes the Differentiation of Tregs into More Suppressive Subtypes


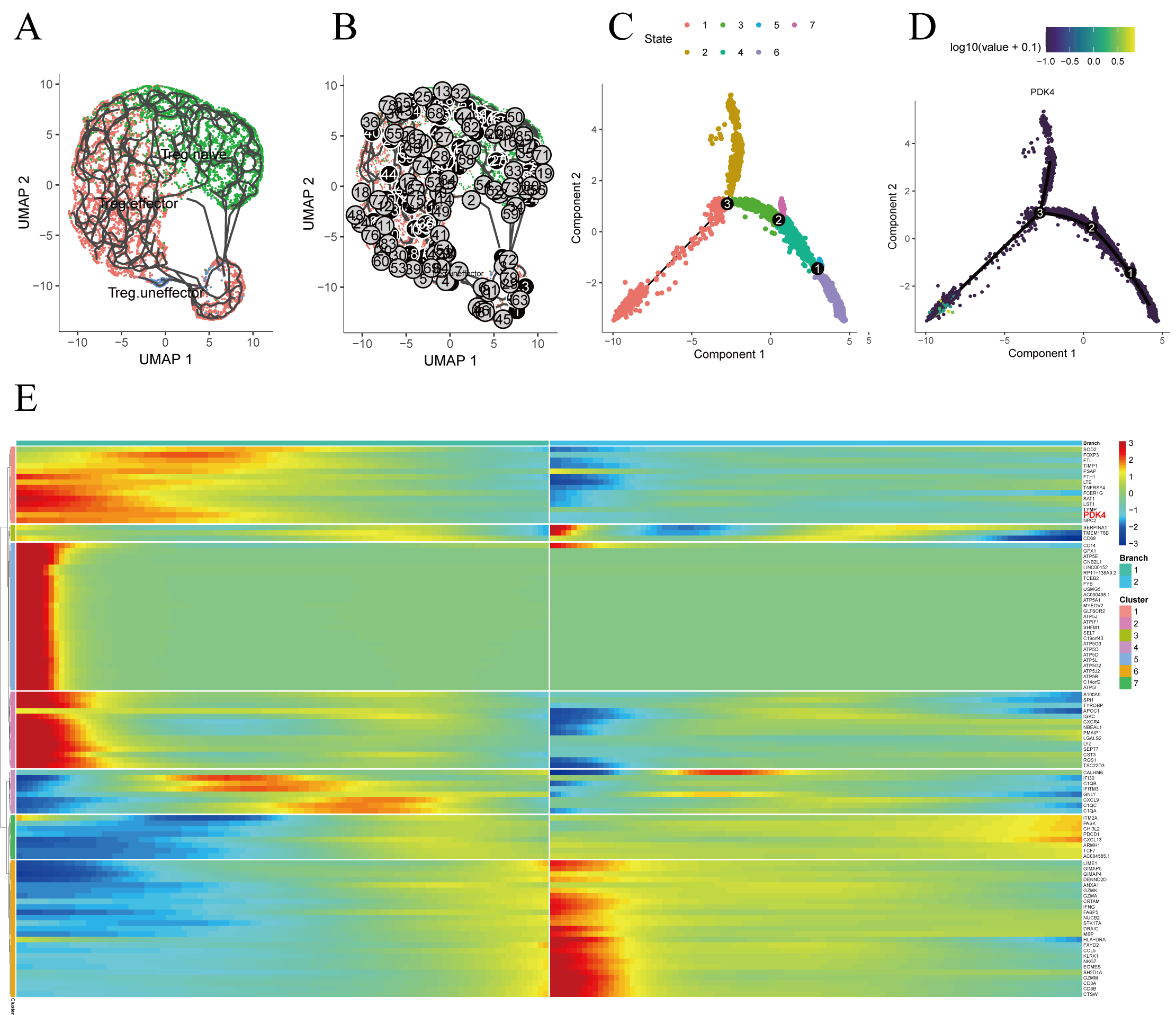


**Supplementary Figure 18.** (A-B) Monocle 3 trajectory plots depicting Treg subcluster differentiation paths: (A) graph and cell type, (B) graph and cell type. (C-D) Monocle 2 trajectory plots of Treg subcluster dynamics: (C) state (D) PDK4 expression. (E) Heatmap showing the trend of expression changes of time-varying related genes in two branches.

## 2.19 Statistical Data on In Vitro CRT Expression, DC Maturation, and CD8+ T Cell Expression


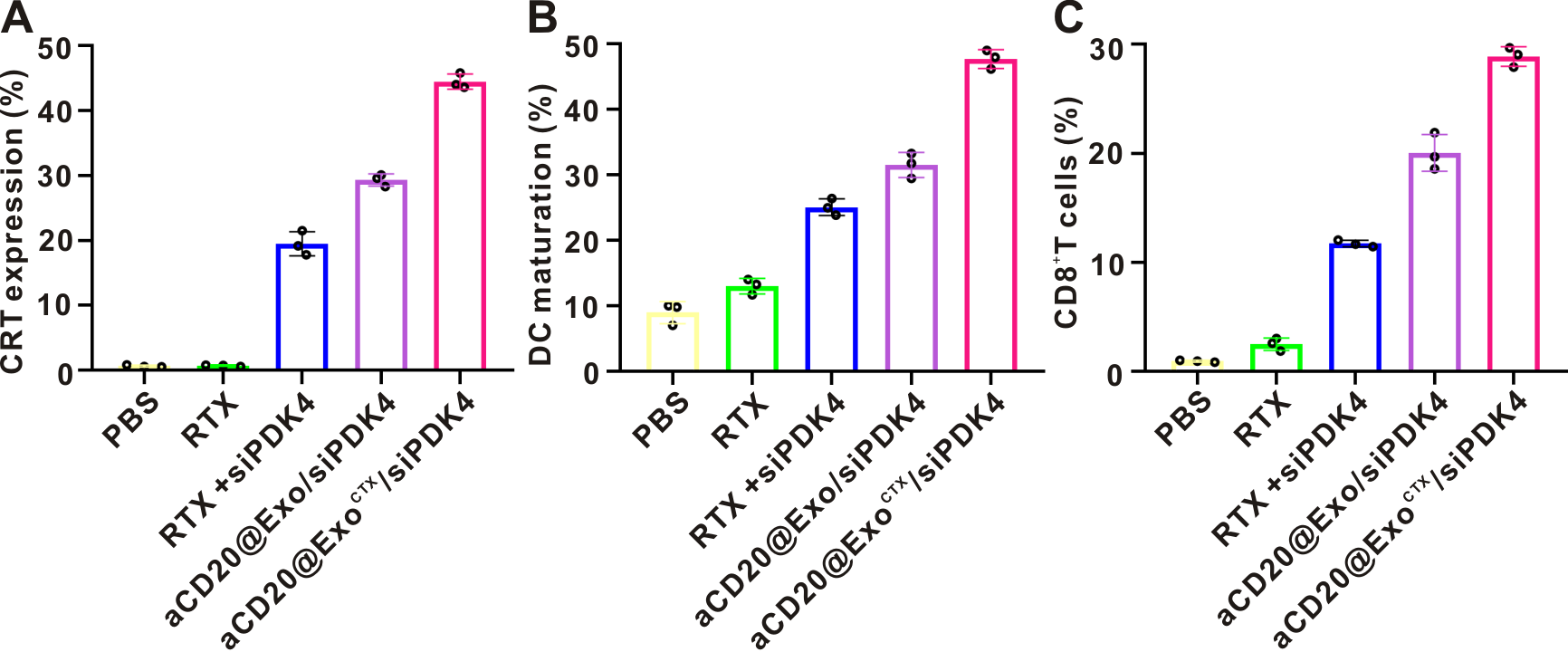


**Supplementary Figure 19 (A-C).** Statistical analysis of CRT expression (A), DC cell maturation (B), and CD8^+^ T cells producing (C) *in vitro.* Data are expressed as the average ± SD (n = 3).

## 2.20 Scheme for the generation of the humanized mouse model


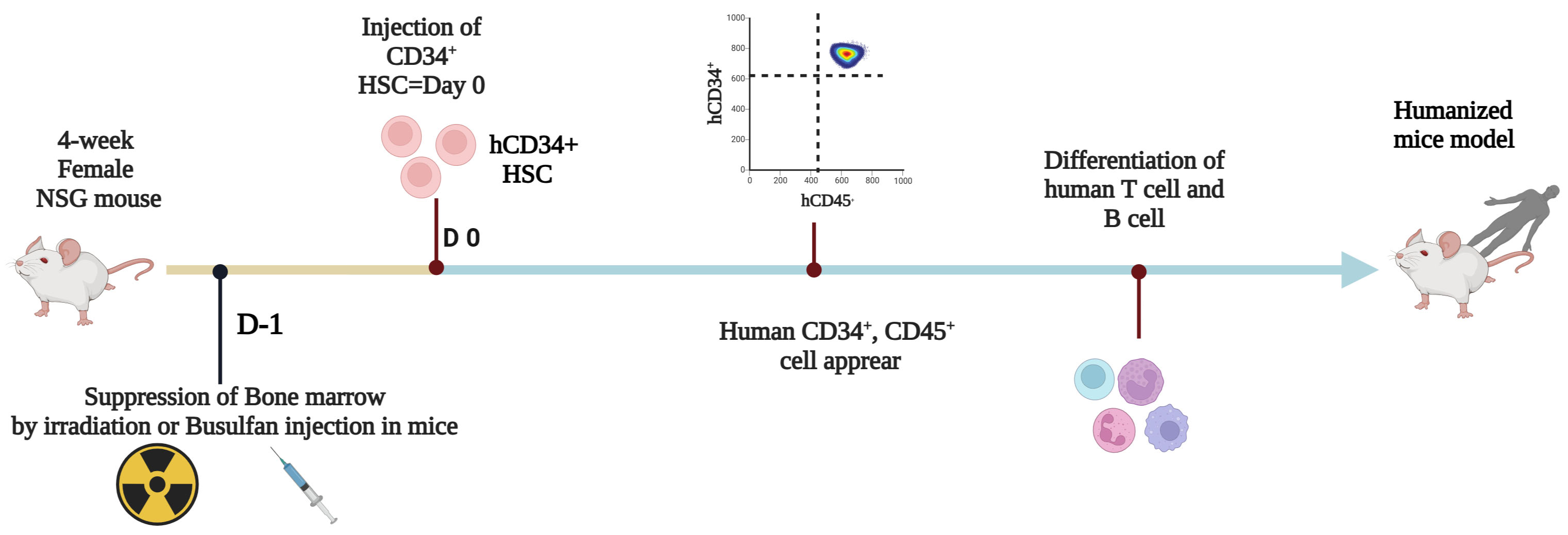


**Supplementary Figure 20.** 4-week-old female NOD-scid IL2rγnull (NSG) mice were prepared to generate humanized mice.

## 2.21 Immunofluorescence Detection of Apoptosis Protein Expression Induced by aCD20@Exo^CTX^/siPDK4 In Vivo


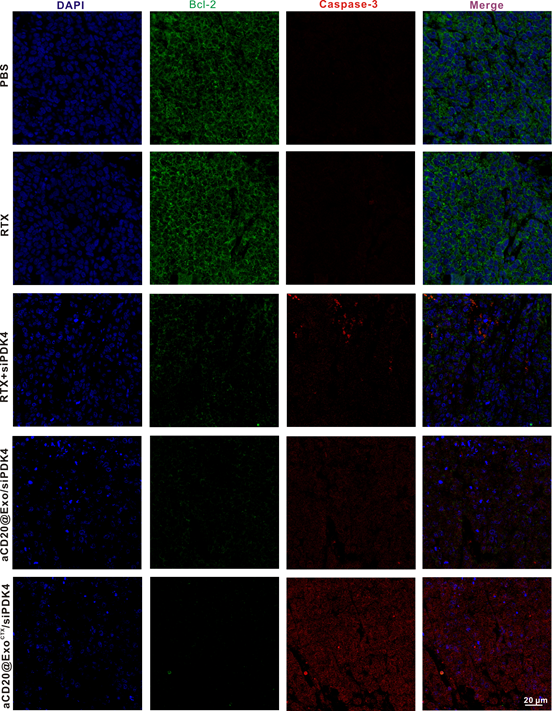


**Supplementary Figure 21.** The animals were under the treatment of PBS、RTX、RTX+siPDK4、aCD20@Exo/siPDK4 and aCD20@Exo^CTX^/siPDK4, respectively, and Bcl-2 (green), and Caspase-3 (red) in the tumor tissues were analyzed by immunofluorescence staining—scale bar: 20 μm.

## 2.22 Statistical Data on In Vivo DC Maturation, Expression of CD3^+^ T Cells, CD8^+^ T Cells, and Treg Cells


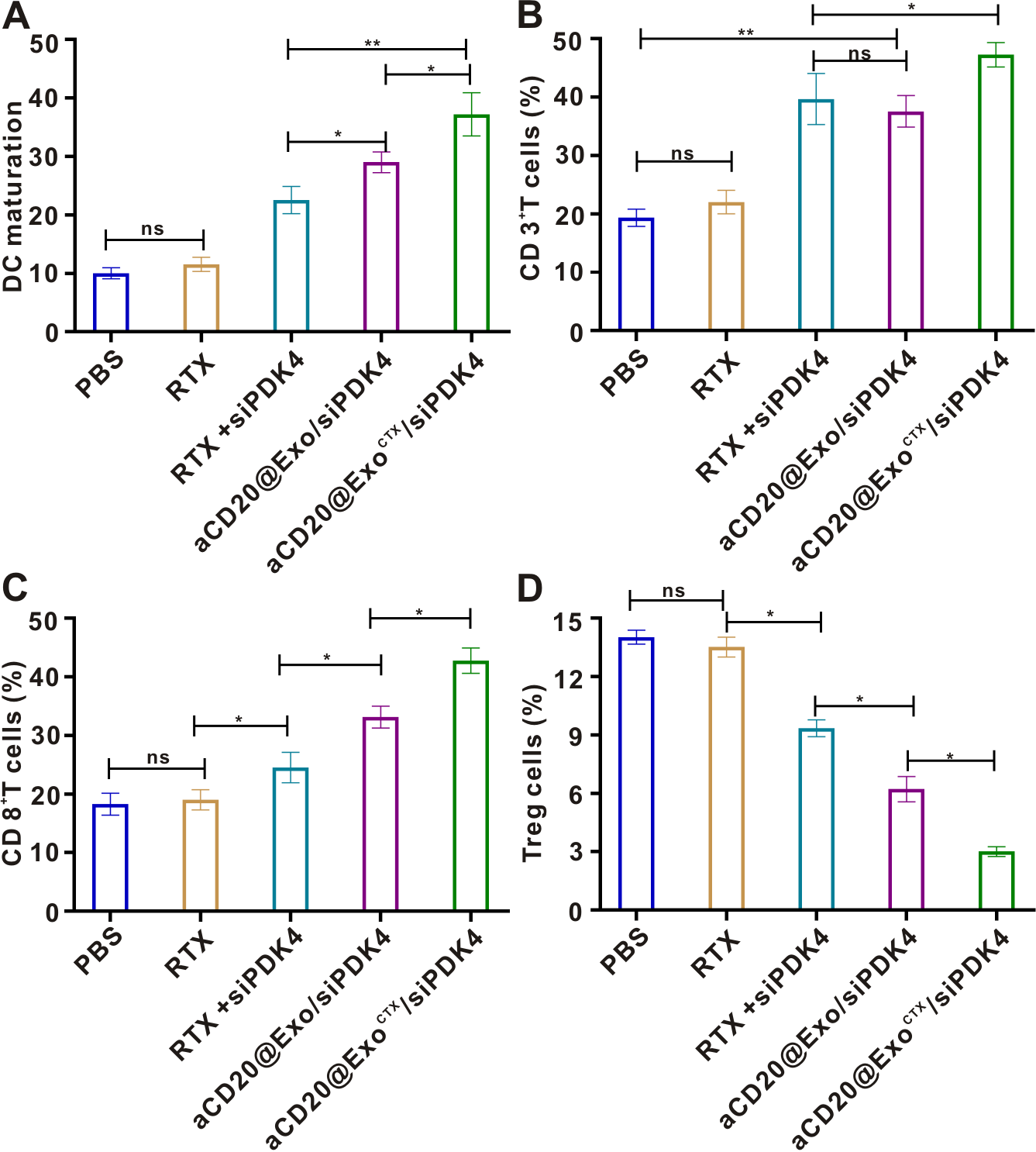


**Supplementary Figure 22 (A-D).** Statistical analysis of DC cell maturation (A), CD3^+^T (B), CD8^+^ T (C), and Treg cells producing (D) *in vivo*. Data are expressed as the average ± SD (n = 6). *p < 0.05, **p < 0.01. ns, no significant difference.

## 2.23 Proliferation, Differentiation, Maturation, or Activation of Immune Cells Induced by BMSC-Derived Exosomes


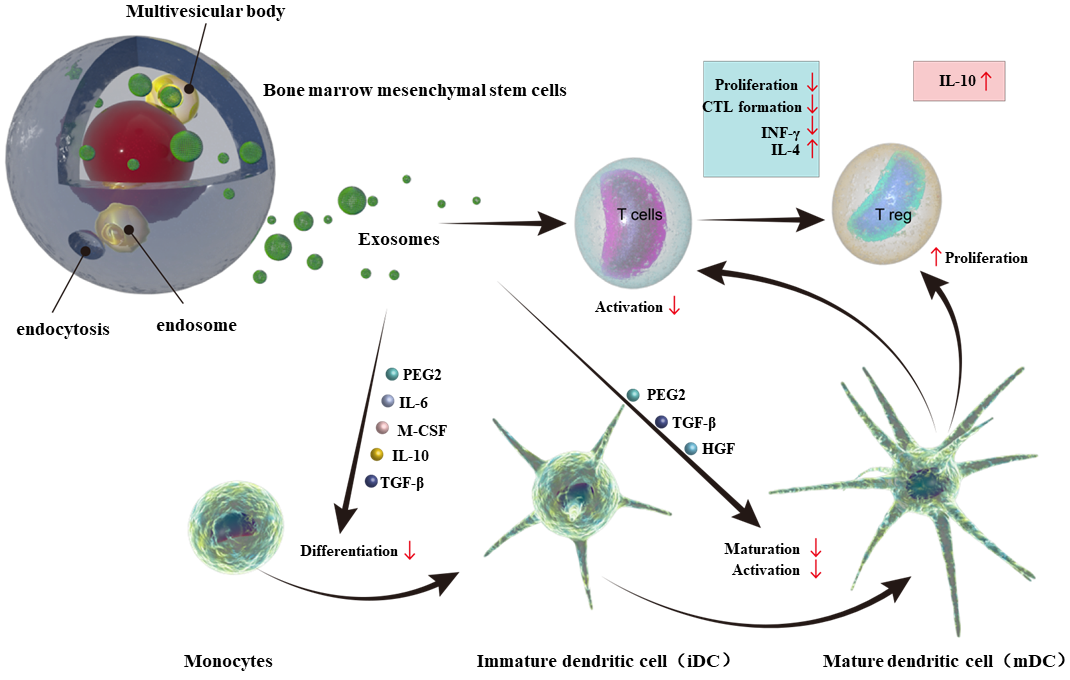


**Supplementary Figure 23.** Exosomes derived from BMSCs inhibit T cell activation and dendritic cell maturation and promote the proliferation of regulatory T cells.

## 2.24 Flow Cytometry Analysis of the Proportion of Tregs (CD4^+^CD25^+^Foxp3^+^) in Healthy Human Controls and Human DLBCL Patients


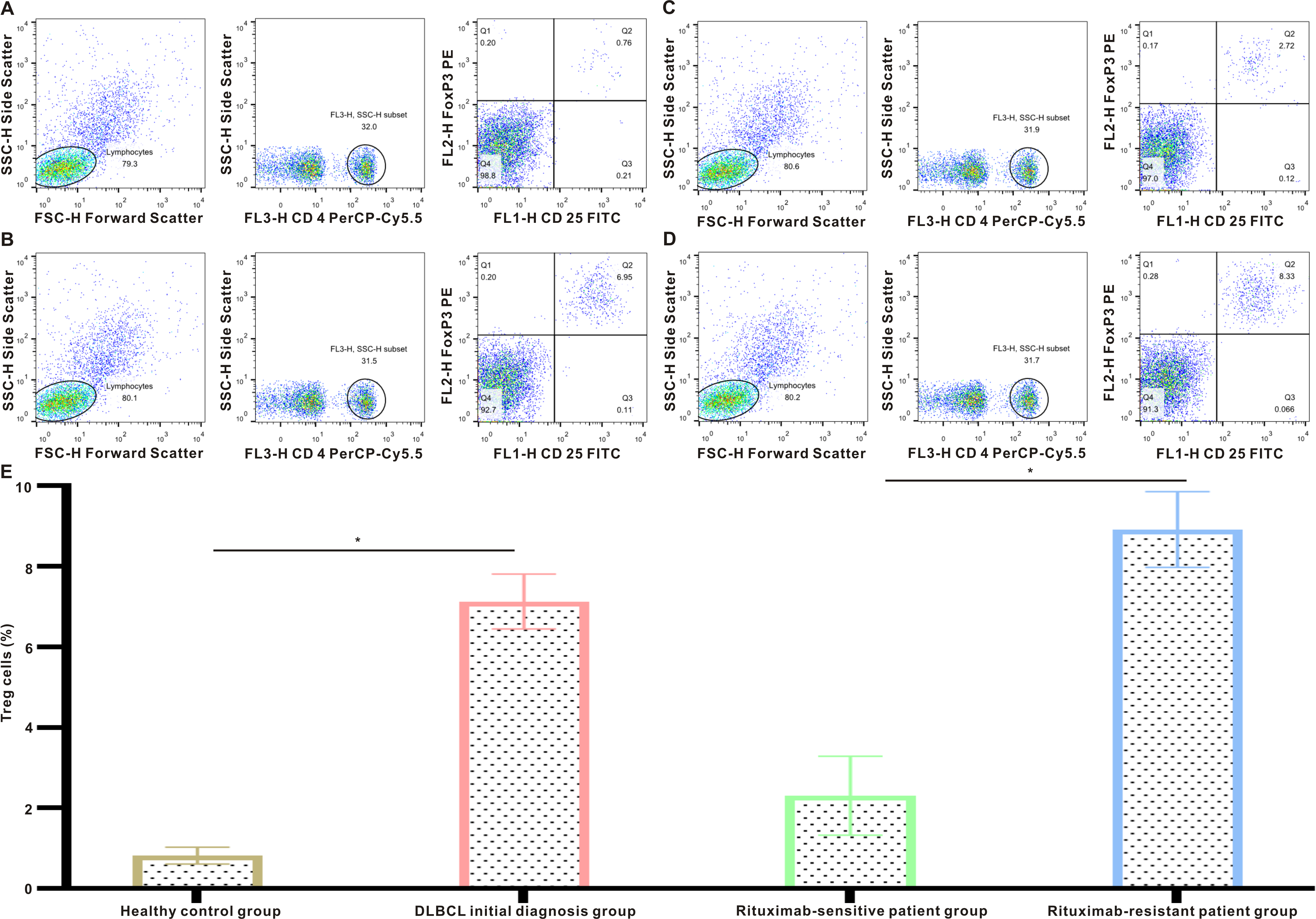


**Supplementary Figure 24 A-E.** (A-D) Flow cytometry measurements of the proportion of Tregs (CD4^+^CD25^+^Foxp3^+^) in the peripheral blood of the normal control group, newly diagnosed DLBCL group, rituximab-sensitive group, and rituximab-resistant group. (E) Statistical analysis of Treg cells in healthy human controls and human DLBCL patients. Data are expressed as the average ± SD (n = 3). *p < 0.05.

## 2.25 Detection of Cytokines in Mouse Serum


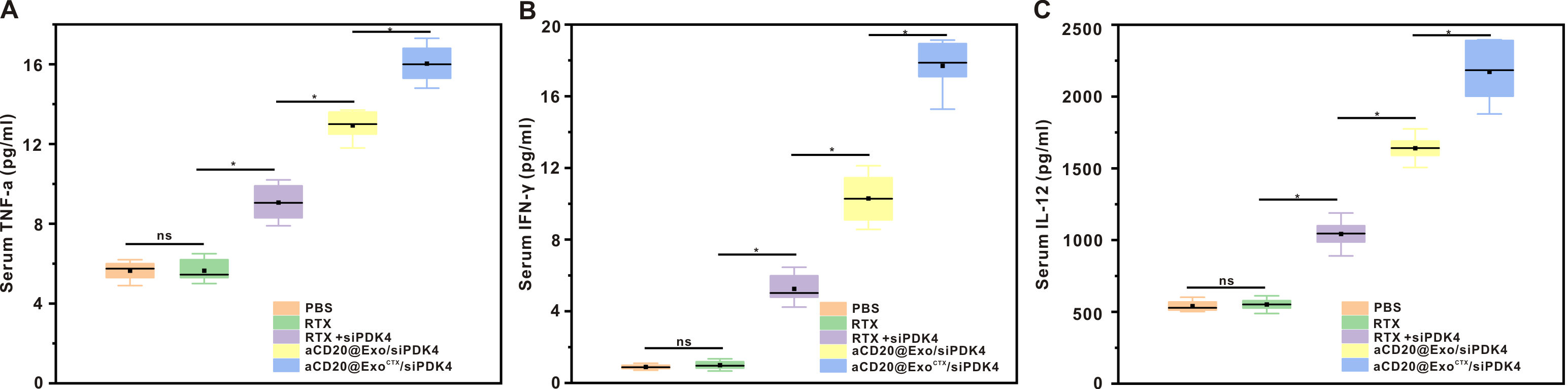


**Supplementary Figure 25 A-C.** Cytokine levels in sera from mice isolated from different treatments. Data shown are mean ± SD (n = 3). *p < 0.05, ns, no significant difference.

## 2.26 H&E Staining of Key Organs in Mice

**Supplementary Figure 26.** The main organs were stained with H&E. Scale bar: 50 μm.

## 2.27 Determining the potential inflammatory effects of aCD20@Exo^CTX^/siPDK4 through the levels of interleukin-6 (IL-6) in plasma.


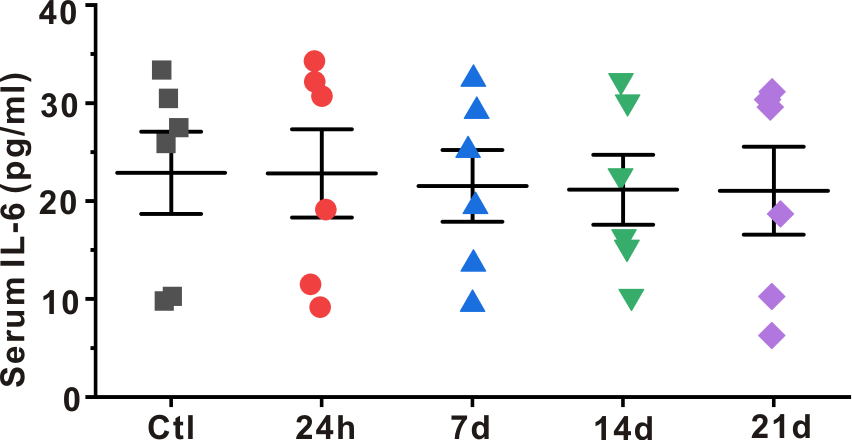


**Supplementary Figure 27.** in mouse plasma 24 h, 7, 14, and 21 days after aCD20@Exo^CTX^/siPDK4 via vehicle injection for the control group (PBS). Data represent the mean ± SEM of 6 mice.

## 2.28 Hematological and biochemical tests of mouse blood.


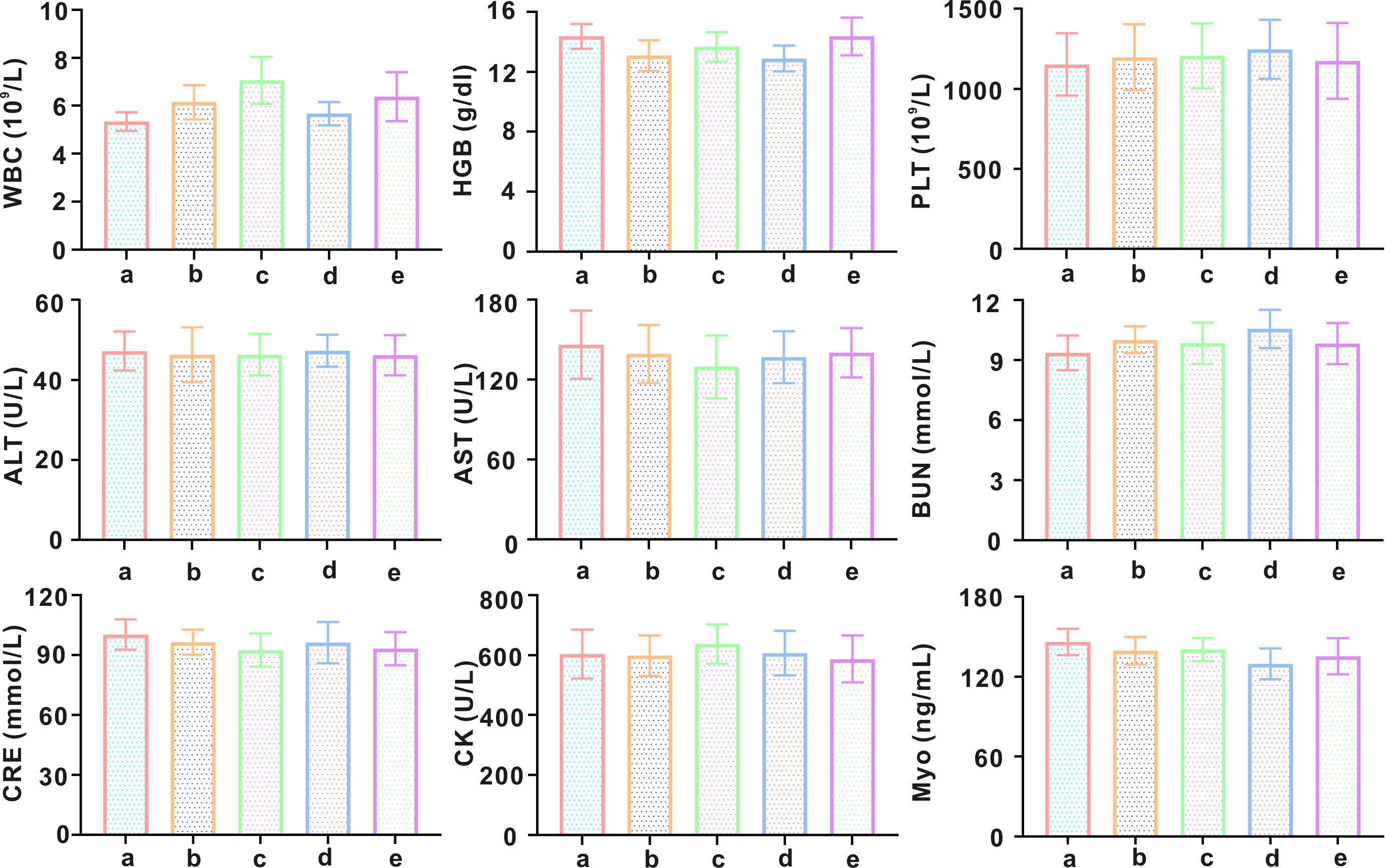


**Supplementary Figure 28.** Blood routine indexes (WBC, HGB, and PLT), Liver function index (ALT, AST), Renal function index (BUN, CRE), and Cardiac function index (CK, Myo) were detected.

# 3. References

(1) Morris, G. M.; Huey, R.; Olson, A. J. Using AutoDock for ligand-receptor docking. *Curr Protoc Bioinformatics* 2008, ***Chapter 8***, Unit 8 14. DOI: 10.1002/0471250953.bi0814s24.

(2) Pierce, B. G.; Wiehe, K.; Hwang, H.; Kim, B. H.; Vreven, T.; Weng, Z. ZDOCK server: interactive docking prediction of protein-protein complexes and symmetric multimers. *Bioinformatics* 2014, ***30*** (12), 1771-1773. DOI: 10.1093/bioinformatics/btu097.

(3) Pierce, B. G.; Hourai, Y.; Weng, Z. Accelerating protein docking in ZDOCK using an advanced 3D convolution library. *PLoS One* 2011, ***6*** (9), e24657. DOI: 10.1371/journal.pone.0024657.

(4) Zohni, K.; Lopez, L.; Mander, P.; Szaraz, P.; Filice, M.; Wyse, B. A.; et al. Human umbilical cord perivascular cells maintain regenerative traits following exposure to cyclophosphamide. *Cancer Lett* 2021, ***501***, 133-146. DOI: 10.1016/j.canlet.2020.12.035.

(5) Xu, P.; Zhang, X.; Chen, K.; Zhu, M.; Jia, R.; Zhou, Q.; et al. Tumor cell–derived microparticles induced by methotrexate augment T-cell antitumor responses by down-regulating expression of PD-1 in neutrophils. *Cancer Immunology Research* 2023, CIR-22-0595.

(6) Wu, S.; Luo, M.; To, K. K. W.; Zhang, J.; Su, C.; Zhang, H.; et al. Intercellular transfer of exosomal wild type EGFR triggers osimertinib resistance in non-small cell lung cancer. *Mol Cancer* 2021, ***20*** (1), 17. DOI: 10.1186/s12943-021-01307-9.

(7) Roider, T.; Baertsch, M. A.; Fitzgerald, D.; Vöhringer, H.; Brinkmann, B. J.; Czernilofsky, F.; et al. Multimodal and spatially resolved profiling identifies distinct patterns of T cell infiltration in nodal B cell lymphoma entities. *Nat Cell Biol* 2024, ***26*** (3), 478-489. DOI: 10.1038/s41556-024-01358-2 PubMed.

(8) Roider, T.; Seufert, J.; Uvarovskii, A.; Frauhammer, F.; Bordas, M.; Abedpour, N.; et al. Dissecting intratumour heterogeneity of nodal B-cell lymphomas at the transcriptional, genetic and drug-response levels. *Nat Cell Biol* 2020, ***22*** (7), 896-906. DOI: 10.1038/s41556-020-0532-x PubMed.

(9) Steen, C. B.; Luca, B. A.; Esfahani, M. S.; Azizi, A.; Sworder, B. J.; Nabet, B. Y.; et al. The landscape of tumor cell states and ecosystems in diffuse large B cell lymphoma. *Cancer Cell* 2021, ***39*** (10). DOI: 10.1016/j.ccell.2021.08.011 PubMed.

(10) Lenz, G.; Wright, G.; Dave, S. S.; Xiao, W.; Powell, J.; Zhao, H.; et al. Stromal gene signatures in large-B-cell lymphomas. *The New England Journal of Medicine* 2008, ***359*** (22), 2313-2323. DOI: 10.1056/NEJMoa0802885 PubMed.

(11) Ye, X.; Wang, L.; Nie, M.; Wang, Y.; Dong, S.; Ren, W.; et al. A single-cell atlas of diffuse large B cell lymphoma. *Cell Rep* 2022, ***39*** (3), 110713. DOI: 10.1016/j.celrep.2022.110713 From NLM.

(12) Maeser, D.; Gruener, R. F.; Huang, R. S. oncoPredict: an R package for predicting in vivo or cancer patient drug response and biomarkers from cell line screening data. *Brief Bioinform* 2021, ***22*** (6). DOI: 10.1093/bib/bbab260 PubMed.

(13) Newman, A. M.; Steen, C. B.; Liu, C. L.; Gentles, A. J.; Chaudhuri, A. A.; Scherer, F.; et al. Determining cell type abundance and expression from bulk tissues with digital cytometry. *Nat Biotechnol* 2019, ***37*** (7), 773-782. DOI: 10.1038/s41587-019-0114-2 PubMed.

(14) Jin, S.; Guerrero-Juarez, C. F.; Zhang, L.; Chang, I.; Ramos, R.; Kuan, C.-H.; et al. Inference and analysis of cell-cell communication using CellChat. *Nature Communications* 2021, ***12*** (1), 1088. DOI: 10.1038/s41467-021-21246-9 PubMed.

(15) Qiu, X.; Mao, Q.; Tang, Y.; Wang, L.; Chawla, R.; Pliner, H. A.; et al. Reversed graph embedding resolves complex single-cell trajectories. *Nat Methods* 2017, ***14*** (10), 979-982. DOI: 10.1038/nmeth.4402 PubMed.
